# Supplementary material for: Biochemical and structural insights of multifunctional flavin-dependent monooxygenase FlsO1-catalyzed unexpected xanthone formation
Source: Nat Commun. 2022 Sep 14;13:5386. doi: 10.1038/s41467-022-33131-0 (PMC9474520; doi:10.1038/s41467-022-33131-0)
Supplement: Supplementary file 1 — Supplementary Information [file 41467_2022_33131_MOESM1_ESM.pdf]

Supplementary Information

Biochemical and structural insights of multifunctional flavin-dependent monooxygenase FlsO1-catalyzed unexpected xanthone formation

Chunfang Yang[1,2,3,4,#], Liping Zhang[1,2,3,#], Wenjun Zhang[1,2,3,4], Chunshuai Huang[1,4], Yiguang Zhu[1,2,3,4], Xiaodong Jiang[1,4], Wei Liu[1], Mengran Zhao[1,4], Bidhan Chandra De[1,4], Changsheng Zhang[1,2,3,4]

[1] Key Laboratory of Tropical Marine Bioresources and Ecology, Guangdong Key Laboratory of Marine Materia Medica, South China Sea Institute of Oceanology, Chinese Academy of Sciences, Guangzhou 510301, China

[2] Southern Marine Science and Engineering Guangdong Laboratory (Guangzhou), 1119 Haibin Road, Nansha District, Guangzhou 511458, China;

[3] Sanya Institute of Ocean Eco-Environmental Engineering, Yazhou Scientific Bay, Sanya 572000, China

[4] University of Chinese Academy of Sciences, 19 Yuquan Road, Beijing 100049, China

[#] These authors contributed equally.

Correspondence should be addressed to Changsheng Zhang (Email: czhang@scsio.ac.cn or czhang2006@gmail.com; orcid.org/0000-0003-2349-3138).

Table of Contents

Supplementary Notes 5
1.1. Structure elucidation........ 5
1.2. Spectral data for new compounds........ 10

Supplementary Tables 13
Supplementary Table 1. Strains and plasmids used and constructed in this study........ 13
Supplementary Table 2. Primers used in this study. 13
Supplementary Table 3. ^{13}C NMR data for compounds **16**–**19**, **22**, **24** and **25**........ 15
Supplementary Table 4. ^{1}H NMR data for compounds **16**–**19**, **22**, **24** and **25**........ 16
Supplementary Table 5. ^{1}H NMR (700 MHz) data and ^{13}C (176 MHz) NMR data for compounds **26** and **27** in acetone-d_6 17
Supplementary Table 6. Data collection of FlsO1/FAD and refinement statistics........ 18

Supplementary Figures 19
Supplementary Fig. 1. Representative methods for the chemical synthesis of the xanthone skeleton.19
Supplementary Fig. 2. SDS-PAGE analysis of FlsO1 and its homologous proteins and determination of the associated cofactors........ 20
Supplementary Fig. 3. HPLC analysis of enzyme reactions of FlsO1, AlpK, and Nes26 with PJM (**8**). 21
Supplementary Fig. 4. HRESIMS spectrum of fluoxanthone A (**16**)........ 22
Supplementary Fig. 5. The ^{1}H NMR (700 MHz) spectrum of fluoxanthone A (**16**) and its partial ^{1}H NMR from ppm 6.8 to ppm 8.0 in acetone-d_6/DMSO-d_6 (20:1). 23
Supplementary Fig. 6. The ^{13}C NMR (176 MHz) spectrum of fluoxanthone A (**16**) in acetone-d_6/DMSO-d_6 (20:1)........ 24
Supplementary Fig. 7. The DEPT 135 spectrum of fluoxanthone A (**16**) in acetone-d_6/DMSO-d_6 (20:1). 25
Supplementary Fig. 8. The HSQC spectrum of fluoxanthone A (**16**) in acetone-d_6/DMSO-d_6 (20:1).26
Supplementary Fig. 9. The COSY spectrum of fluoxanthone A (**16**) in acetone-d_6/DMSO-d_6 (20:1).27
Supplementary Fig. 10. The HMBC spectrum of fluoxanthone A (**16**) in acetone-d_6/DMSO-d_6 (20:1). 28
Supplementary Fig. 11. HRESIMS spectrum of fluoxanthone B (**17**)........ 29
Supplementary Fig. 12. The ^{1}H NMR (700 MHz) spectrum of fluoxanthone B (**17**) in acetone-d_6.... 30
Supplementary Fig. 13. The ^{13}C (176 MHz) spectrum of fluoxanthone B (**17**) in acetone-d_6........ 31
Supplementary Fig. 14. The DEPT 135 spectrum of fluoxanthone B (**17**) in acetone-d_6. 32
Supplementary Fig. 15. The HSQC spectrum of fluoxanthone B (**17**) in acetone-d_6........ 33
Supplementary Fig. 16. The COSY spectrum of fluoxanthone B (**17**) in acetone-d_6........ 34
Supplementary Fig. 17. The HMBC spectrum of fluoxanthone B (**17**) in acetone-d_6........ 35
Supplementary Fig. 18. HRESIMS spectrum of fluoxanol (**18**). 36
Supplementary Fig. 19. The ^{1}H NMR (700 MHz) spectrum of fluoxanol (**18**) in DMSO-d_6........ 37
Supplementary Fig. 20. The ^{13}C (176 MHz) spectrum of fluoxanol (**18**) in DMSO-d_6. 38
Supplementary Fig. 21. The DEPT 135 spectrum of fluoxanol (**18**) in DMSO-d_6........ 39
Supplementary Fig. 22. The HSQC spectrum of fluoxanol (**18**) in DMSO-d_6. 40
Supplementary Fig. 23. The COSY spectrum of fluoxanol (**18**) in DMSO-d_6. 41
Supplementary Fig. 24. The HMBC spectrum of fluoxanol (**18**) in DMSO-d_6. 42
Supplementary Fig. 25. Comparison of the experimental ECD spectra of **8** and **17**. 43
Supplementary Fig. 26. HPLC analysis of the time course assay of FlsO1 with PJM (**8**). 44
Supplementary Fig. 27. LC-MS analysis for the putative intermediated of the FlsO1 reaction with **8**. 45
Supplementary Fig. 28. HRESIMS spectrum of 12-hydroxyl-prejadomycin (**19**). 46
Supplementary Fig. 29. The ^{1}H NMR (700 MHz) spectrum of 12-hydroxyl-prejadomycin (**19**) in DMSO-d_6........ 47
Supplementary Fig. 30. The ^{13}C (176 MHz) spectrum of 12-hydroxyl-prejadomycin (**19**) in DMSO-d_6. 48
Supplementary Fig. 31. The DEPT 135 spectrum of 12-hydroxyl-prejadomycin (**19**) in DMSO-d_6.. 49

Supplementary Fig. 32. The HSQC spectrum of 12-hydroxyl-prejadomycin (**19**) in DMSO-d_6.........50
Supplementary Fig. 33. The COSY spectrum of 12-hydroxyl-prejadomycin (**19**) in DMSO-d_6.........51
Supplementary Fig. 34. The HMBC spectrum of 12-hydroxyl-prejadomycin (**19**) in DMSO-d_6. 52
Supplementary Fig. 35. The stability of **17** under diverse conditions. 53
Supplementary Fig. 36. The stability of **18** in diverse solvents and the spontaneous conversation of **18** to **16**. 54
Supplementary Fig. 37. HPLC analysis of the reaction of **22** with DTC (**23**) and the MS analysis of the captured product **24**. 55
Supplementary Fig. 38. HRESIMS spectrum of DTC-fluostacid A (**24**). 56
Supplementary Fig. 39. The ^{1}H NMR (700 MHz) spectrum of DTC-fluostacid A (**24**) in DMSO-d_6.57
Supplementary Fig. 40. The ^{13}C (176 MHz) spectrum of DTC-fluostacid A (**24**) in DMSO-d_6. 58
Supplementary Fig. 41. The HSQC spectrum of DTC-fluostacid A (**24**) in DMSO-d_6. 59
Supplementary Fig. 42. The COSY spectrum of DTC-fluostacid A (**24**)in DMSO-d_6. 60
Supplementary Fig. 43. The HMBC spectrum of DTC-fluostacid A (**24**) in DMSO-d_6. 61
Supplementary Fig. 44. HRESIMS spectrum of DTC-fluostacid B (**25**). 62
Supplementary Fig. 45. The ^{1}H NMR (700 MHz) spectrum of DTC-fluostacid B (**25**) in acetone-d_6. 63
Supplementary Fig. 46. The ^{13}C (176 MHz) spectrum of DTC-fluostacid B (**25**) in acetone-d_6.........64
Supplementary Fig. 47. The DEPT 135 spectrum of DTC-fluostacid B (**25**) in acetone-d_6. 65
Supplementary Fig. 48. The HSQC spectrum of DTC-fluostacid B (**25**) in acetone-d_6.......................66
Supplementary Fig. 49. The COSY spectrum of DTC-fluostacid B (**25**) in acetone-d_6.......................67
Supplementary Fig. 50. The HMBC spectrum of DTC-fluostacid B (**25**) in acetone-d_6......................68
Supplementary Fig. 51. The HRESIMS spectrum of epoxy-prejadomycin (**22**). 69
Supplementary Fig. 52. The ^{1}H NMR (700 MHz) spectrum of epoxy-prejadomycin (**22**) in acetone-d_6. 70
Supplementary Fig. 53. The HSQC spectrum of epoxy-prejadomycin (**22**) in acetone-d_6. 71
Supplementary Fig. 54. The HMBC spectrum of epoxy-prejadomycin (**22**) in acetone-d_6. 72
Supplementary Fig. 55. Comparison of the the HMBC spectrum of **22** and **19**. 73
Supplementary Fig. 56. The proposed mechanism for the formation of **24**. 74
Supplementary Fig. 57. LC-MS analysis of the FlsO1 reactions terminated with normal and deuterated MeOH. 75
Supplementary Fig. 58. HRESIMS spectrum of epoxyfluoxanester B (**26**)..76
Supplementary Fig. 59. The ^{1}H NMR (700 MHz) spectrum of epoxyfluoxanester B (**26**) in acetone-d_6. 77
Supplementary Fig. 60. The ^{13}C (176 MHz) spectrum of epoxyfluoxanester B (**26**) in acetone-d_6. ...78
Supplementary Fig. 61. The DEPT 135 spectrum of epoxyfluoxanester B (**26**) in acetone-d_6...........79
Supplementary Fig. 62. The HSQC spectrum of epoxyfluoxanester B (**26**) in acetone-d_6. 80
Supplementary Fig. 63. The COSY spectrum of epoxyfluoxanester B (**26**) in acetone-d_6. 81
Supplementary Fig. 64. The HMBC spectrum of epoxyfluoxanester B (**26**) in acetone-d_6.................82
Supplementary Fig. 65. HRESIMS spectrum of epoxyfluoxanester A (**27**). 83
Supplementary Fig. 66. The ^{1}H NMR (700 MHz) spectrum of epoxyfluoxanester A (**27**) in acetone-d_6. 84
Supplementary Fig. 67. The ^{13}C (176 MHz) spectrum of epoxyfluoxanester A (**27**) in acetone-d_6. ...85
Supplementary Fig. 68. The DEPT 135 spectrum of epoxyfluoxanester A (**27**) in acetone-d_6. 86
Supplementary Fig. 69. The HSQC spectrum of epoxyfluoxanester A (**27**) in acetone-d_6.................87
Supplementary Fig. 70. The COSY spectrum of epoxyfluoxanester A (**27**) in acetone-d_6.................88
Supplementary Fig. 71. The HMBC spectrum of epoxyfluoxanester A (**27**) in acetone-d_6.................89
Supplementary Fig. 72. Comparison of the experimental and calculated ECD spectra of **26** and **27**. 90
Supplementary Fig. 73. LC-HRMS analysis of the spontaneous conversion of **21** to **18**. 91
Supplementary Fig. 74. HPLC analysis of the FlsO1 reaction with **19** and **22** as the substrate. 92
Supplementary Fig. 75. LC-MS analysis of products from FlsO1 reactions with **8** in atmospheric $^{16}O_2$ or $^{18}O_2$. 93
Supplementary Fig. 76. The proposed catalytic mechanism for the FPMO XanO4. 94
Supplementary Fig. 77. The comparison of mechanisms for FPMOs-catalyzed ring opening reaction. 95

Supplementary Fig. 78. The proposed involvement of two forms of reactive flavin species hydroperoxyflavin (FADOOH) or a peroxyflavin ($FADOO^-$) in a single enzyme-catalyzed reactions.96
Supplementary Fig. 79. Determination of kinetic parameters of FlsO1 and FlsO2 reactions with PJM (**8**)....97
Supplementary Fig. 80. HPLC analysis of the production profiles of *M. rosaria* SCSIO N160 and the Δ*flsO2* mutant. 98
Supplementary Fig. 81. Comparison of UV-Vis spectra of **44** and **45** with those of known compounds. 99
Supplementary Fig. 82. LC-HRMS/MS analysis of **44** and **45**. 100
Supplementary Fig. 83. LC-MS analysis of the products **44** and **45** from the FlsO1 reaction with NEN C (**14**) in the presence of ^{14}N-L-cysteine or ^{15}N-L-cysteine. 101
Supplementary Fig. 84. LC-MS analysis of the **44** and **45** of the FlsO1 reaction with **14** in atmospheric $^{16}O_2$ or $^{18}O_2$. 102
Supplementary Fig. 85. The proposed mechanism for the formation of **44** and **45**. 103
Supplementary Fig. 86. *In vitro* Characterization of the FlsO1 homologous enzymes AlpK and Nes26 using NEN C (**14**) as a substrate mimic. 104
Supplementary Fig. 87. Structure comparison of FlsO1 with its homologues AlpK, MtmOIV, and BexE. 105
Supplementary Fig. 88. Alignments of the docked models of **8**, **20** and **22**. 106
Supplementary Fig. 89. The SDS-PAGE analysis of purified recombinant proteins of FlsO1 and its mutants. 107
Supplementary Fig. 90. Phylogenetic analysis of FlsO1. 108
Supplementary Fig. 91. Characterization of the catalytic activity of FlsO1 Q108G/R109G mutant. 109

Supplementary References 110

Supplementary Notes

1.1. Structure elucidation

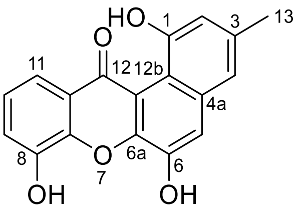

fluoxanthone A (**16**)

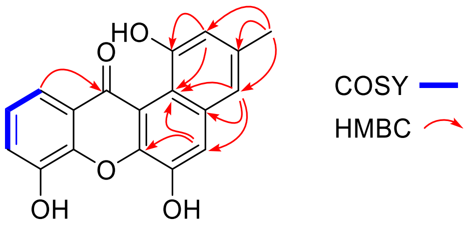

fluoxanthone A (**16**)

Fluoxanthone A (**16**) was obtained as light-yellow powders. The molecular formula of **16** was assigned as $C_{18}H_{12}O_5$ based on HRMS (ESI-TOF) *m/z* 307.0612 [M - H]$^-$ (calcd 307.0612), (Supplementary Fig. 4), corresponding to 13 degrees of unsaturation. ^{1}H and ^{13}C NMR data of **16** were highly similar to those of DHR (**9**) (Supplementary Figs. 5–7).[1] The difference was that **16** had a pyran-4-one moiety, instead of the 1,4-benzoquinone unit in DHR (**9**). The presence of the pyran-4-one moiety in **16** was supported by the deshielded resonances of C-6a (δ_C 145.5, Δ 27.5 ppm), and C-7a (δ_C 143.7, Δ 29.2 ppm), as well as one less degree of unsaturation.[1] Finally, the structure of **16** was confirmed by detailed assignment of 2D NMR data (Supplementary Figs. 8–10).

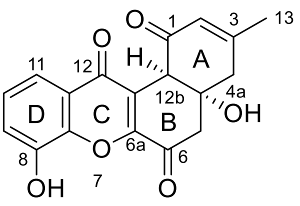

fluoxanthone B (**17**)

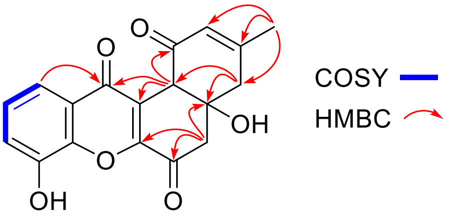

fluoxanthone B (**17**)

Fluoxanthone B (**17**) was obtained as yellow powder. The molecular formula of **17** was established to be $C_{18}H_{14}O_6$ by HRMS (ESI-TOF) *m/z* 325.0725 [M - H]$^-$ (calcd 325.0718) (Supplementary Fig. 11), requiring 12 degrees of unsaturation. ^{1}H and ^{13}C NMR data of **17** were similar to those of PJM (**8**) with the differences in the ring C (Supplementary Figs. 12 and 13). The presence of a pyran-4-one ring in **17** rather than the phenol moiety in **8** was supported by the conjugated keto group (δ_C 178.1), and the deshielded resonances of C-6a (δ_C 149.5, Δ 40.4 ppm) and C-7a (δ_C 144.8, Δ 33.1 ppm) (Supplementary Figs. 14–17). These assignments were further supported by the HMBC correlations from H_2-5 to C-6a, H-9 to C-7a, and H-11 to

C-12 (Supplementary Fig. 17). Thus, the planar structure of **17** was determined. The stereochemistry at the C-4a and C-12b in **17** was assigned as 4a*R*,12b*R* given that **17** and **8** have almost identical Cotton effects of experimental ECD spectra (Supplementary Fig. 25).

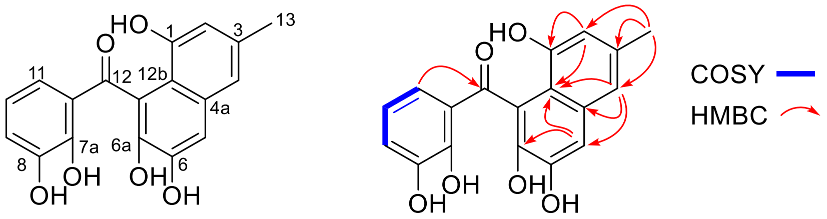

fluoxanol (**18**) fluoxanol (**18**)

Fluoxandiol (**18**) was obtained as yellow powder and the molecular formula of **18** was assigned as $C_{18}H_{14}O_6$ by HRMS (ESI-TOF) *m/z* 325.0726 $[M - H]^-$ (calcd 325.0718) (Supplementary Fig. 18), indicating 12 degrees of unsaturation. Detailed analysis of 1D and 2D NMR spectra of **18** revealed its highly structural similarity to **16** (Supplementary Figs. 19–24). In the ^{13}C NMR spectrum of **18**, an unusual keto group at δ_C 204.7 was observed, implying that the xanthone moiety in **16** was a C-ring-open form in **18**. This assignment was supported by the HMBC correlations of H-5 to C-6a, H-9 to C-7a, and H-11 to C-12 (Supplementary Fig. 24), as well as one less degree of unsaturation.

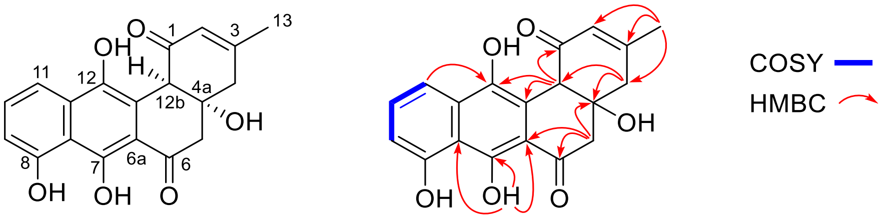

12-hydroxyl-prejadomycin (**19**) 12-hydroxyl-prejadomycin (**19**)

12-Hydroxy-prejadomycin (**19**) was obtained as yellow powders, and was assigned the molecular formula $C_{19}H_{16}O_6$ by HRMS (ESI-TOF) *m/z* 340.1091 $[M - H]^-$ (calcd 340.1020) (Supplementary Fig. 28). Careful analysis of ^1H, ^{13}C and 2D NMR data of **19** (Supplementary Figs. 29–34) revealed its was strikingly structural similarity to PJM (**8**). The only difference was that the H-12 in **8** was substituted by an additional hydroxy group in **19**. This assignment was supported by the HMBC correlations from 12-OH to C-12/C-11a/C-12a (Supplementary Fig. 34). Therefore, the structure of **19** was determined to be 12-hydroxy-prejadomycin.

Accordingly, the absolute configuration of **19** was assigned as 4a*R*,12b*R* given that it was a derivative of **8**.

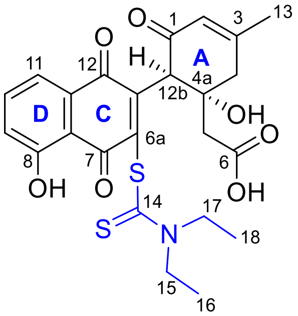

DTC-fluostacid A (**24**)

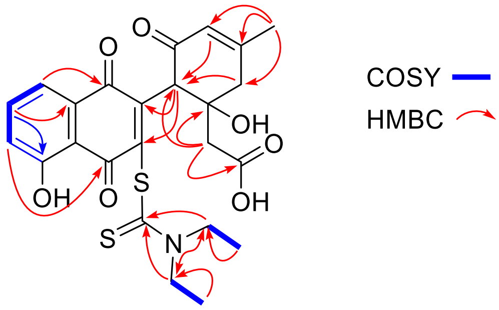

DTC-fluostacid A (**24**)

DTC-fluostacid A (**24**) was obtained as a red amorphous powder with a molecular formula of $C_{24}H_{26}NO_7S_2$ by HRMS (ESI-TOF) *m/z* 504.1139 $[M + H]^+$ (calcd 504.1145) (Supplementary Fig. 38), suggesting that **24** was a DTC (**23**) derivative. The 1D and 2D NMR spectra of **24** allowed the assignment of the unit DTC (Supplementary Figs. 39–43, Supplementary Tables 3 and 4). Careful inspection of the NMR data of **24** indicated the presence of a singlet methyl (δ_H 2.02, 3H, s), two methylenes, five methines including a sp^3 methine (δ_H 4.10, 1H, s), an olefinic methine (δ_H 5.98, 1H, s), and the characteristic aromatic ABC spin system (δ_H 7.37, 1H, d, J = 8.7 Hz; δ_H 7.77, 1H, dd, J = 8.7, 7.0 Hz; δ_H 7.51, 1H, d, J = 7.0 Hz), similar to those of PJM (**8**)[1]. The ^1H-^1H COSY and HMBC correlations allowed the construction of the rings A and D, which were the same as those in PJM (**8**) (Supplementary Figs. 42 and 43). In addition to signals for these fragments, the remaining ^{13}C NMR data of **24** showed 6 carbon resonances for an sp^3 methylene carbon, and five quaternary carbons including two olefinic ones and three carbonyl carbons (δ_C 183.7; δ_C 181.8; δ_C 172.7). The HMBC correlations from H-9 to C-7 (δ_C 183.7), H-11 to C-12 (δ_C 181.8), and H-12b to C-6a/C-12a assigned the two olefinic quaternary carbons and two ketos as 1,4-benzoquinone unit (the ring C). The sp^3 methylene and the additional carbonyl quaternary carbon (δ_C 172.7) were assigned to an acetic acid side chain and located at C-4a, which were supported by the HMBC correlations from H_2-5 to C-4/C-4a/C-12b, indicating the B-ring-open form in **24**. The DTC unit was attached to C-6a by detailed assignment NMR data of **24**. Thus, the planar structure of **24** was assigned as DTC-substituted naphthalenedione-oxocyclohex-acetic acid.

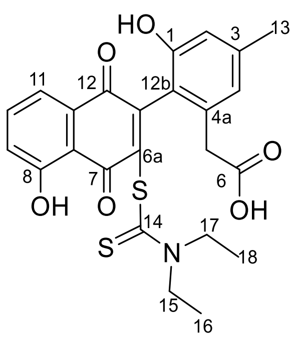

DTC-fluostacid B (**25**)

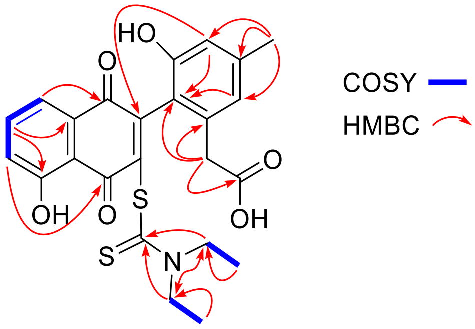

DTC-fluostacid B (**25**)

DTC-fluostacid B (**25**) was obtained as yellow powders, and the molecular formula of **25** was established to be $C_{24}H_{23}NO_6S_2$ by HRMS (ESI-TOF) *m/z* 486.1024 $[M + H]^+$ (calcd 486.1040) (Supplementary Fig. 44), 18 mass units less than that of **24**. Analysis of ^{1}H, ^{13}C, and 2D NMR data revealed that **25** was only different from **24** by aromatization of the ring A, which was supported by the HMBC correlations of H_3-13 to C-2/C-3, H-2 to C-1/C-12b, and H-4 to C-4a/C-12b (Supplementary Fig. 45–50). Thus the structure of **25** was assigned as DTC-substituted dioxo-dihydronaphthanlen-acetic acid (**25**).

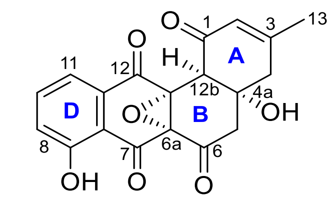

epoxy-prejadomycin (**22**)

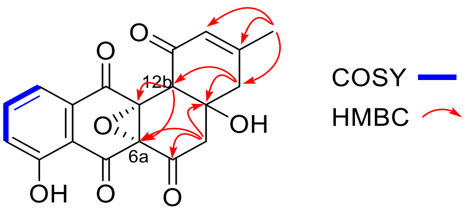

epoxy-prejadomycin (**22**)

Epoxyprejadomycin (**22**) was obtained as light-yellow powders. The molecular formula of **22** was determined as $C_{19}H_{15}O_7$ by HRMS (ESI-TOF) (*m/z* $[M + H]^+$ calcd for 355.0812, found 355.0813) (Supplementary Fig. 51). Careful analysis of ^{1}H, HSQC, and HMBC NMR data of **22** indicated the presence of a singlet methyl (δ_H 2.07, 3H, s), two pairs of methylenes, a sp^3 methine, and characteristic aromatic ABC spin system (δ_H 7.43, 1H, d, J = 7.3 Hz; δ_H 7.88, 1H, dd, J = 7.3, 8.1 Hz; δ_H 7.74, 1H, d, J = 8.1 Hz), which suggested that the ring A, ring B, and ring D of **22** were the same as those of **19** (Supplementary Fig. 52–54). These suggestions were supported by HMBC correlations of H-12 to C-2/C-3/C-4, H-4 to C-12/C-2/C-3/C-4a/C-5/C-12b, H-5 to C-6, H-9 to C-7a/C-11, H-10 to C-8/C-11a and H-11 to C-7a/C-9. The epoxide moiety was located at C-6a/C-12a by HMBC correlations of H-12b to C-6a (Supplementary Fig. 54). Thus, the structure of **22** was tentatively assigned to be 6a,12a-epoxy-7,12-dione-prejadomycin. On the consideration of **22** as a biosynthetic precursor of **27**, the absolute configuration of **22** was tentatively assigned as 4a*R*,6a*R*,12a*R*,12b*S*, the same as **27**.

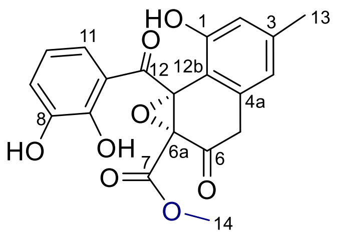

epoxyfluoxanester B (**26**)

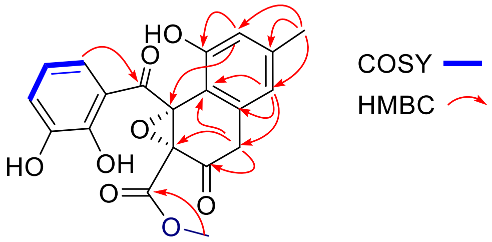

epoxyfluoxanester B (**26**)

Epoxyfluoxanester B (**26**) was established to be $C_{20}H_{16}O_8$ by HRMS (ESI-TOF) (*m/z* [M + H]$^+$, calcd 385.0918, found 385.0923), 18 mass units less than that of **27** (Supplementary Fig. 58). Analysis of ^{1}H and ^{13}C, and 2D NMR data (Supplementary Figs. 59–64) revealed that **26** was only different from **27** by aromatization of the ring A. These assigned were supported by the HMBC correlations from H_3-13 to C-2/C-4, H-2/H-4 to C-12b, and H_2-5 to C-4a/H-12b. Detailed assignment of 2D NMR data confirmed the structure of **26**. Thus, the planar structure of **26** was determined. The absolute configuration of **26** was assigned as (6a*R*,12a*R*) by comparison the experimental ECD spectrum of **26** with computed TDDFT-ECD spectrum of (6a*R*,12a*R*)-**26** (Supplementary Fig. 72).

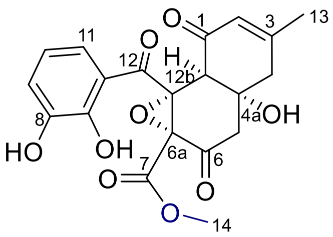

epoxyfluoxanester A (**27**)

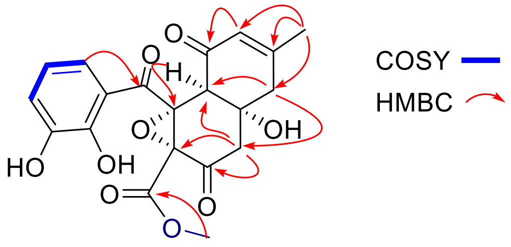

epoxyfluoxanester A (**27**)

Epoxyfluoxanester A (**27**) was established to be $C_{20}H_{18}O_9$ by HRMS (ESI-TOF) (*m/z* [M + H]$^+$, calcd 403.1024, found 403.1030), requiring 12 degrees of unsaturation (Supplementary Fig. 65). ^{1}H and ^{13}C NMR data of **27** (Supplementary Figs. 66 and 67) were similar to those of fluoxanthone B (**17**). The differences were presence of one methoxy (δ_H 3.43, δ_C 52.0), two sp^3 hybridized non-protonated oxygen-bearing carbons (δ_C 81.4; δ_C 87.7), and one ester carbonyls (δ_C 167.7) in **27**, which were suggested as a methyl ester moiety and an epoxy group. These suggestions were supported by the HMBC correlations from H_3-14 to C-7, H_2-5 to C-6a, H-12b to C-12a (Supplementary Fig. 71). Detailed assignment of 2D NMR data (Supplementary Figs.

69–71) indicated the ring C of **17** opened in **27**, and located the methyl ester unit at C6a and the epoxy group at C-6a/C-12a, respectively. Thus, the planar structure of **27** was determined. The absolute configuration at the C-4a and C-12b of **27** was assigned as 4a*R*,12b*S* given that it was a derivative of **8**. The (6a*R*,12a*R*) absolute configuration was assigned for **27** by means of the good agreement between the experimental ECD spectrum of **27** and computed TDDFT-ECD spectrum of (4a*R*,6a*R*,12a*R*,12b*S*)-**27** (Supplementary Fig. 72).

1.2. Spectral data for new compounds

Fluoxanthone A (**16**). ^{1}H NMR (700 MHz, acetone-d_6 /DMSO-d_6 20:1): δ 12.9 (s, 1H), 7.85 (dd, *J* = 7.9, 1.6 Hz, 1H), 7.64 (s, 1H), 7.43 (t, *J* = 7.9 Hz, 1H), 7.41 (dd, *J* = 7.9, 1.6 Hz, 1H), 7.12 (d, *J* = 1.5 Hz, 1H), 6.84 (d, *J* = 1.5 Hz, 1H), 2.40 (s, 3H); ^{13}C NMR (176 MHz, acetone-d_6 /DMSO-d_6 20:1): δ 180.8, 156.1, 150.1, 146.5, 144.8, 143.2, 138.1, 134.1, 125.5; 122.7, 120.7, 119.7, 118.5, 116.5, 116.1, 116.0, 113.2, 20.2; HRMS (*m*/*z*): [M - H]$^-$ calcd. for $C_{18}H_{11}O_5$, 307.0612; found 307.0612; analysis (calcd., found for $C_{18}H_{12}O_5$): C (70.13), H (3.92), O (25.95).

Fluoxanthone B (**17**). ^{1}H NMR (700 MHz, acetone-d_6): δ 7.59 (dd, *J* = 4.8, 4.8, 1H), 7.35 (d, *J* = 4.8 Hz, 2H), 6.01 (s, 1H), 4.35 (d, *J* = 2.2, 1H); 3.09 (d, *J* = 19.2 Hz, 1H), 3.08 (d, *J* = 17.8, 1H), 2.73 (dd, *J* = 17.8, 2.2 Hz, 1H), 2.70 (d, *J* = 19.2 Hz, 1H), 2.08 (s, 3H); ^{13}C NMR (176 MHz, acetone-d_6): δ 193.7, 191.8, 178.1, 159.5, 149.5, 147.5, 144.8, 125.7, 125.6, 125.1, 124.1, 120.4, 115.1, 73.6, 51.6, 47.0, 45.2, 23.0; HRMS (*m*/*z*): [M - H]$^-$ calcd. for $C_{18}H_{13}O_6$, 325.0718; found 325.0725; analysis (calcd., found for $C_{18}H_{14}O_6$): C (66.26), H (4.32), O (29.42).

Fluoxandiol (**18**). ^{1}H NMR (700 MHz, DMSO-d_6): δ 7.07 (s, 1H), 6.95 (dd, *J* = 7.1, 2.2 Hz, 1H), 6.89 (s, 1H), 6.55 (dd, *J* = 8.1, 7.1, 1H), 6.54 (dd, *J* = 8.1, 2.2 1H), 6.30 (s, 1H), 2.26 (s, 3H); ^{13}C NMR (176 MHz, DMSO-d_6): δ 204.7, 151.7, 150.1, 146.7, 146.3, 143.0,133.7, 130.7, 123.0, 122.9, 120.9, 110.0, 109.1, 118.5, 118.0, 116.7, 115.6, 21.6; HRMS (*m*/*z*): [M - H]$^-$ calcd. for $C_{18}H_{13}O_6$, 325.0718; found 325.0726; analysis (calcd., found for $C_{18}H_{14}O_6$): C (66.26), H (4.32), O (29.42).

12-Hydroxy-prejadomycin (**19**). ^{1}H NMR (700 MHz, DMSO-d_6): δ 15.37 (s, 1H), 9.88 (s, 1H), 8.68 (s, 1H), 7.62 (dd, *J* = 8.4, 1.1, 1H), 7.58 (dd, *J* = 8.4, 7.6 Hz, 1H), 6.88 (dd, *J* = 7.6, 1.1 1H), 5.99 (s, 1H), 5.63 (s, 1H), 4.35 (d, *J* = 2.4, 1H), 2.91 (d, *J* = 17.6Hz, 1H), 2.87 (d, *J* = 18.9, 1H), 2.53 (d, *J* = 18.9, 1H), 2.52 (dd, *J* = 17.6, 2.4, 1H), 2.02 (s, 3H); ^{13}C NMR (176 MHz,

DMSO-d_6): δ 204.1,195.6, 160.1, 158.3, 157.9, 143.8, 133.3, 132.1, 125.7, 115.3, 113.7, 113.1, 111.7; 109.1, 72.6, 53.6, 45.9, 45.9, 24.2; HRMS (m/z): [M - H]$^-$ calcd. for $C_{19}H_{15}O_6$, 340.1020; found, 340.1091; analysis (calcd., found for $C_{19}H_{16}O_6$): C (67.06), H (4.74), O (28.21).

DTC-fluostacid A (**24**). ^{1}H NMR (700 MHz, DMSO-d_6): δ 7.77 (dd, J = 8.7, 7.0 Hz, 1H), 7.51 (dd, J = 7.0, 1H), 7.37 (d, J = 8.7 1H), 5.98 (s, 1H), 4.10 (s, 1H), 3.93 (m, 2H), 3.85 (m, 2H), 2.87 (d, J = 16.6, 1H), 2.68 (d, J = 16.6, 1H), 2.56 (d, J = 14.6, 1H), 2.26 (d, J = 14.6 Hz, 1H), 2.02 (s, 3H), 1.42 (s, J = 6.9, 3H), 1.18 (t, J = 7.1, 3H). ^{13}C NMR (176 MHz, DMSO-d_6): δ 194.0, 190.7, 183.7, 181.8, 172.7, 160.1, 159.5, 148.7, 147.9, 136.5, 132.3, 125.0, 123.5, 118.9, 116.2, 74.8, 62.8, 48.7, 48.6, 42.9, 41.7, 24.3, 12.9. 11.4; HRMS (m/z): [M + H]$^+$ calcd. for $C_{24}H_{26}NO_7S_2$, 504.1145; found, 504.1139; analysis (calcd., found for $C_{24}H_{25}NO_7S_2$): C (57.24), H (5.00), N (2.78), O (22.24), S (12.73).

DTC-fluostacid B (**25**). ^{1}H NMR (700 MHz, acetone-d_6): δ 7.79 (t, J = 8.0 Hz, 1H), 7.64 (d, J = 8.0, 1H), 7.36 (d, J = 8.0 1H), 6.79 (s, 1H), 6.67 (s, 1H), 3.97 (m, 1H), 3.92 (m, 1H), 3.85 (m, 2H); 3.63 (d, J = 15.6 Hz, 1H), 3.45 (d, J = 15.6, 1H), 2.29 (s, 3H), 1.34 (t, J = 7.1, 3H), 1.23 (t, J = 7.1, 3H). ^{13}C NMR (176 MHz, acetone-d_6): δ 192.2, 186.3, 182.2, 172.1, 161.9, 155.7, 150.4, 145.7, 140.6, 137.5, 135.5, 133.7, 124.3; 123.2, 120.0, 119.8, 117.1, 115.5, 49.8, 49.5, 39.6, 21.4, 13.1, 11.6; HRMS (m/z): [M + H]$^+$ calcd. for $C_{24}H_{24}NO_6S_2$, 486.1040; found, 486.1024; analysis (calcd., found for $C_{24}H_{23}NO_7S_2$): C (59.37), H (4.77), N (2.88), O (19.77), S (13.21).

Epoxyprejadomycin (**22**). ^{1}H NMR (700 MHz, acetone-d_6): δ, 7.88 (dd, J = 8.1, 7.3 Hz, 1H), 7.74 (d, J = 8.1, 1H), 7.43 (d, J = 7.3 1H), 5.92 (s, 1H), 4.26 (d, J = 1.1, 1H), 2.96 (d, J = 17.0, 1H), 2.94 (d, J = 19.8, 1H), 2.79 (d, J = 17.0 Hz, 1H), 2.63 (d, J = 19.8 Hz, 1H), 2.07 (s, 3H). ^{13}C NMR (176 MHz, acetone-d_6): δ 195.7, 194.7, 162.4, 161.6, 130.0, 125.4, 124.9; 119.5, 114.7, 71.6, 67.2, 50.3, 49.9, 45.8, 23.6; HRMS (m/z): [M + H]$^+$ calcd. for $C_{19}H_{15}O_7$, 355.0812; found, 355.0813; analysis (calcd., found for $C_{19}H_{14}O_7$): C (64.41), H (3.98), O (31.61).

Epoxyfluoxanester B (**26**). ^{1}H NMR (700 MHz, acetone-d_6): δ 7.08 (d, J = 7.8 1H), 7.07 (d, J = 7.8, 1H), 6.98 (t, J = 7.8 Hz, 1H), 6.64 (s, 1H), 6.55 (s, 1H), 4.27 (d, J = 21.5, 1H), 3.86 (d, J = 21.5 Hz, 1H), 3.63 (s, 3H); 2.25 (s, 3H), ^{13}C NMR (176 MHz, acetone-d_6): δ 201.3, 197.3, 166.6, 160.2, 155.2, 143.0; 140.8, 138.2, 124.3, 122.3, 121.8, 120.1, 116.5, 114.4, 113.8, 90.8, 84.2, 52.3; 41.6, 20.3; HRMS (m/z): [M + H]$^+$ calcd. for $C_{20}H_{17}O_8$, 385.0918; found, 385.0923;

analysis (calcd., found for $C_{20}H_{16}O_8$): C (62.50), H (4.20), O (33.30).

Epoxyfluoxanester A (**27**). ^{1}H NMR (700 MHz, acetone-d_6): δ 7.11 (dd, *J* = 7.6, 1.1 1H), 6.99 (dd, *J* = 7.6, 1.1, 1H), 6.95 (t, *J* = 7.6 Hz, 1H), 5.59 (s, 1H), 3.68 (s, 1H), 3.65 (d, *J* = 12.9, 1H), 3.43 (s, 3H), 3.13 (d, *J* = 19.1, 1H), 2.65 (d, *J* = 12.9 Hz, 1H), 2.37 (d, *J* = 19.1 Hz, 1H), 1.99 (s, 3H); ^{13}C NMR (176 MHz, acetone-d_6): δ 199.6, 197.1, 193.8, 167.7, 160.4, 159.7, 143.6, 125.0, 123.1, 123.1, 122.6, 113.8, 87.7, 81.4, 73.4, 55.1, 52.0, 50.5, 42.4, 22.3; HRMS (*m*/*z*): $[M + H]^+$ calcd. for $C_{20}H_{19}O_9$, 403.1024; found, 403.1030; analysis (calcd., found for $C_{20}H_{18}O_9$): C (59.70), H (4.51), O (35.79).

Supplementary Tables

Supplementary Table 1. Strains and plasmids used and constructed in this study.

Strains/Plasmids	Charicteristic(s)	Sources Reverence
E. coli		
DH5α	host strain for cloning	Invitrogen
BL21 (DE3)	host strain for protein expression	Novagen
Actinomycetes		
Micromonospora rosaria SCSIO N160	fluostatin producer	[2]
FLS03	Δ*flsO2* mutant of *M. rosaria* SCSIO N160 producing PJM (**8**)	[1]
Micromonospora echinospora SCSIO 04089	nenestatin producer	[3]
NES004	Δ*nes18* mutant of *M. echinospora* SCSIO 04089 producing **14**	[4]
Streptomyces ambofaciens Δ*alp1U*	template for the amplification of *alpK*	[5]
**Plasmids**		
pET28a	Km^r, expression vector	Novagen
pCSG5102	A 1467 bp *Nde*I/*Bam*HI fragment of *flsO2* [a] by PCR from genomic DNA of *M. rosaria* SCSIO N160 inserted into pET28a	[1]
pCSG5203	A 1494 bp *Nde*I/*Bgl*II fragment of *flsO3* [a] by PCR from genomic DNA of *M. rosaria* SCSIO N160 inserted into pET28a	This study
pCSG5205	A 1494 bp *Nde*I/*Bgl*II fragment of *flsO1* [a] by PCR from genomic DNA of *M. rosaria* SCSIO N160 inserted into pET28a	This study
pCSG5210	A 1479 bp *Nde*I/*Bam*HI fragment of *flsO4* [a] by PCR from genomic DNA of *M. rosaria* SCSIO N160 inserted into pET28a	This study
pCSG5215	A 1491 bp *Nde*I/*Bgl*II fragment of *flsO5* [a] by PCR from genomic DNA of *M. rosaria* SCSIO N160 inserted into pET28a	This study
pCSG5230	A 1476 bp *Nde*I/*Bgl*II fragment of *alpK* [b] by PCR from genomic DNA of *S. ambofaciens*Δ*alp1U* inserted into pET28a	This study
pCSG5231	A 1479 bp *Nde*I/*Bgl*II fragment of *nes26* [c] by PCR from genomic DNA of *M. echinospora* SCSIO 04089 inserted into pET28a	This study
pCSG5232	pCSG5205 derivative carrying *flsO1* R45A mutation	This study
pCSG5233	pCSG5205 derivative carrying *flsO1* H76F mutation	This study
pCSG5234	pCSG5205 derivative carrying *flsO1* R95A mutation	This study
pCSG5235	pCSG5205 derivative carrying *flsO1* M203A mutation	This study
pCSG5236	pCSG5205 derivative carrying *flsO1* L205A mutation	This study
pCSG5237	pCSG5205 derivative carrying *flsO1* R213A mutation	This study
pCSG5238	pCSG5205 derivative carrying *flsO1* V215G mutation	This study
pCSG5239	pCSG5205 derivative carrying *flsO1* F258A mutation	This study
pCSG5240	pCSG5205 derivative carrying *flsO1* M284A mutation	This study
pCSG5241	pCSG5205 derivative carrying *flsO1* Q108G/R109G mutation	This study

[a] The GenBank accession number of *fls* genes (*flsO1*, *flsO2*, *flsO3*, *flsO4* and *flsO5*) is KT726162.1 [https://www.ncbi.nlm.nih.gov/nuccore/KT726162.1].
[b] The GenBank accession number of *alpK* is AY338477.2 [https://www.ncbi.nlm.nih.gov/nuccore/AY338477.2].
[c] The GenBank accession number of *nes26* is KY454837.1 [https://www.ncbi.nlm.nih.gov/nuccore/KY454837.1].

Supplementary Table 2. Primers used in this study.

Primers	Usage targeted	Sequences
**For gene expression**		
FlsO1EF	*flsO1*	5'- GAAGGTCCATATGAACACAATCGATGCG -3' (*Nde*I)
FlsO1ER		5'- GCGGAGATCTTCAGCGGGACGGGCC -3' (*Bgl*II)
FlsO3EF	*flsO3*	5'- ACGCCGGCATATGACCGGCACGCCGCCGCAGAC -3' (*Nde*I)
FlsO3ER		5'- AGGTCAGATCTCTAGTCTTCGGCGGGCGCCG -3' (*Bgl*II)
FlsO4EF	*flsO4*	5'- GAAAGCTCATATGGTGGTCGTGGGTGCC -3' (*Nde*I)
FlsO4ER		5'- CCGCGGATCCTCACGCGGCCACCAC -3' (*Bam*HI)
FlsO5EF	*flsO5*	5'- GAAGGTGCATATGCCCGCCAACGACACG -3' (*Nde*I)
FlsO5ER		5'- GTCGAGATCTTCAGCCCACCGCCGG -3' (*Bgl*II)
AlpKEF	*alpK*	5'- GCAGTGCATATGGAATTCTACGATTCGG -3' (*Nde*I)
AlpKER		5'- CGACAGATCTTCAGGCGGTGGGGCCGAACCAG -3' (*Bgl*II)
Nes26EF	*nes26*	5'- GCAGTGCATATGCCAGAGTTTGACGCG -3' (*Nde*I)
Nes26ER		5'- CGACAGATCTCTAGGCTCGGCCGAA -3' (*Bgl*II)
**For FlsO1 mutagenesis**		
R45AF	FlsO1-R45A	5'- GCCCATGCAGCAGTCG***GCC***GCCCTGGGTTTCTC -3'
R45AR		5'- ***GCC***GACTGCTGCATGGGCTCGGCGAGTCGATCC -3'
H76FF	FlsO1-H76F	5'- CACGATCCCGTTCGGA***TTC***TTCGGCGGCGTGCC -3'
H76FR		5'- ***AAT***CCGAACGGGATCGTGCCGACCTCACCGAAG -3'
R95AF	FlsO1-R95A	5'- CGGCTCGTACGGCGCC***GCC***GGTATCCCGCAGTC -3'
R95AR		5'- ***GCG***GCGCCGTACGAGCCGCCCTTGATCACCCGG -3'
M203AF	FlsO1-M203A	5'- GCCGGGCGGCATGGTG***GCG***GTGCTGCCGCTGGG -3'
M203AR		5'- ***GCC***ACCATGCCGCCCGGCACCAGCTCGCCGGAG -3'
L205AF	FlsO1-L205A	5'- CGGCATGGTGATGGTG***GCG***CCGCTGGGCCCGGT -3'
L205AR		5'- ***GCC***ACCATCACCATGCCGCCCGGCACCAGCTCG -3'
R213AF	FlsO1-R213A	5'- GGGCCCGGTGGCCCAG***GCG***GTGGTCGTCTTCGA -3'
R213AR		5'- ***GCC***TGGGCCACCGGGCCCAGCGGCAGCACCATC -3'
V215GF	FlsO1-V215G	5'- GGTGGCCCAGCGGGTG***GGC***GTCTTCGAGCACGC -3'
V215GR		5'- ***CCC***ACCCGCTGGGCCACCGGGCCCAGCGGCAGC -3'
F258AF	FlsO1-F258A	5'- GCTCTGGGTCAGCTGG***GCC***ACCGACTCCAGCCG -3'
F258AR		5'- ***GCC***CAGCTGACCCAGAGCGGCTTGCCGCCCCGG -3'
M284AF	FlsO1-M284A	5'- CGCCGCGCACATCCAC***GCG***CCGATCGGTGGCCA -3'
M284AR		5'- ***GCG***TGGATGTGCGCGGCGTCGCCGGCGAGCAGG -3'
Q108GF	FlsO1-Q108G/R109G	5'- ATGCTGGCC***GGTGGT***GCGGTCGAGCTCGGCGCC -3'
R109GR		5'- CGC***ACCACC***GGCCAGCATGCCCTCGGT -3'

Supplementary Table 3. ^{13}C NMR data for compounds 16−19, 22, 24 and 25.

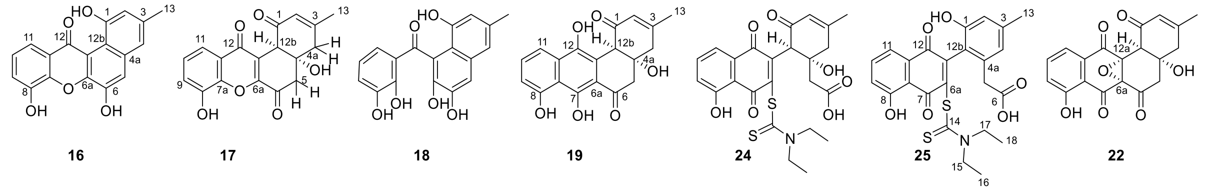

16 17 18 19 24 25 22

position	16[a,3]	17[a,2]	18[a,1]	19[a,1]	24[a,1]	25[a,2]	22[a,b,2]
	*δ*C, type	*δ*C, type	*δ*C, type	*δ*C, type	*δ*C, type	*δ*C, type	*δ*C, type
1	156.1, C	193.7, C	151.7, C	195.6, C	194.0, C	155.7, C	195.7, C
2	116.0, CH	125.6, CH	109.1, CH	125.7, CH	125.0, CH	115.5, CH	125.4, CH
3	138.1, C	159.5, C	133.7, C	160.1, C	160.1, C	140.6, C	162.4, C
4	118.5, CH	45.2, CH_2	116.7, CH	45.8, CH_2	42.9, CH_2	123.2, CH	45.8, CH_2
4a	134.1, C	73.6, C	130.7, C	72.6, C	74.8, C	135.5, C	71.6, C
5	119.7, CH	47.0, CH_2	110.0, CH	45.9, CH_2	41.7, CH_2	39.6, CH_2	49.9, CH_2
6	150.1, C	191.8, C	143.0, C	204.1, C	172.7, C	172.1, C	194.7, C
6a	144.8, C	149.5, C	146.7, C	109.1, C	147.9, C	145.7, C	67.2, C
7				158.3, C	183.7, C	186.3, C	N
7a	143.2, C	144.8, C	150.1, C	113.1, C	116.2, C	117.1, C	114.7, CH
8	146.5, C	147.5, C	146.3, C	157.9, C	159.5, C	161.9, C	161.6, C
9	120.7, CH	125.7, CH	120.9, CH	111.7, CH	123.5, CH	124.3, CH	124.9, CH
10	125.5, CH	115.1, CH	118.0, CH	132.1, CH	136.5, CH	137.5, CH	N
11	116.1, CH	120.4, CH	122.9, CH	113.7, CH	118.9, CH	120.0, CH	119.5, CH
11a	122.7, C	124.1, C	123.0, C	133.3, C	132.3, C	133.7, C	130.0, C
12	180.8, C	178.1, C	204.7, C	143.8, C	181.8, C	182.2, C	N
12a	116.5, C	125.1, C	118.5, C	115.3, C	148.7, C	150.4, C	N
12b	113.2, C	51.6, CH	115.6, C	53.6, CH	62.8, CH	119.8, C	50.3, CH
13	20.2 CH_3	23.0, CH_3	21.6, CH_3	24.2, CH_3	24.3, CH_3	21.4, CH_3	23.6, CH_3
14					190.7, C	192.2, C	
15					48.6, CH_2	49.8, CH_2	
16					11.4, CH_3	13.1, CH_3	
17					48.7, CH_2	49.5, CH_2	
18					12.9, CH_3	11.6, CH_3	

[a] ^{1}H and ^{13}C NMR were recorded at 700 and 176 MHz, respectively.
[b] ^{13}C NMR Data was assigned by HSQC and HMBC.
[1] Measured in DMSO-*d*6,
[2] Measured in acetone-*d*6,
[3] Measured in acetone-*d*6 /DMSO-*d*6 (20:1)
N: no signal was observed.
Assignments were based on DEPT, HSQC, COSY, HMBC, and NOESY experiments.

Supplementary Table 4. ^{1}H NMR data for compounds 16−19, 22, 24 and 25.

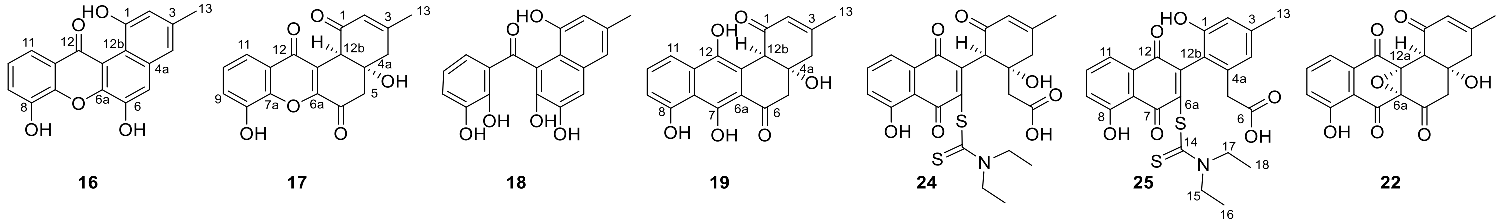

No.	**16**[a,3] δ_H, multi (J in Hz)	**17**[a,2] δ_H, multi (J in Hz)	**18**[a,1] δ_H, multi (J in Hz)	**19**[a,1] δ_H, multi (J in Hz)	No.	**24**[a,1] δ_H, multi (J in Hz)	**25**[a,2] δ_H, multi (J in Hz)	**22**[a,2] δ_H, multi (J in Hz)
2	6.84, d (1.5)	6.01, s	6.30, s	5.99, s		5.98, s	6.67, s	5.92, s
4	7.12, d (1.5)	3.09, d (19.2)<br>2.70, d (19.2)	6.89, s	2.87, d (18.9)<br>2.53, d (18.9)		2.87, d (16.6)<br>2.68, d (16.6)	6.79, s	2.94, d (19.8)<br>2.63, d (19.8)
5	7.64, s	3.08, d (17.8)<br>2.73, dd (17.8, 2.2)	7.07, s	2.91, d (17.6)<br>2.52, dd (17.6, 2.4)		2.26, d (14.6)<br>2.56, d (14.6)	3.45, d (15.6)<br>3.63, d (15.6)	2.96, d (17.0)<br>2.79, d (17.0)
9	7.41, dd (7.9, 1.6)	7.35, d (4.8)	6.95, dd (7.1, 2.2)	6.88, dd (7.6, 1.1)		7.37, d (8.7)	7.36, d (8.0)	7.43, d (7.3)
10	7.43, t (7.9)	7.59, dd (4.8, 4.8)	6.55, dd (8.1, 7.1)	7.58, dd (8.4, 7.6)		7.77, dd (8.7, 7.0)	7.79, t (8.0)	7.88, dd (8.1, 7.3)
11	7.85, dd(7.9, 1.6)	7.35, d (4.8)	6.54, dd (8.1, 2.2)	7.62, dd (8.4, 1.1)		7.51, d (7.0)	7.64, d (8.0)	7.74, d (8.1)
12b		4.35, d (2.2)		4.35, d (2.4)		4.10, s		4.26, d (1.1)
13	2.40, s	2.08, s	2.26, s	2.02, s		2.02, s	2.29, s	2.07, s
1-OH	12.90, s							
4a-OH				5.63, s	15	3.85, m	3.85, m	
7-OH				15.37, s	16	1.18, t (7.1)	1.34, t (7.1)	
8-OH				9.88, s	17	3.93, m	3.92, m, 3.97, m	
12-OH				8.68, s	18	1.42, t (6.9)	1.23, t (7.1)	

[a] ^{1}H and ^{13}C NMR were recorded at 700 and 176 MHz, respectively.
[1]Measured in DMSO-d_6,
[2] Measured in acetone-d_6,
[3] Measured in acetone-d_6/DMSO-d_6 (20:1).
Assignments were based on DEPT, HSQC, COSY, HMBC, and NOESY experiments.

Supplementary Table 5. ^{1}H NMR (700 MHz) data and ^{13}C (176 MHz) NMR data for compounds 26 and 27 in acetone-d_6 .

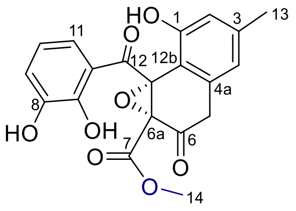

26

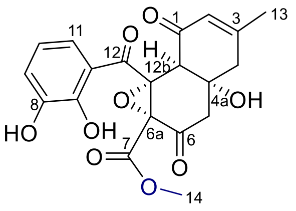

27

Position	**26**		**27**	
	δ_H, mult. (J in Hz)	δ_C, type	δ_H, mult. (J in Hz)	δ_C, type
1		155.2, C		193.8, C
2	6.55, s	114.4, CH	5.59, s	125.0, CH
3		140.8, C		160.4, C
4	6.64, s	120.1, CH	2.37, d (19.1)	42.4, CH_2
			3.13, d (19.1)	
4a		138.2, C		73.4, C
5	3.86, d (21.5)	41.6, CH_2	2.65, d (12.9)	50.5, CH_2
	4.27, d (21.5)		3.65, d (12.9)	
6		201.3, C		199.6, C
6a		84.2, C		81.4, C
7		166.6, C		167.7, C
7a		160.2, C		159.7, C
8		143.0, C		143.6, C
9	7.08, d (7.8)	122.3, CH	7.11, dd (7.6, 1.1)	123.1, CH
10	6.98, t (7.8)	121.8, CH	6.95, t (7.6)	123.1, CH
11	7.07, d (7.8)	113.8, CH	6.99, dd (7.6, 1.1)	113.8, CH
11a		124.3, C		122.6, C
12		197.3, C		197.1, C
12a		90.8, C		87.7, C
12b		116.5, C	3.68, s	55.1, CH
13	2.25, s	20.3, CH_3	1.99, s	22.3, CH_3
14	3.63, s	52.3, CH_3	3.43, s	52.0, CH_3

Assignments were based on DEPT, HSQC, COSY, and HMBC experiments.

Supplementary Table 6. Data collection of FlsO1/FAD and refinement statistics.

Protein Data Bank accession code	7VWP
Wavelength (Å)	1.5418
Resolution range	13.18 - 2.3 (2.382 - 2.3)
Space group	P 1
Unit cell	76.5405 84.6364 102.799 91.3136 107.86 115.391
Total reflections	345820 (30705)
Unique reflections	95966 (9481)
Multiplicity	3.6 (3.2)
Completeness (%)	98.51 (97.86)
Mean I/sigma(I)	12.93 (2.78)
Wilson B-factor	24.80
R-merge	0.07412 (0.3781)
R-meas	0.08706 (0.4532)
R-pim	0.04506 (0.2461)
CC1/2	0.997 (0.913)
CC*	0.999 (0.977)
Reflections used in refinement	95553 (9465)
Reflections used for R-free	4704 (470)
R-work	0.1959 (0.2477)
R-free	0.2600 (0.3384)
CC(work)	0.958 (0.892)
CC(free)	0.921 (0.733)
Number of non-hydrogen atoms	15777
macromolecules	14445
ligands	406
solvent	1047
Protein residues	1926
RMS(bonds)	0.014
RMS(angles)	1.47
Ramachandran favored (%)	95.97
Ramachandran allowed (%)	3.92
Ramachandran outliers (%)	0.11
Rotamer outliers (%)	2.02
Clashscore	9.62
Average B-factor	35.75
macromolecules	35.91
ligands	35.04
solvent	33.70

Statistics for the highest-resolution shell are shown in parentheses.

Supplementary Figures

Condensation of phloroglucinol carboxylic acids with orcinol derivatives

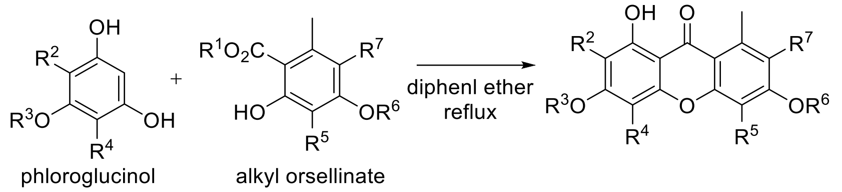

R^1 = Me, Et; R^2 = R^3 = H, Me; R^4 = R^5 = R^7 = H, Cl; R^6 = H, Me, Bn

Nucleophilic addition of diphenol

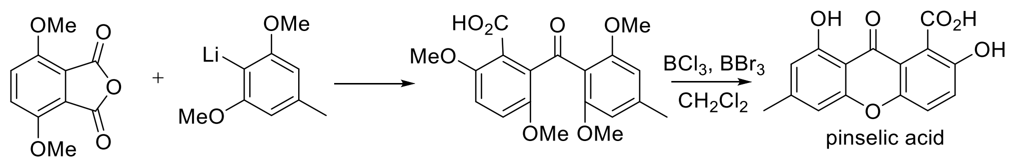

A variant of the Ullmann reaction mediated with acid chloride intermediate

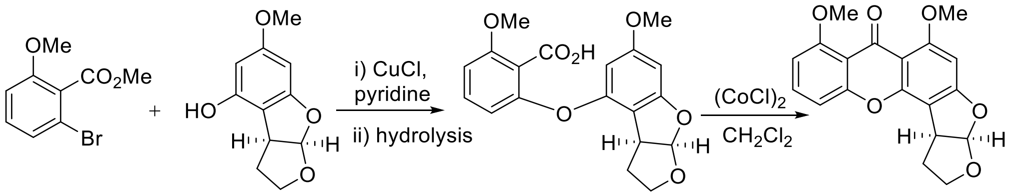

Supplementary Fig. 1. Representative methods for the chemical synthesis of the xanthone skeleton. The representative synthetic methods were reported previously[6].

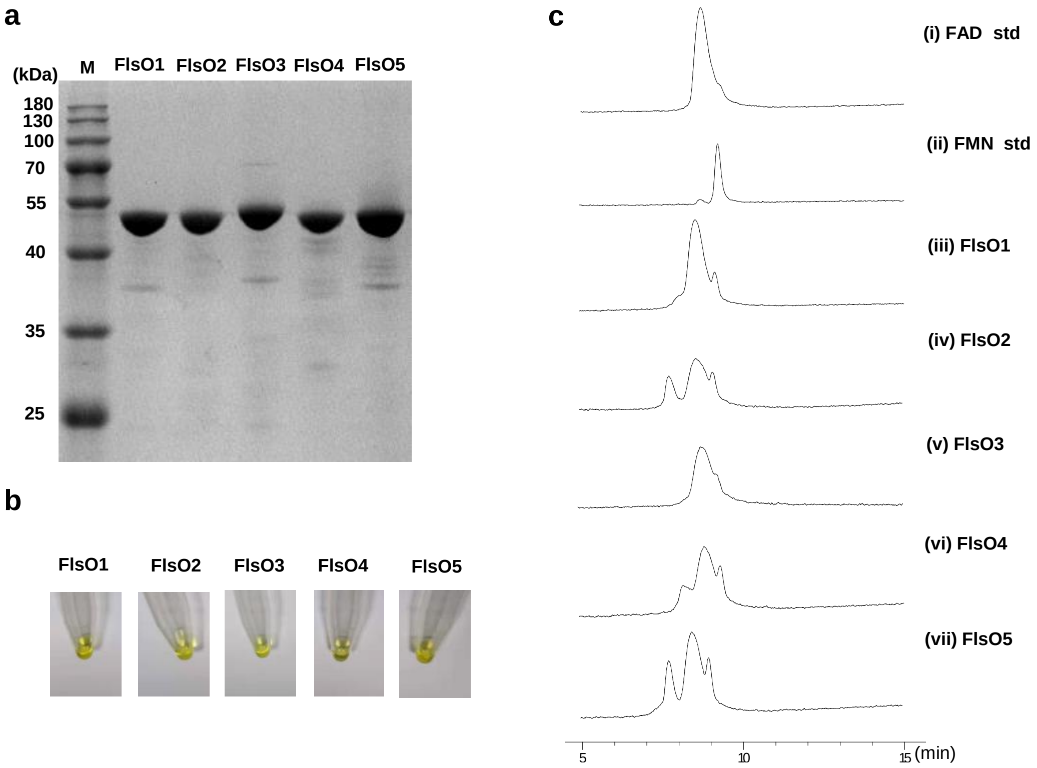

Supplementary Fig. 2. SDS-PAGE analysis of FlsO1 and its homologous proteins and determination of the associated cofactors. (**a**) SDS-PAGE analysis of purified FlsO1, FlsO2, FlsO3, FlsO4, and FlsO5. These experiments were repeated in tripliate. (**b**) Yellow-colored supernatants of FlsO1, FlsO2, FlsO3, FlsO4, and FlsO5. (**c**) HPLC analysis of supernatants of FlsO1, FlsO2, FlsO3, FlsO4, and FlsO5, which were treated in boiling water for 10 min and centrifuged at 14000 rpm for 10 min to determine the associated cofactors. (i) FAD and (ii) FMN as standards; (iii) FlsO1; (iv) FlsO2; (v) FlsO3; (vi) FlsO4; and (vii) FlsO5.

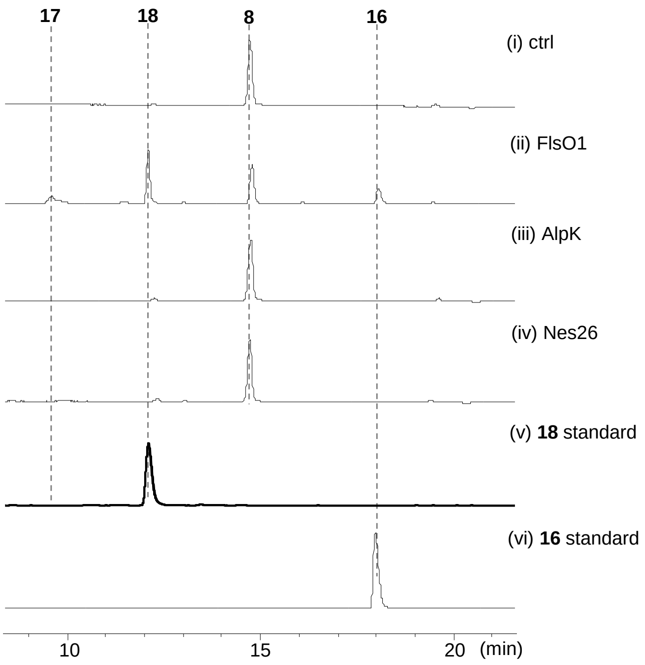

Supplementary Fig. 3. HPLC analysis of enzyme reactions of FlsO1, AlpK, and Nes26 with PJM (8). The assays were performed by incubation of 200 µM PJM (**8**) in the presence of 2 mM NADPH, with (i) control (no enzyme); (ii) 10 µM FlsO1; (iii) 10 µM AlpK; (iv) 10 µM Nes26; (v) **18** standard; (vi) **16** standard. The reactions were performed in 50 mM PBS buffers (pH 7.0) at 30 °C for 30 min.

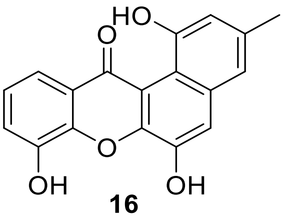

16

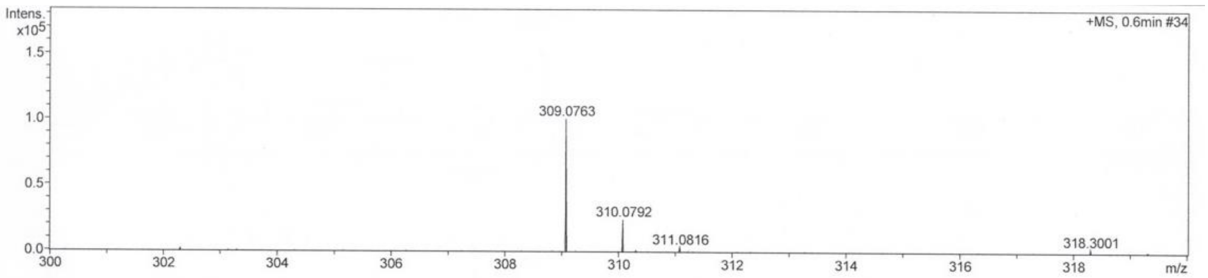

Meas. m/z	#	Ion Formula	Score	m/z	err [ppm]	err [mDa]	mSigma	rdb	e⁻ Conf	N-Rule
309.0763	1	C18H13O5	100.00	309.0757	1.8	0.6	26.4	12.5	even	ok
617.1447	1	C36H25O10	100.00	617.1442	-0.8	-0.5	12.0	24.5	even	ok

Supplementary Fig. 4. HRESIMS spectrum of fluoxanthone A (16).

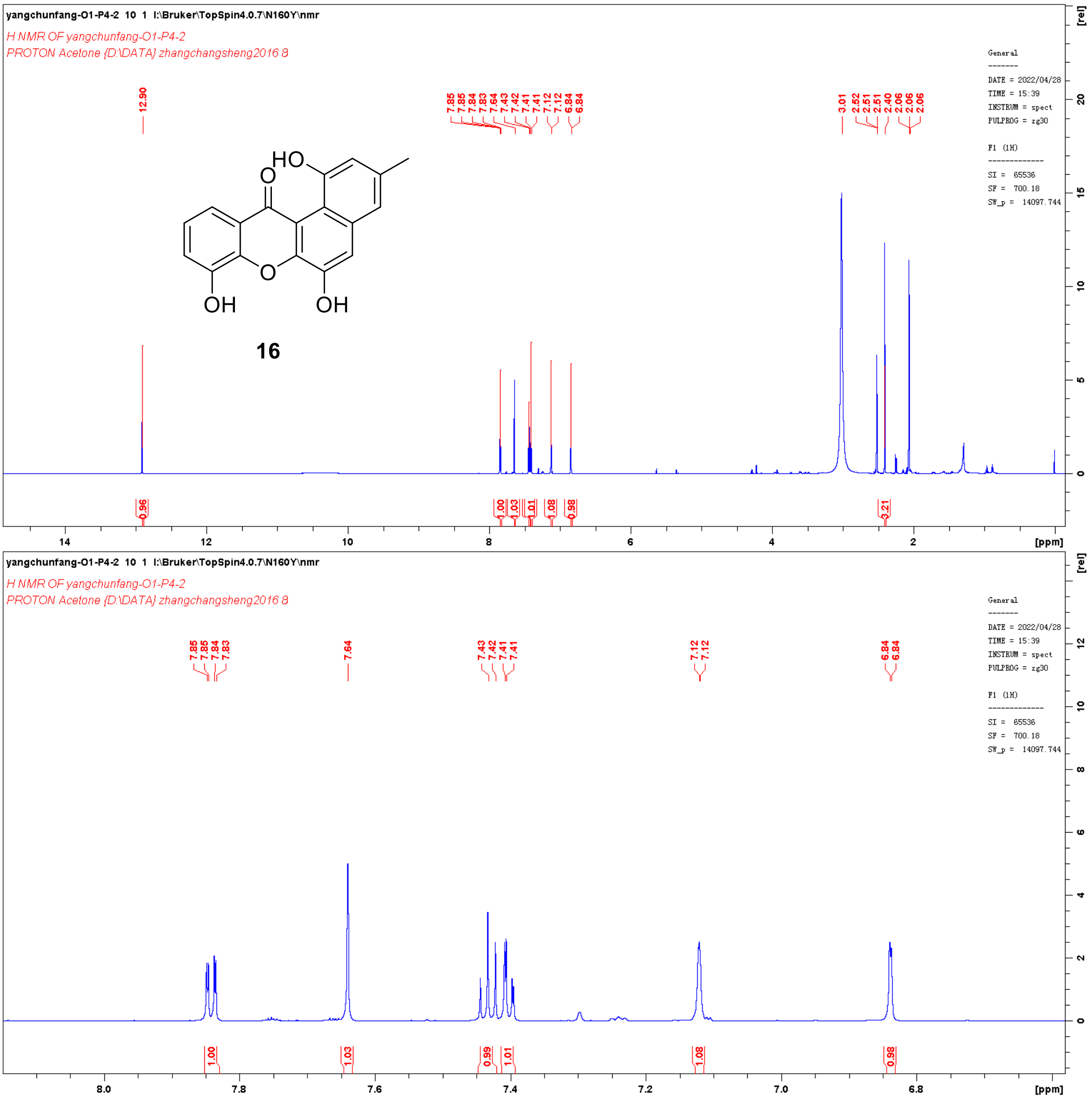

Supplementary Fig. 5. The ^{1}H NMR (700 MHz) spectrum of fluoxanthone A (16) and its partial ^{1}H NMR from ppm 6.8 to ppm 8.0 in acetone-d$_6$/DMSO-d_6 (20:1).

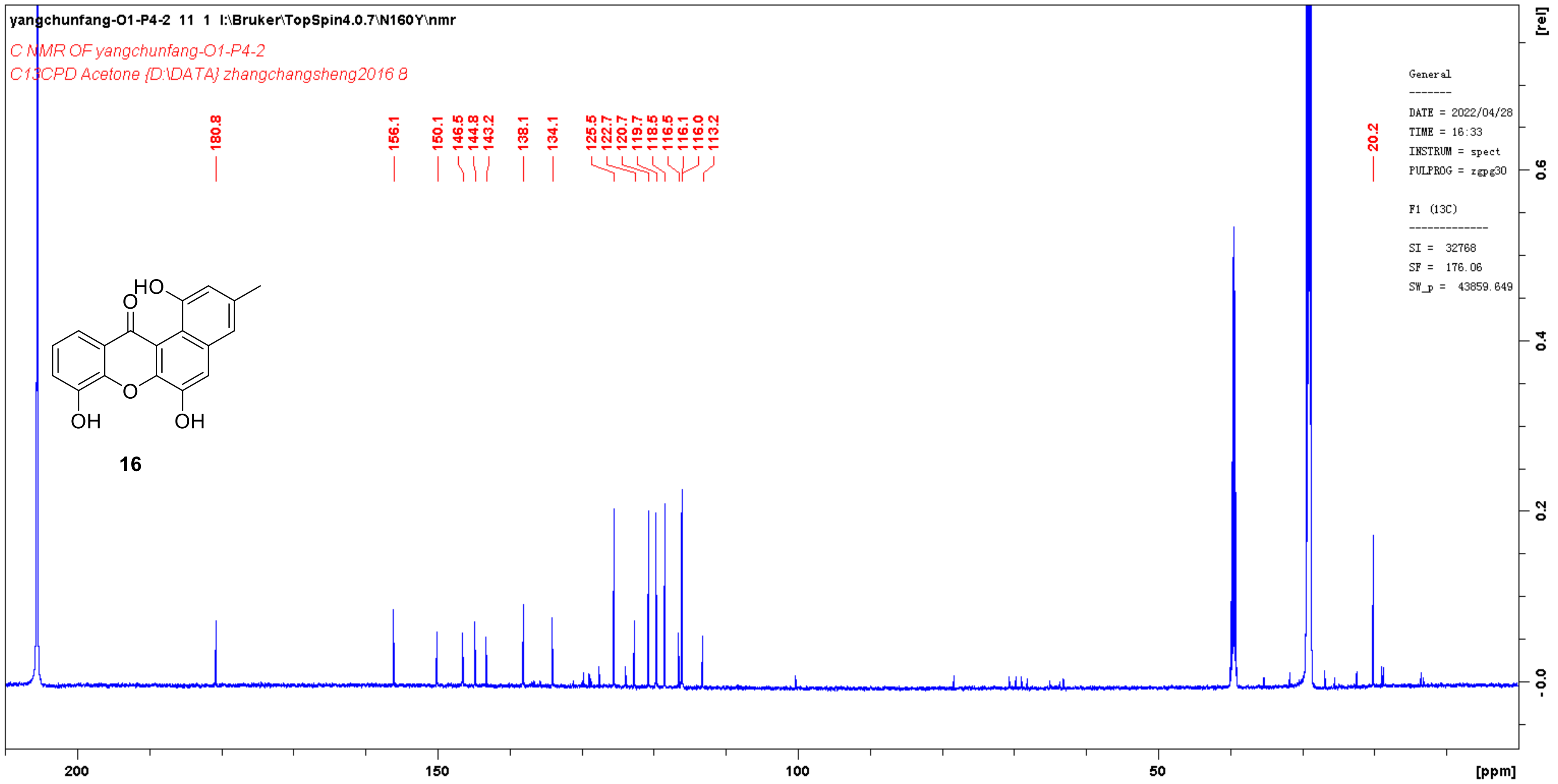

Supplementary Fig. 6. The ^{13}C NMR (176 MHz) spectrum of fluoxanthone A (16) in acetone-d_6/DMSO-d_6 (20:1).

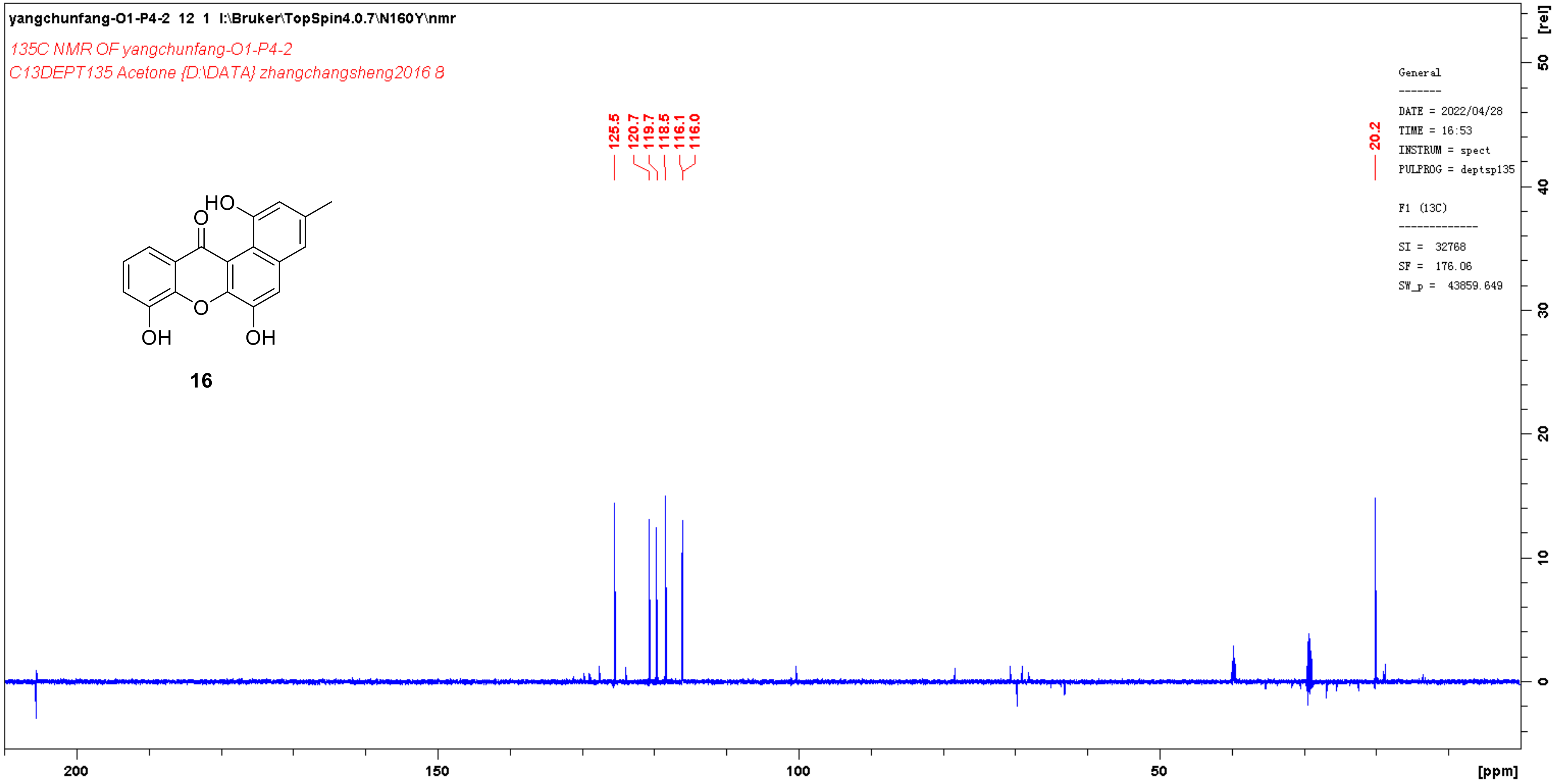

Supplementary Fig. 7. The DEPT 135 spectrum of fluoxanthone A (16) in acetone-d_6/DMSO-d_6 (20:1).

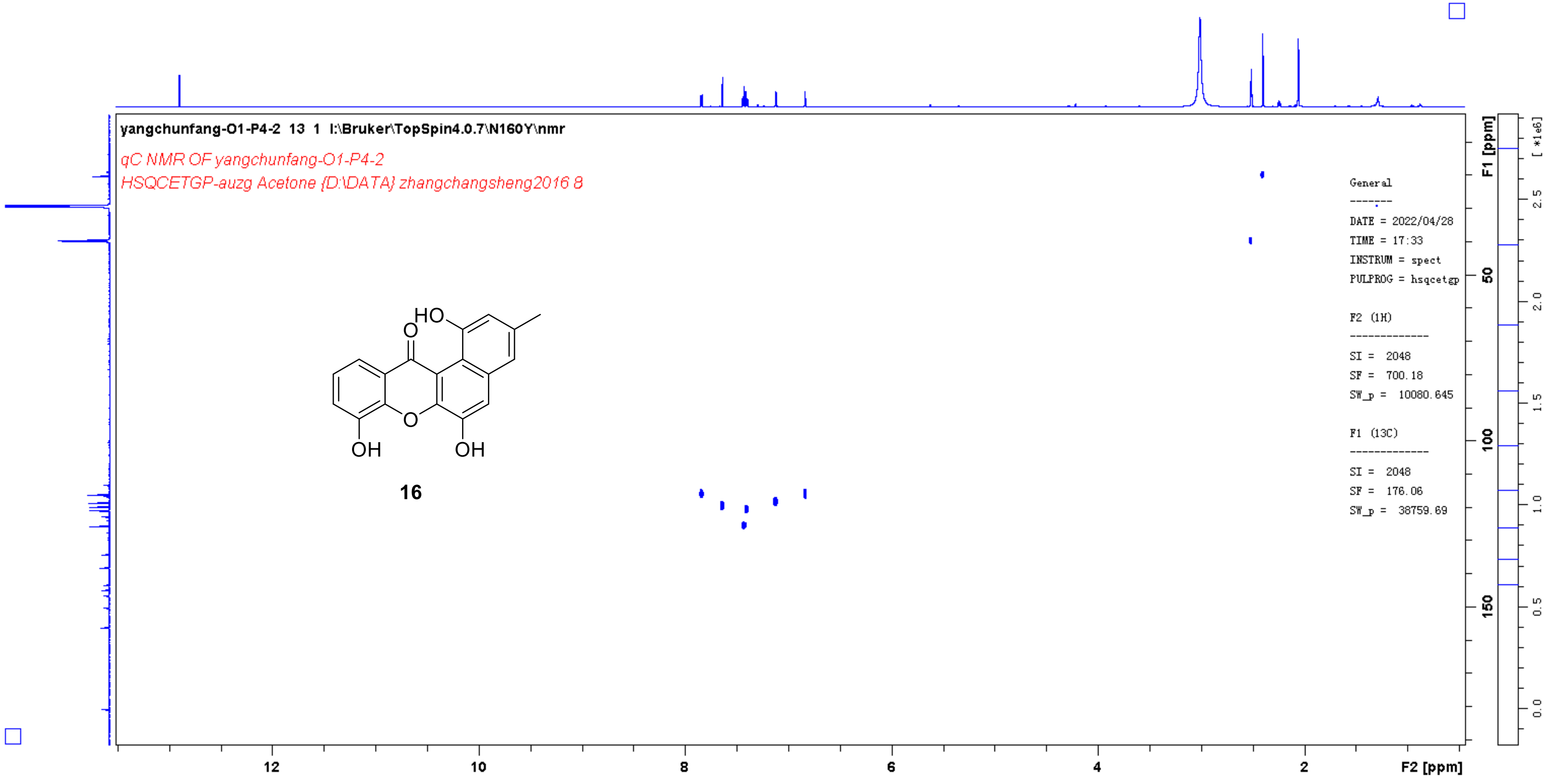

Supplementary Fig. 8. The HSQC spectrum of fluoxanthone A (16) in acetone-d_6/DMSO-d_6 (20:1).

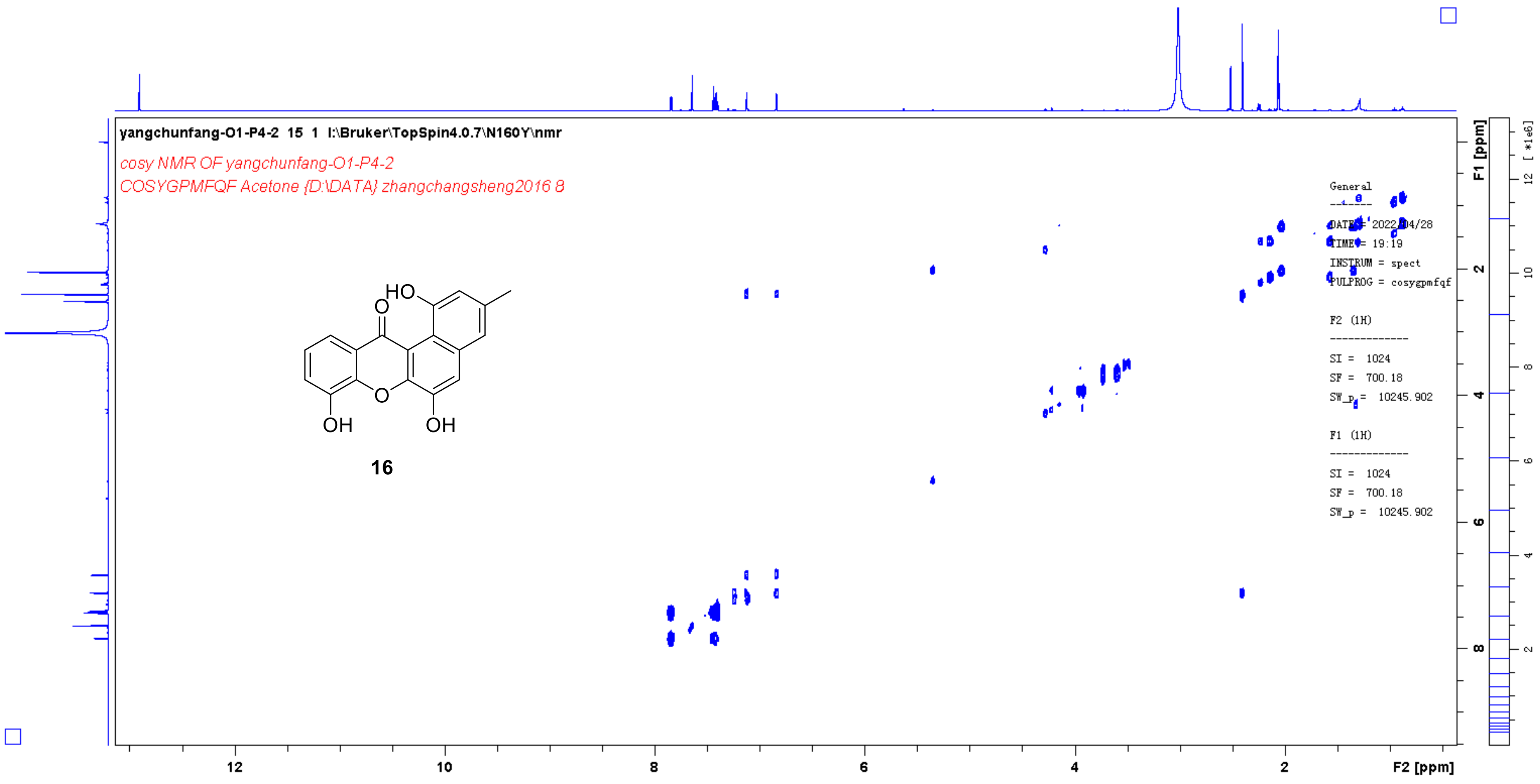

Supplementary Fig. 9. The COSY spectrum of fluoxanthone A (16) in acetone-d_6/DMSO-d_6 (20:1).

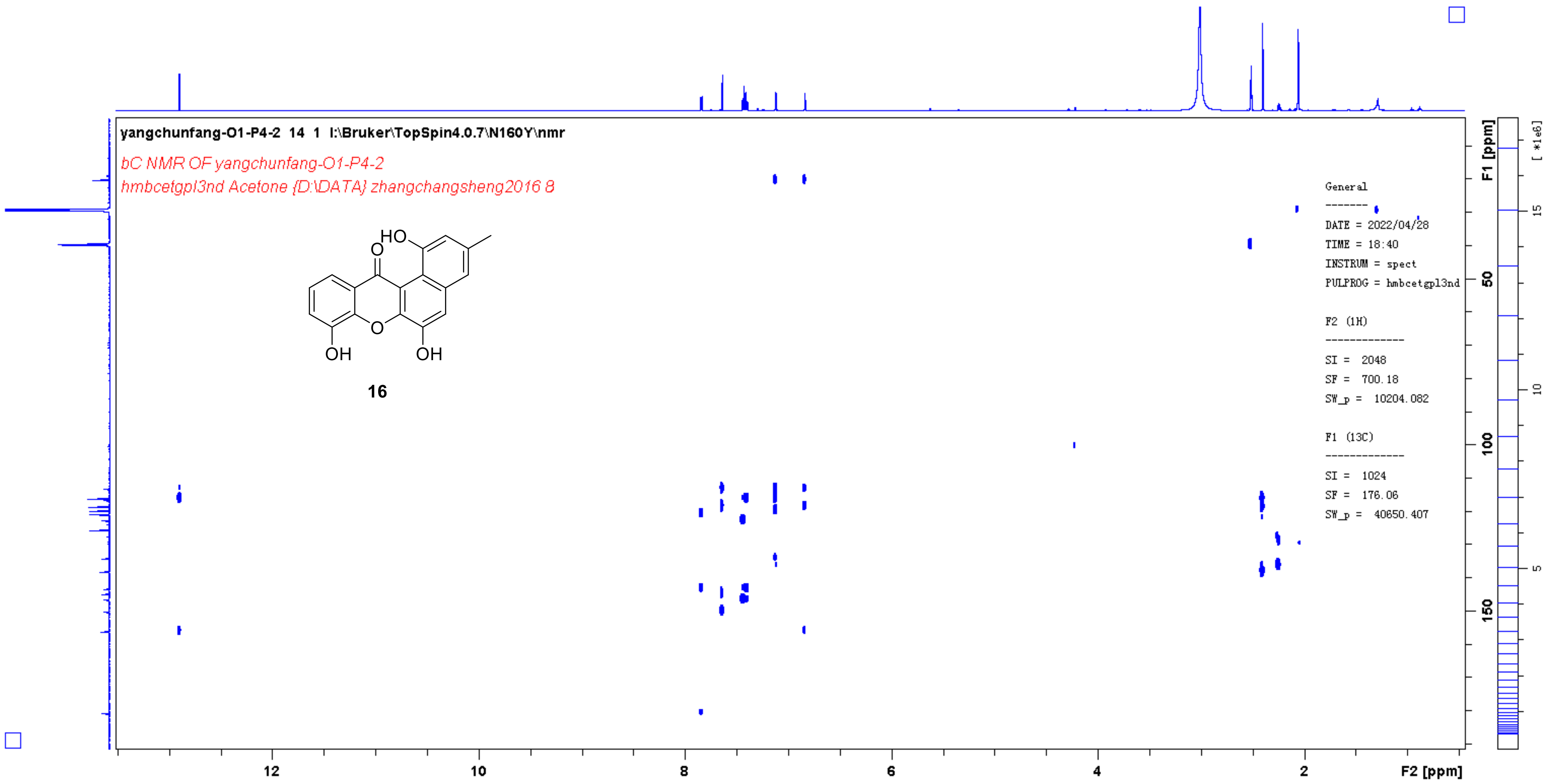

Supplementary Fig. 10. The HMBC spectrum of fluoxanthone A (16) in acetone-d_6/DMSO-d_6 (20:1).

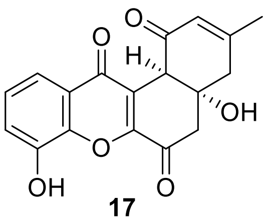

17

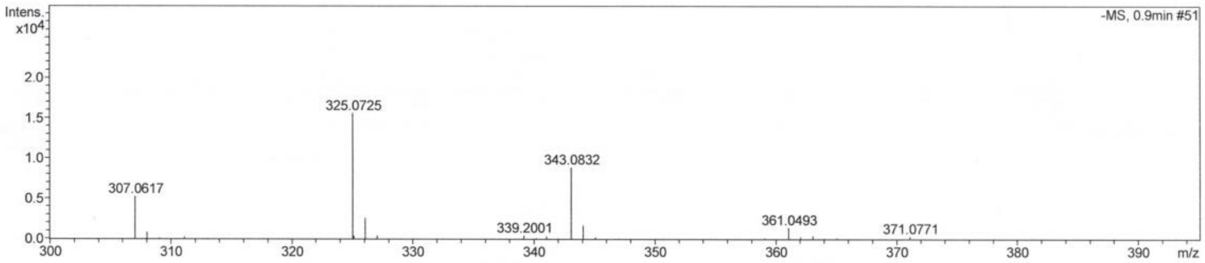

Meas. m/z	#	Ion Formula	Score	m/z	err [ppm]	err [mDa]	mSigma	rdb	e⁻ Conf	N-Rule
325.0725	1	C18H13O6	100.00	325.0718	2.1	0.7	14.8	12.5	even	ok
361.0493	1	C18H14ClO6	100.00	361.0484	2.3	0.8	24.2	11.5	even	ok
651.1528	1	C36H27O12	100.00	651.1508	-3.0	-2.0	79.8	23.5	even	ok
687.1336	1	C36H28ClO12	100.00	687.1275	-8.9	-6.1	137.9	22.5	even	ok

Supplementary Fig. 11. HRESIMS spectrum of fluoxanthone B (17).

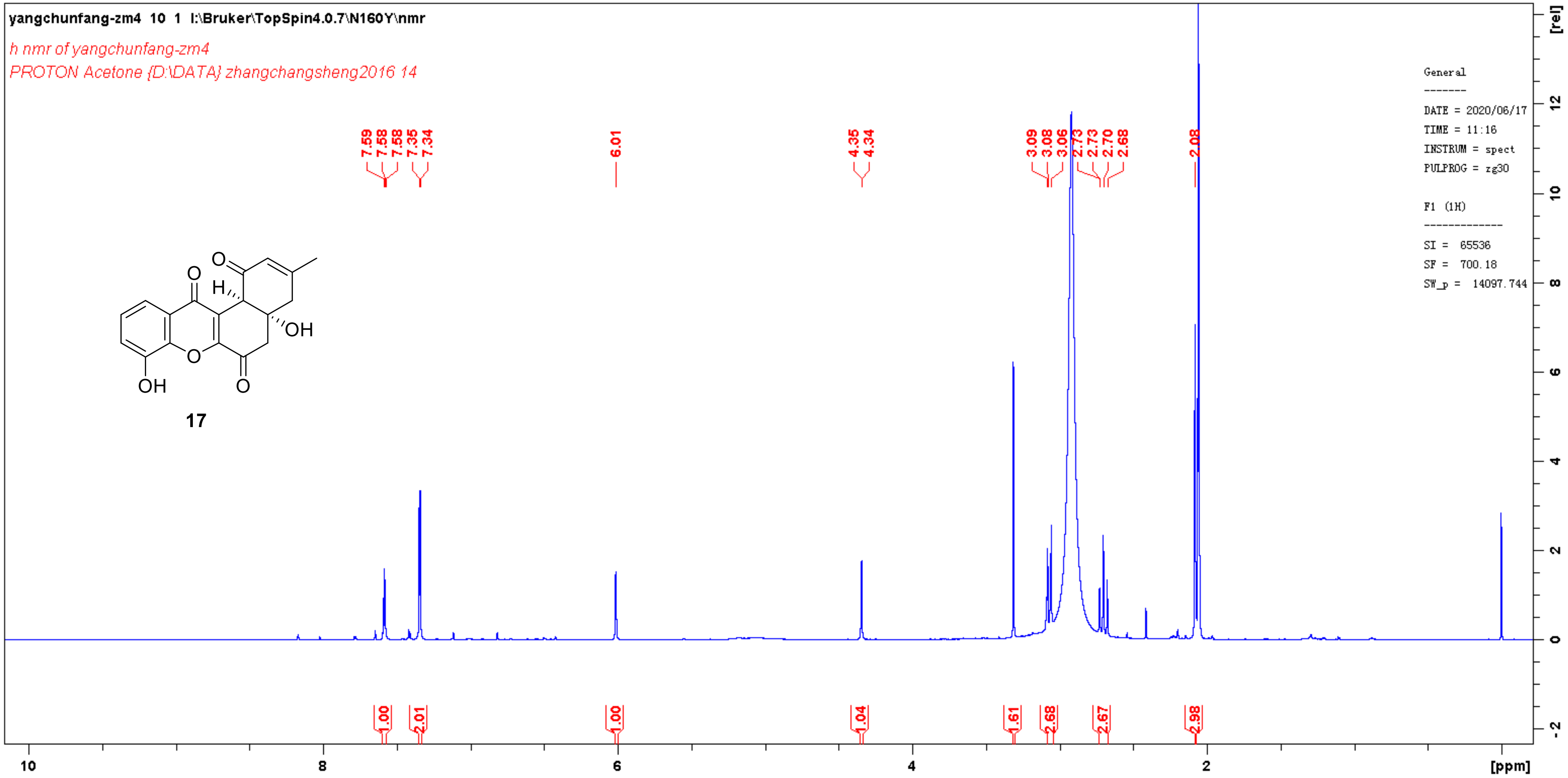

Supplementary Fig. 12. The ^{1}H NMR (700 MHz) spectrum of fluoxanthone B (17) in acetone-d_6.

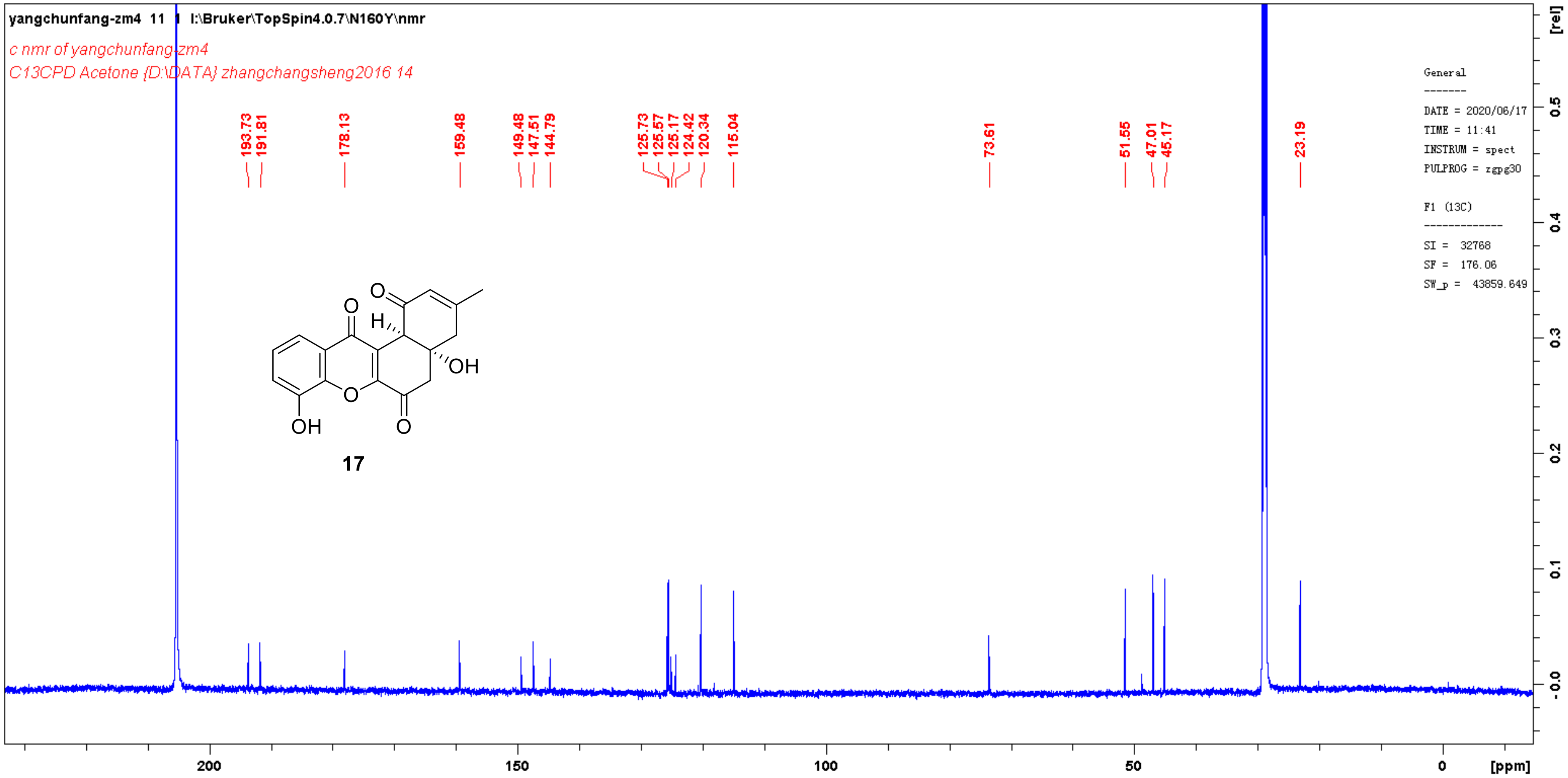

Supplementary Fig. 13. The ^{13}C (176 MHz) spectrum of fluoxanthone B (17) in acetone-d_6.

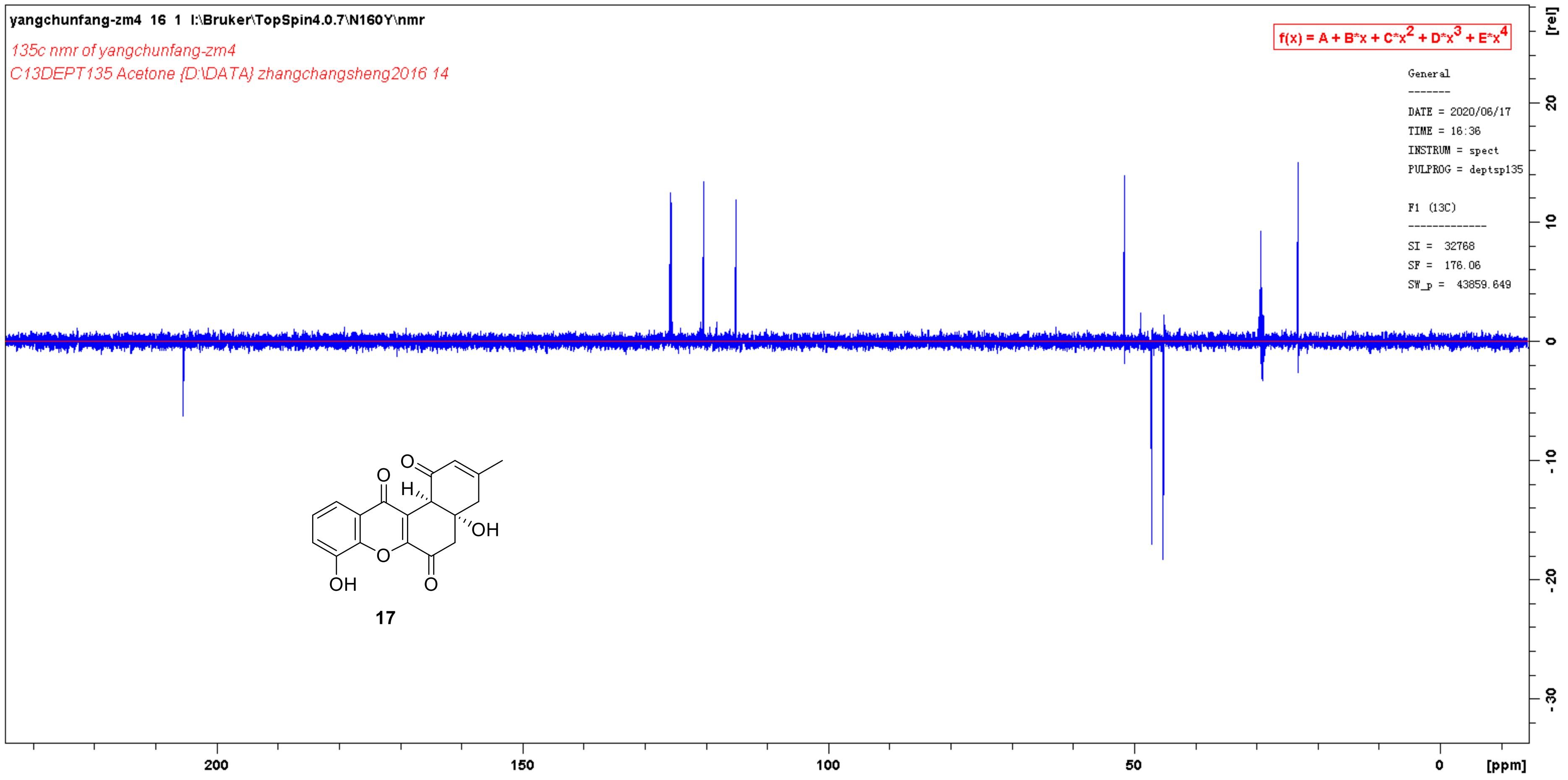

Supplementary Fig. 14. The DEPT 135 spectrum of fluoxanthone B (17) in acetone-d_6.

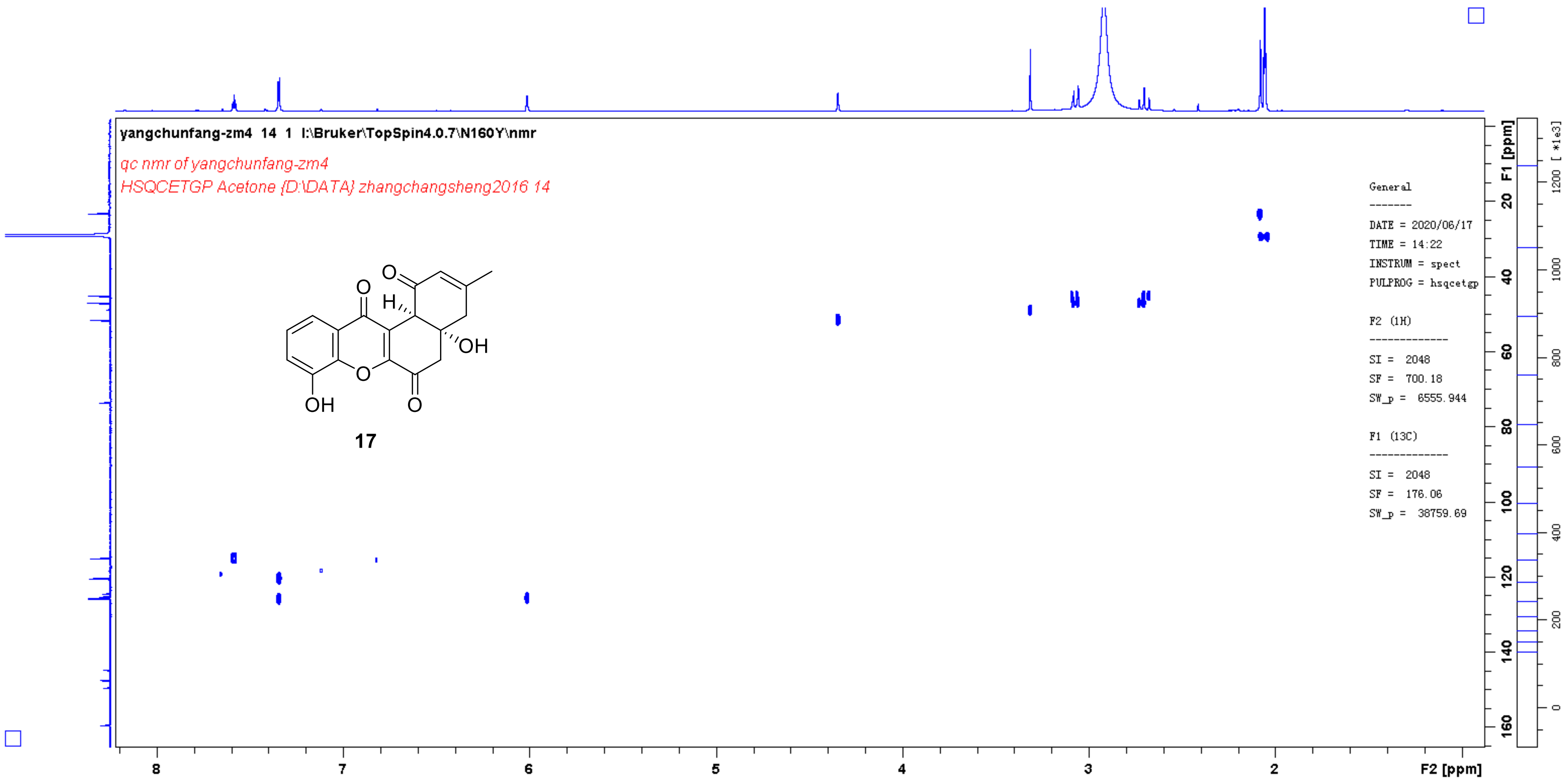

Supplementary Fig. 15. The HSQC spectrum of fluoxanthone B (17) in acetone-d_6.

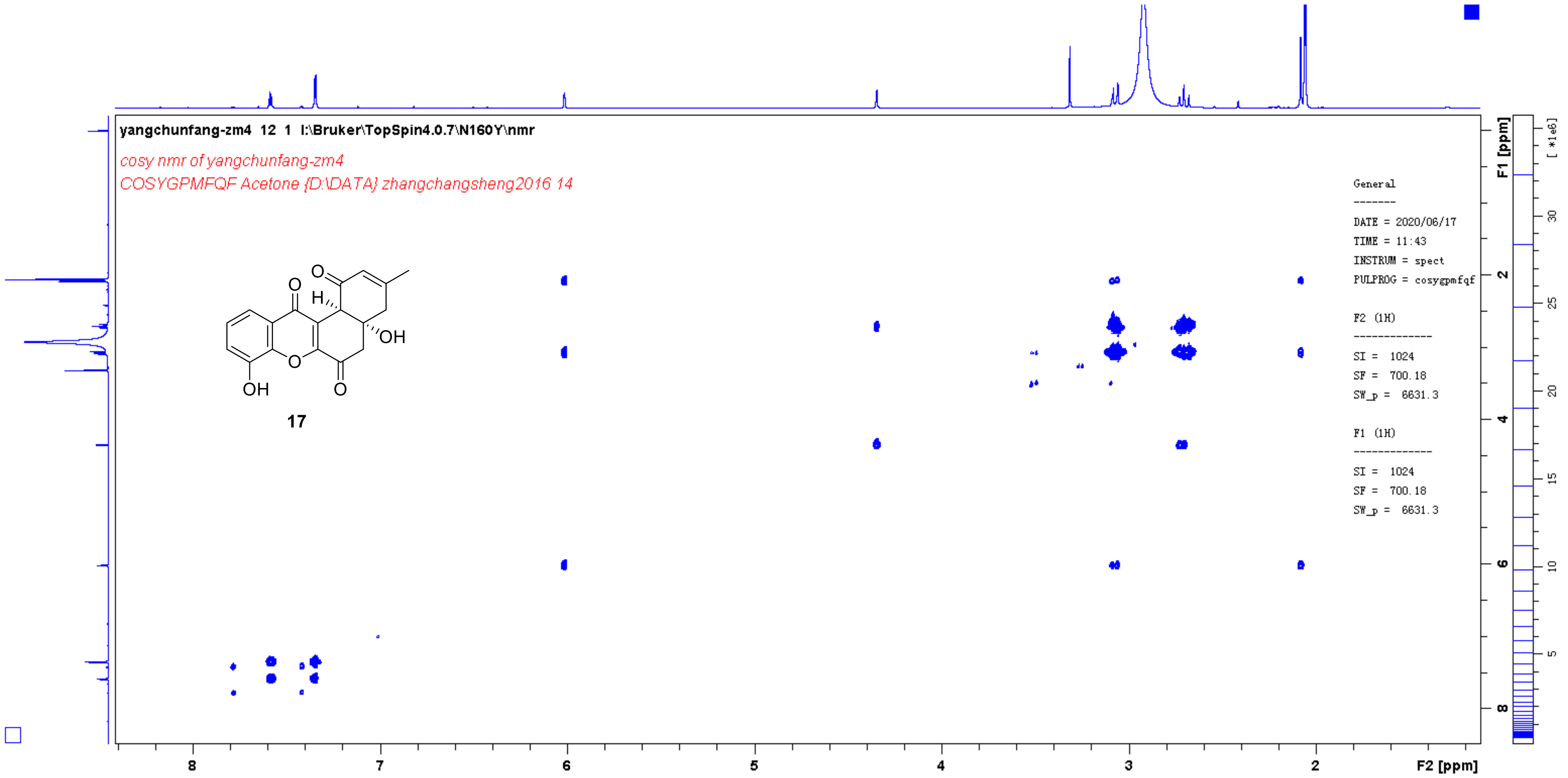

Supplementary Fig. 16. The COSY spectrum of fluoxanthone B (17) in acetone-d_6.

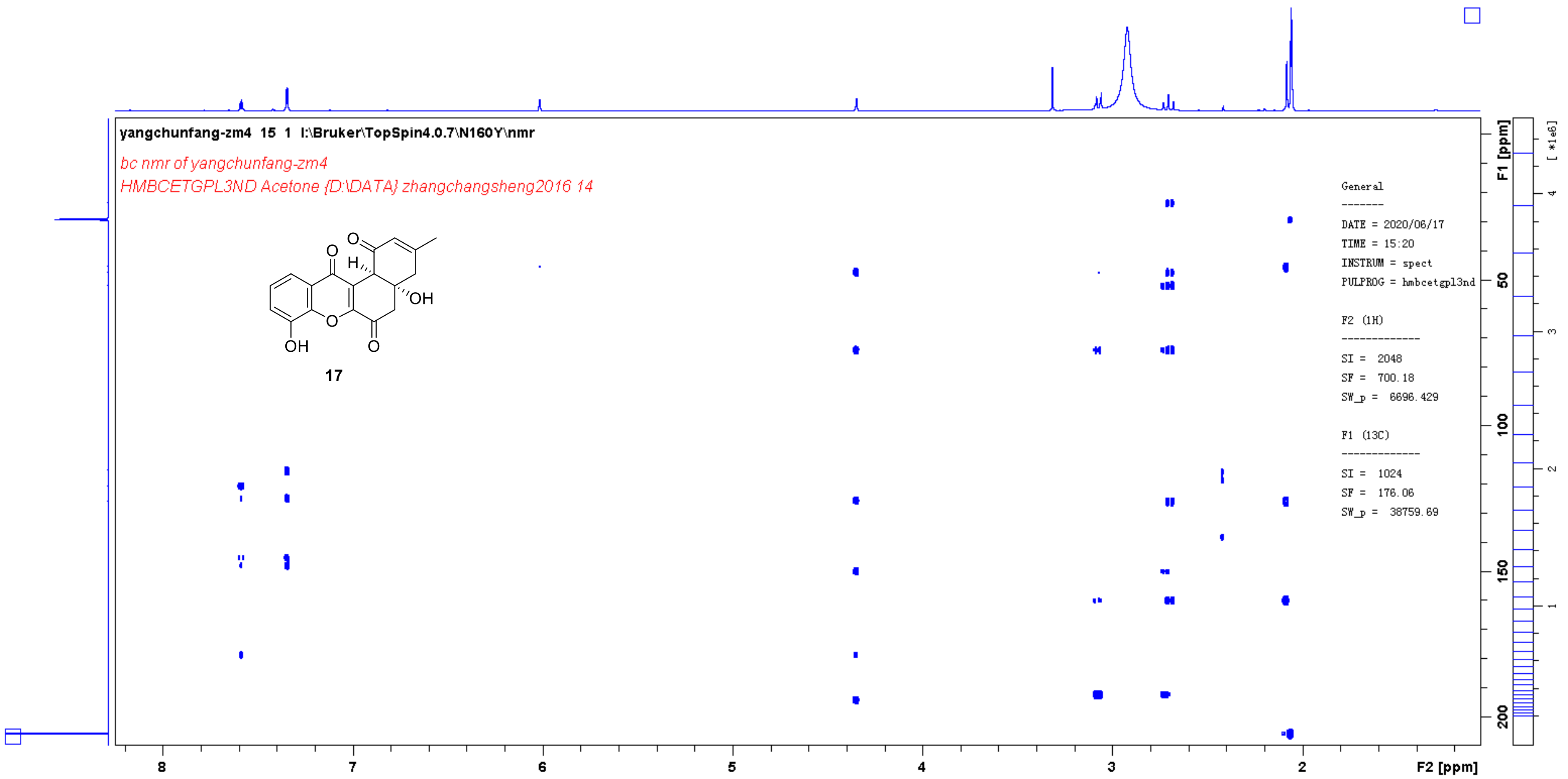

Supplementary Fig. 17. The HMBC spectrum of fluoxanthone B (17) in acetone-d_6.

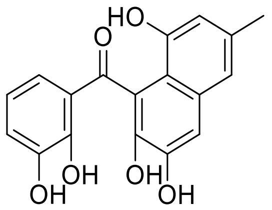

18

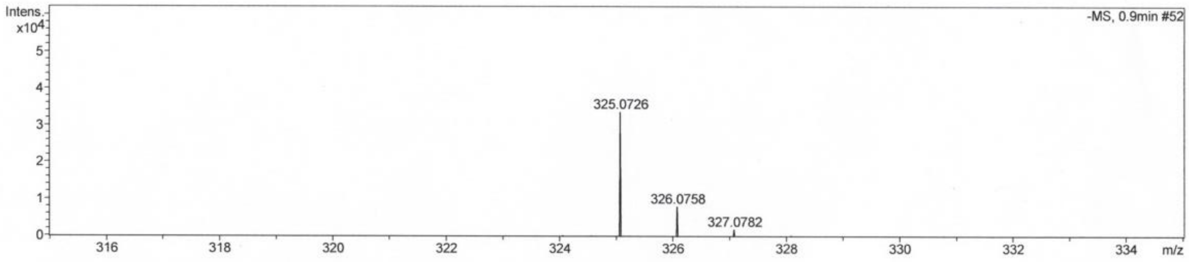

Meas. m/z	#	Ion Formula	Score	m/z	err [ppm]	err [mDa]	mSigma	rdb	e⁻ Conf	N-Rule
325.0726	1	C18H13O6	100.00	325.0718	2.6	0.9	23.2	12.5	even	ok

Supplementary Fig. 18. HRESIMS spectrum of fluoxanol (18).

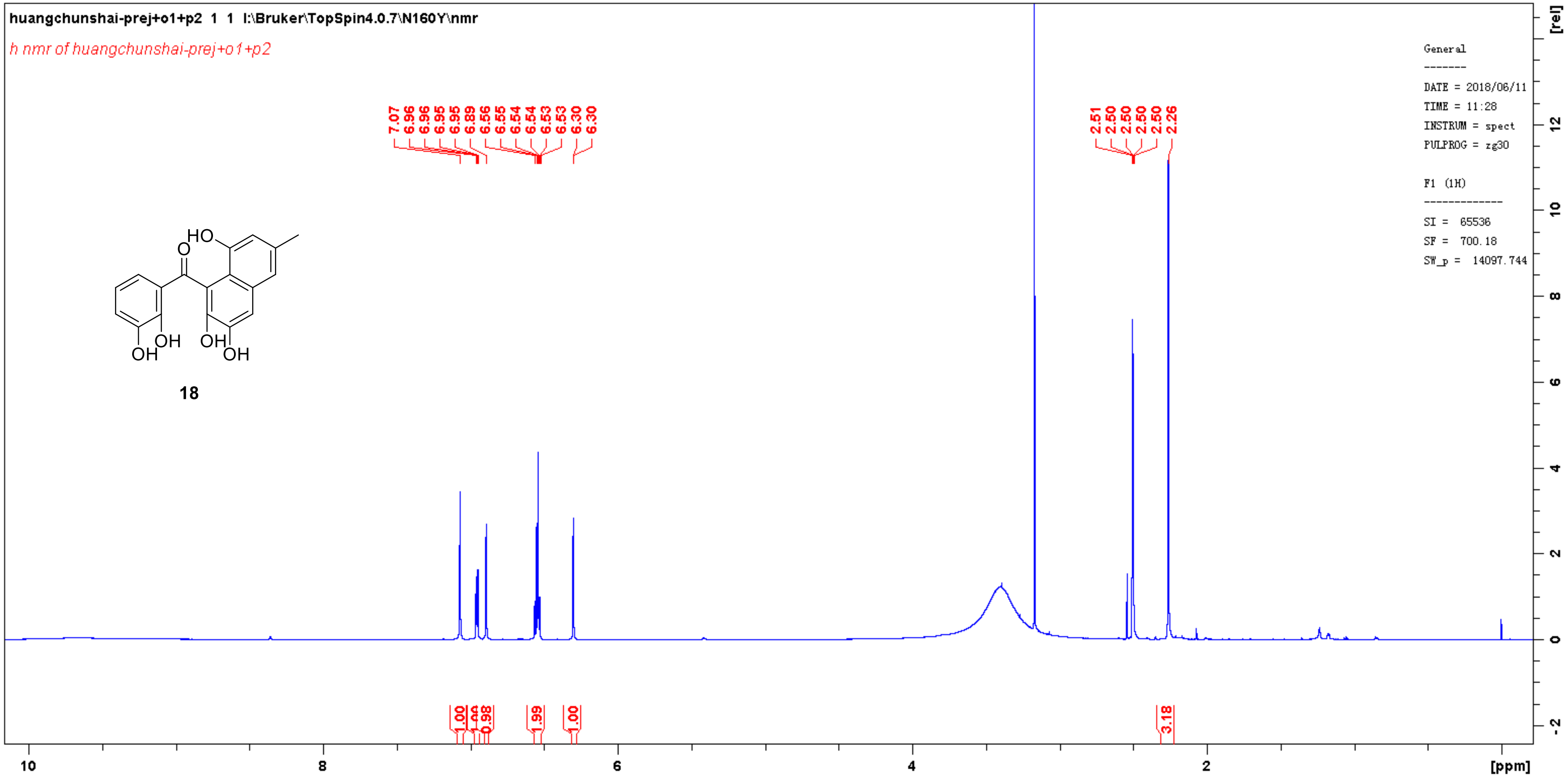

Supplementary Fig. 19. The ^{1}H NMR (700 MHz) spectrum of fluoxanol (18) in DMSO-d_6.

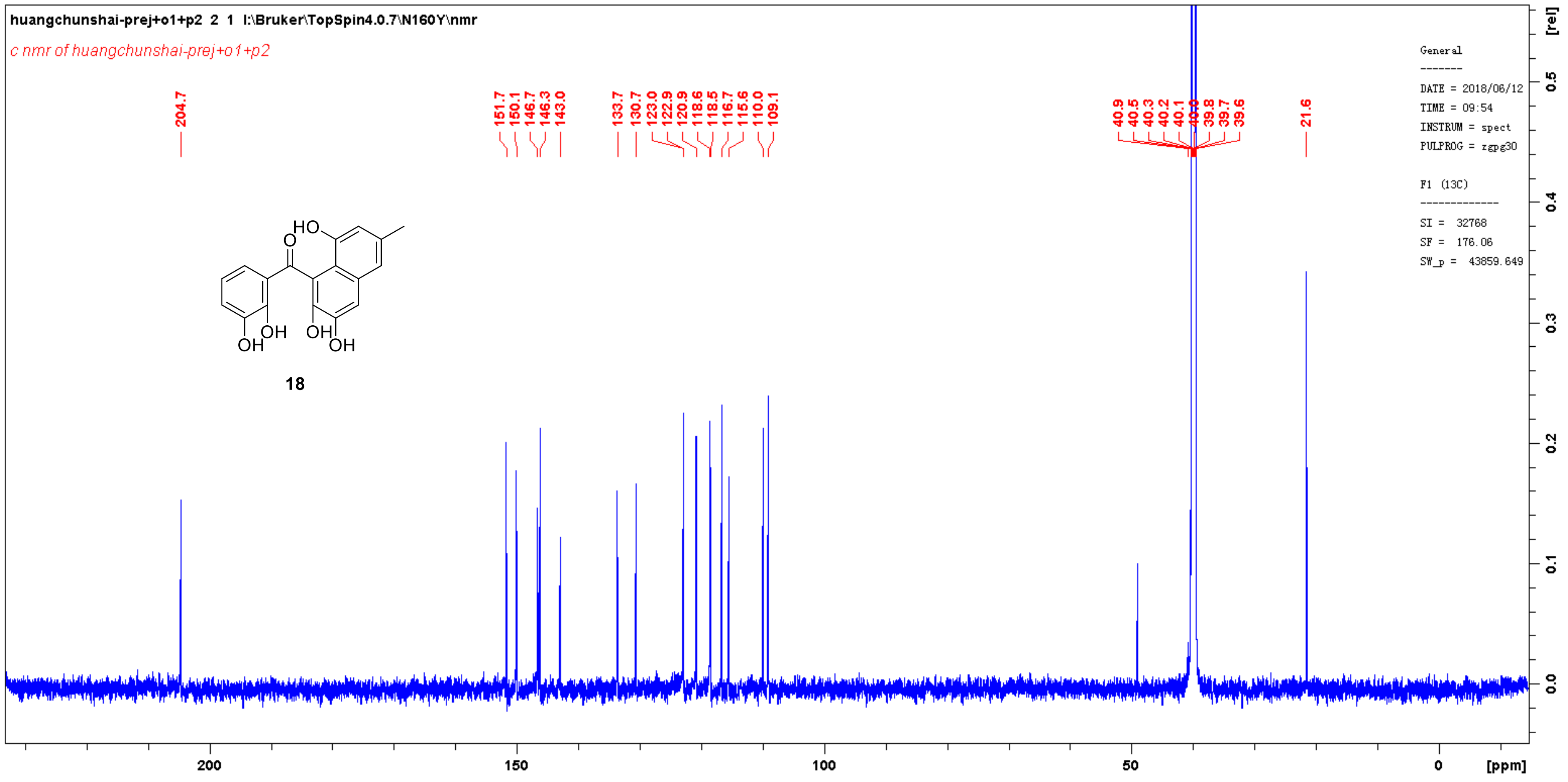

Supplementary Fig. 20. The ^{13}C (176 MHz) spectrum of fluoxanol (18) in DMSO-d_6.

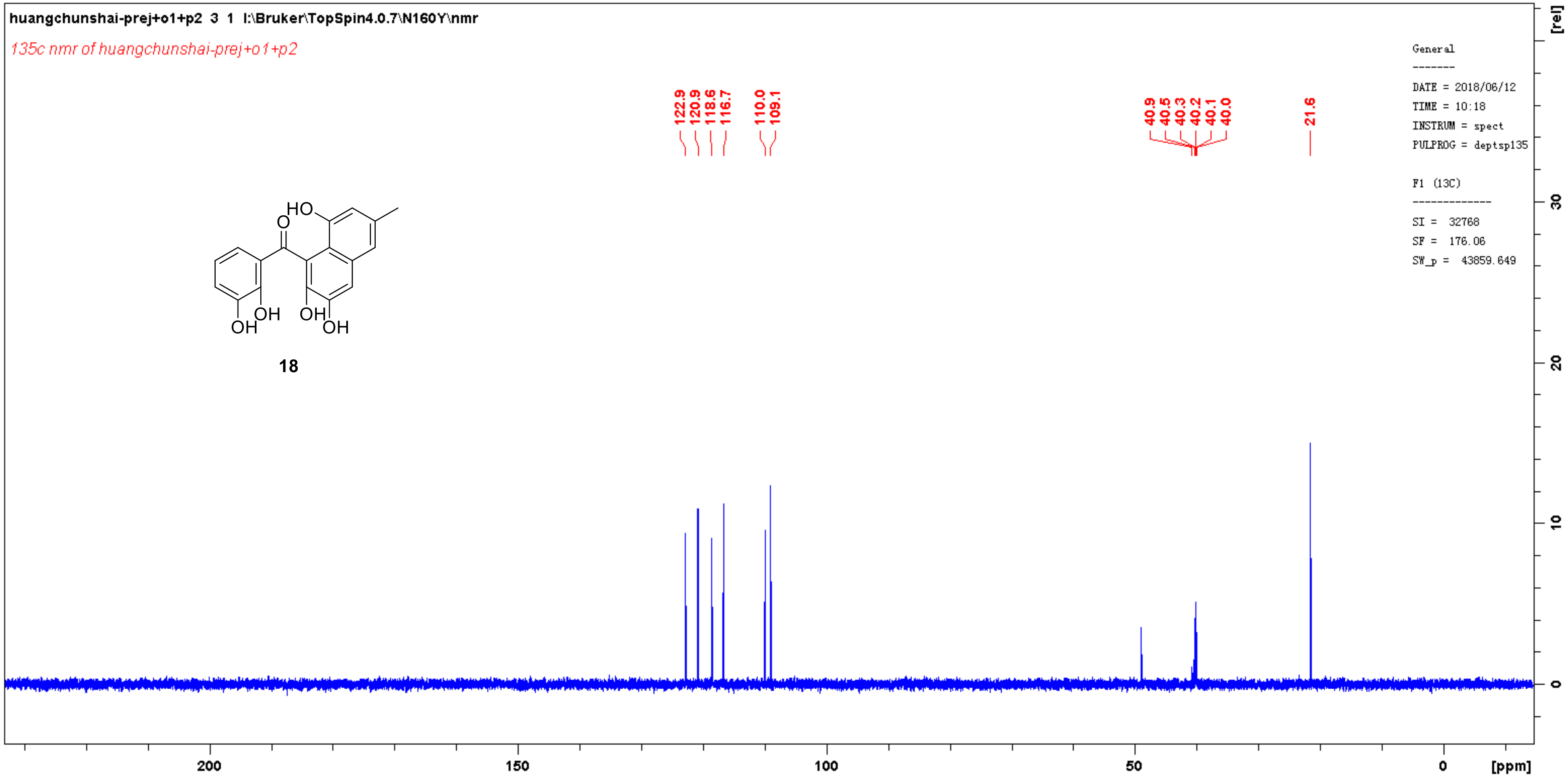

Supplementary Fig. 21. The DEPT 135 spectrum of fluoxanol (18) in DMSO-d_6.

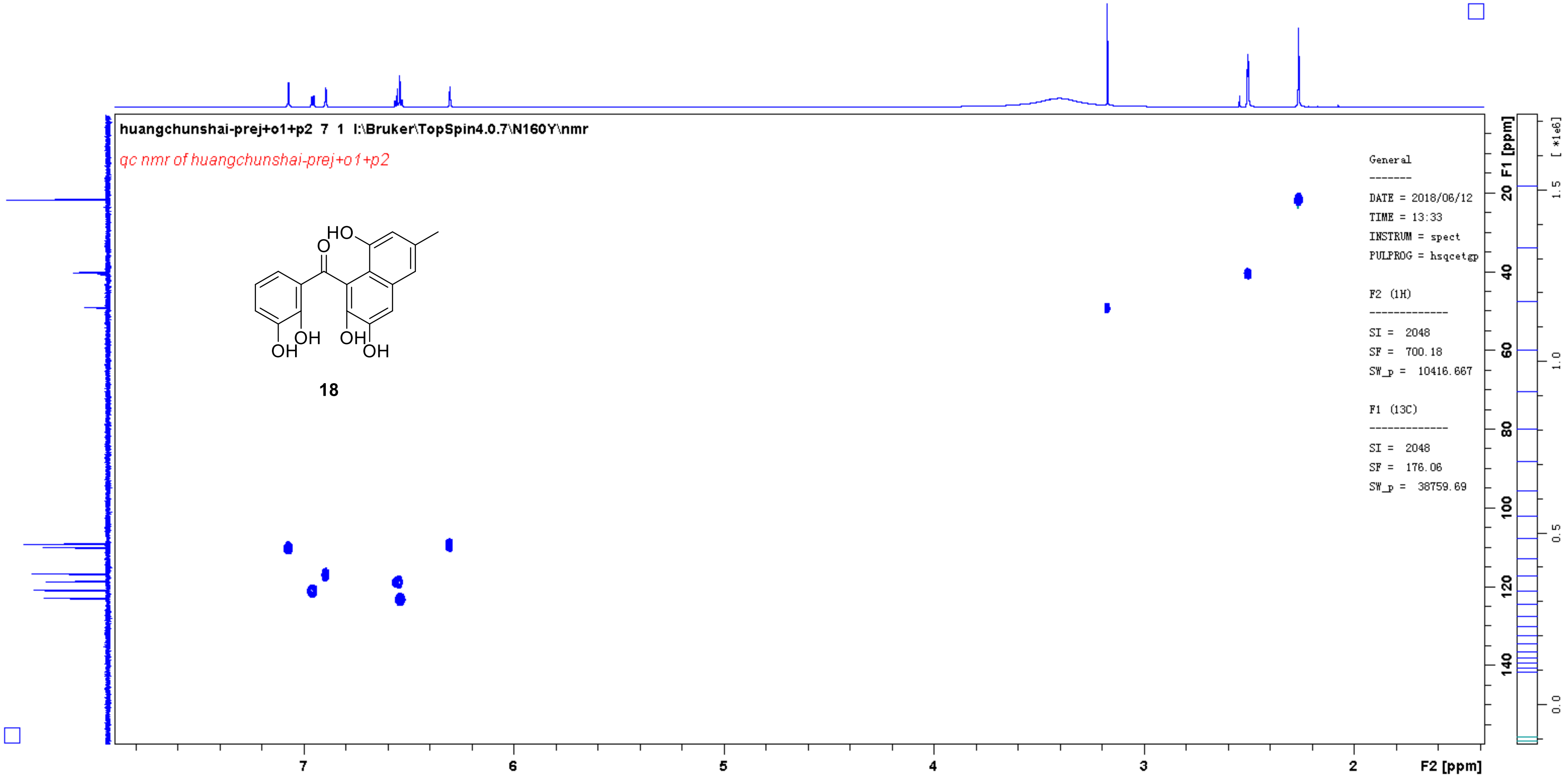

Supplementary Fig. 22. The HSQC spectrum of fluoxanol (18) in DMSO-d_6.

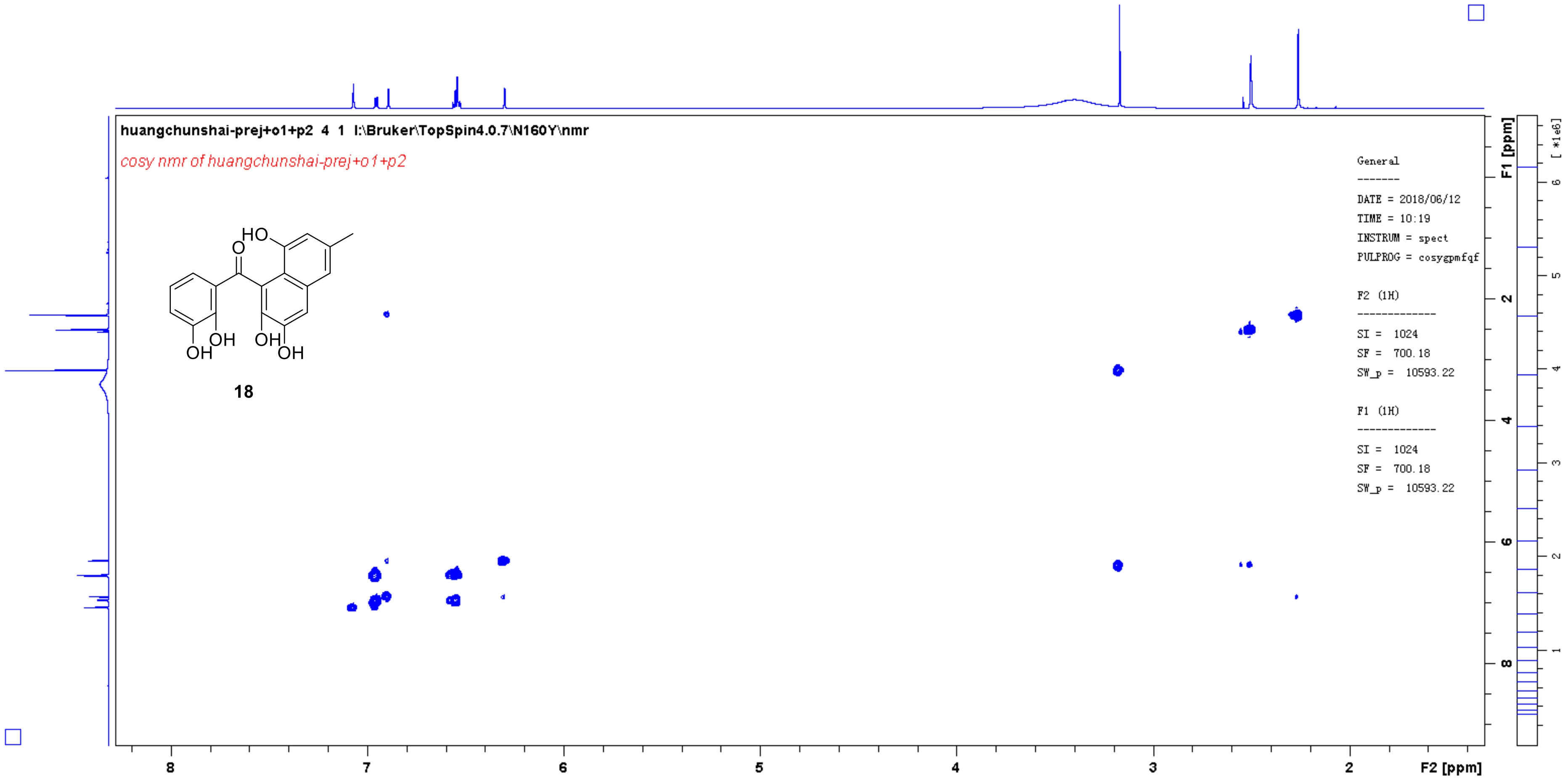

Supplementary Fig. 23. The COSY spectrum of fluoxanol (18) in DMSO-d_6.

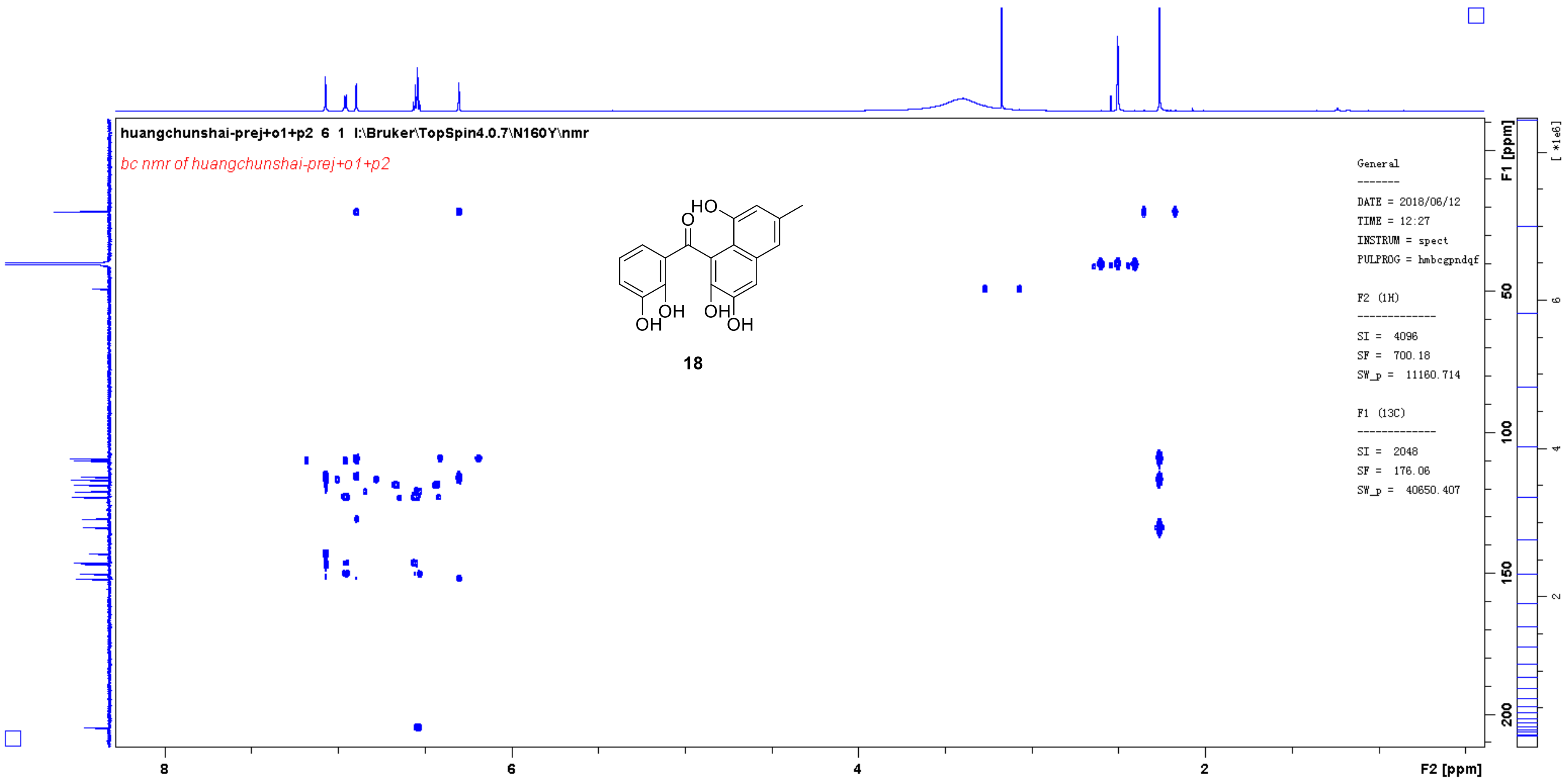

Supplementary Fig. 24. The HMBC spectrum of fluoxanol (18) in DMSO-*d*$_6$.

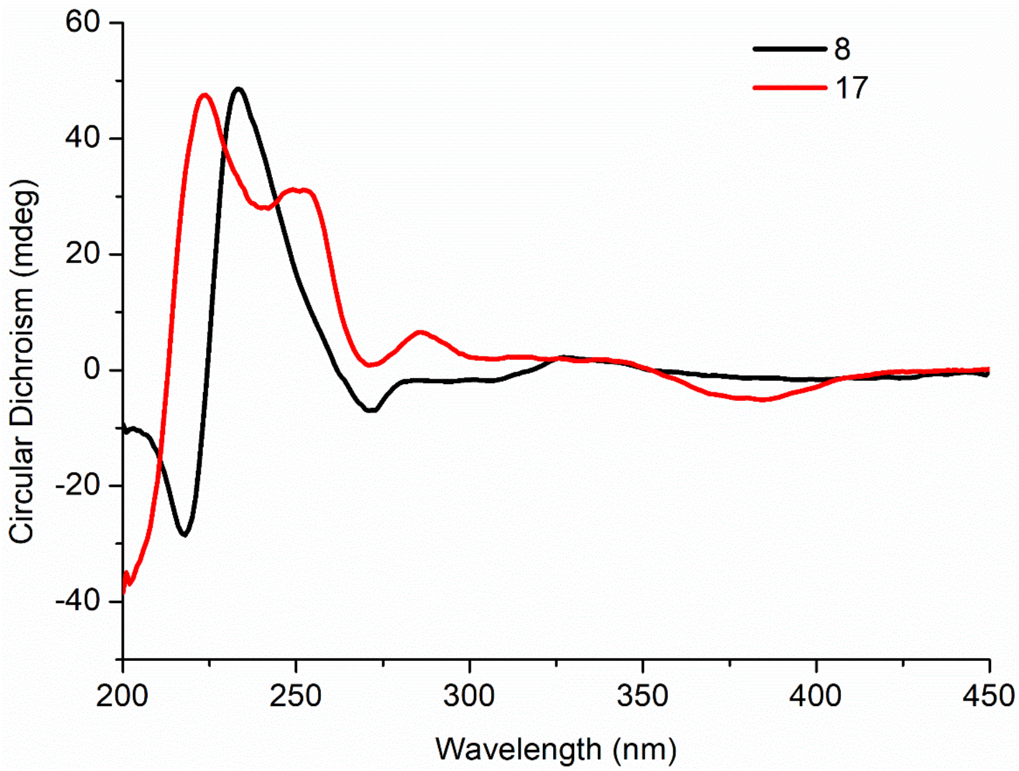

Supplementary Fig. 25. Comparison of the experimental ECD spectra of 8 and 17.

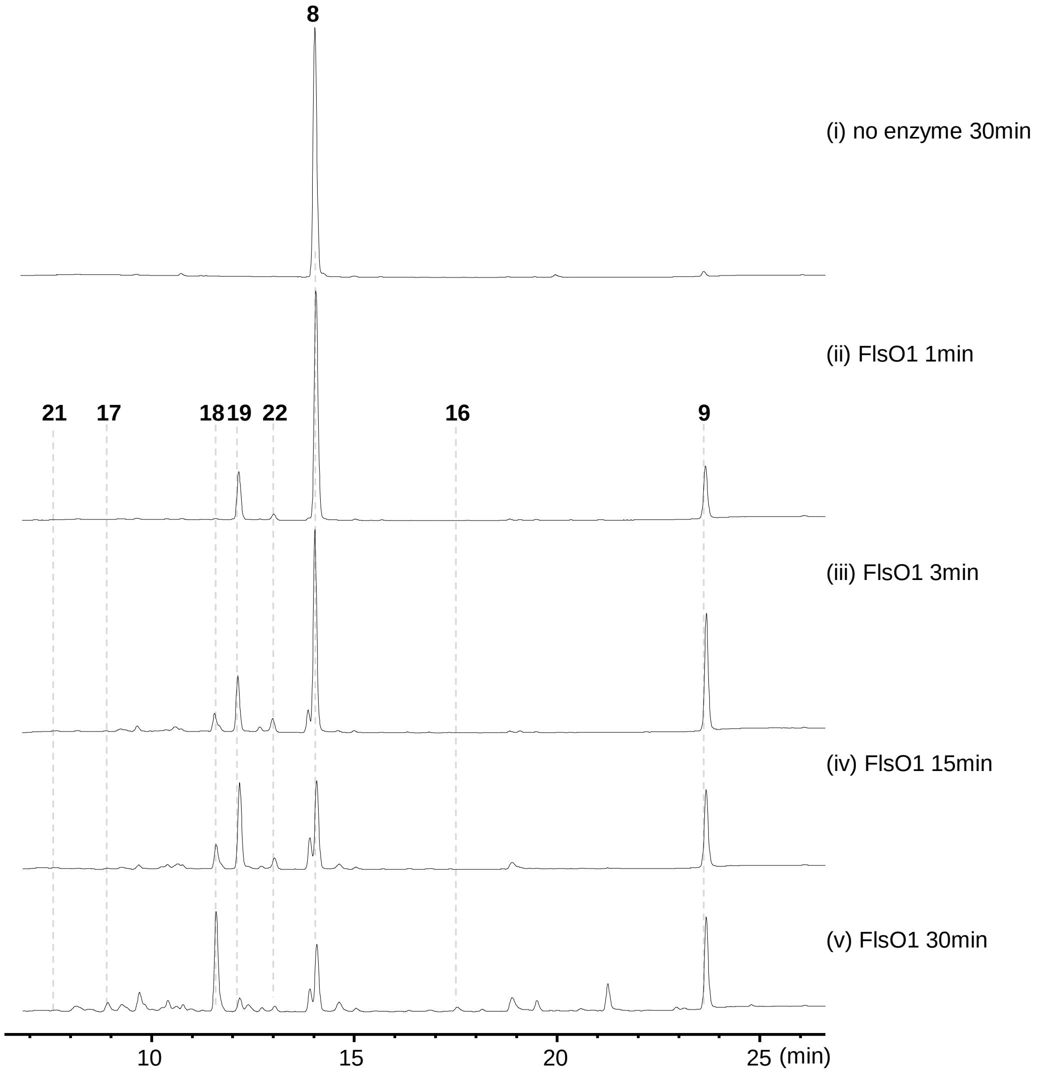

Supplementary Fig. 26. HPLC analysis of the time course assay of FlsO1 with PJM (8). The assays were performed by incubation of 200 μM PJM (**8**), 2 mM NADPH, and 10μM FlsO1, with (i) control (no enzyme) 30 min; (ii) FlsO1 1 min; (iii) FlsO1 3 min; (iv) FlsO1 15 min; (v) FlsO1 30 min, in 50 mM PBS buffers (pH 6.0) at 30 °C.

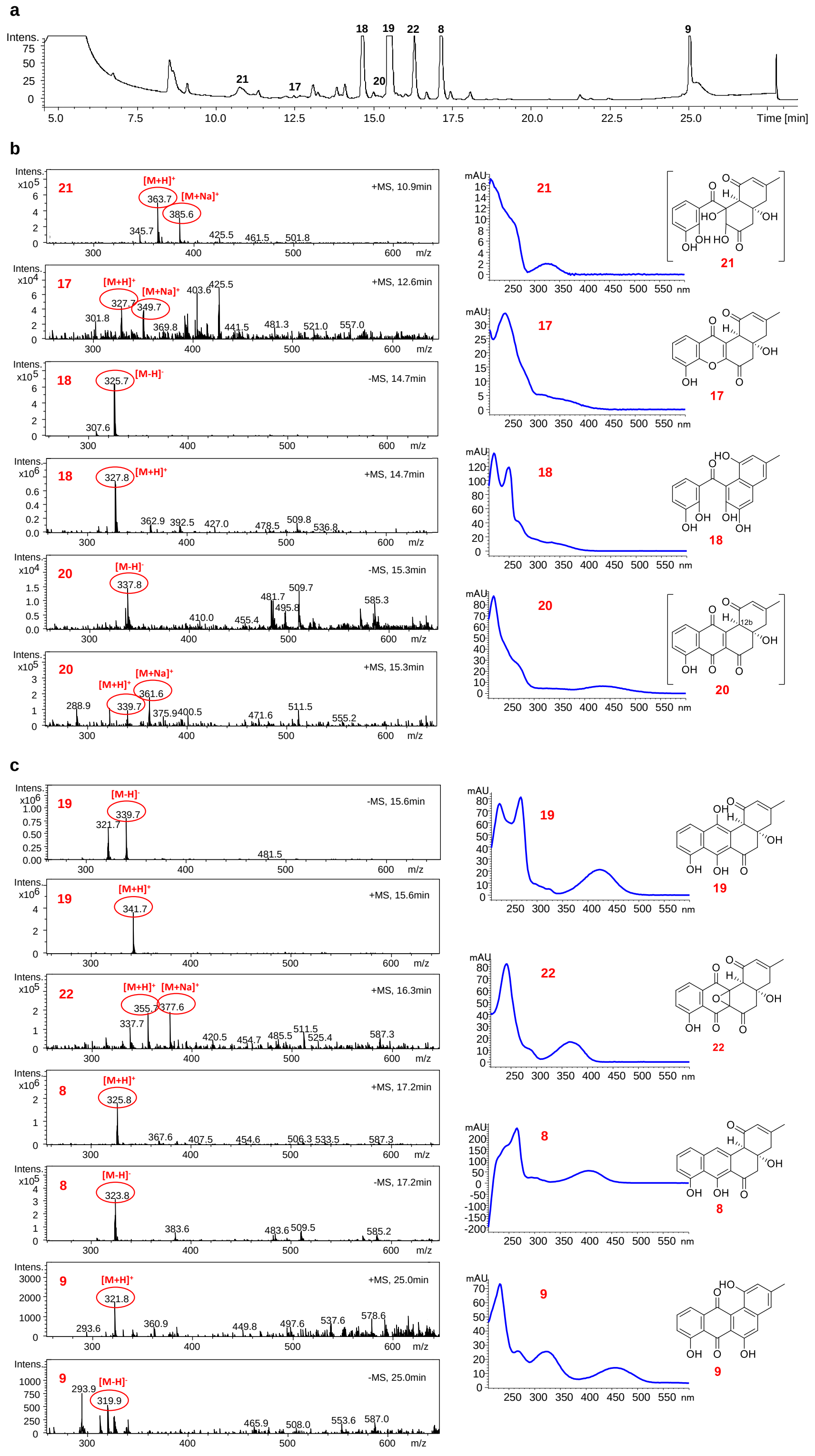

Supplementary Fig. 27. LC-MS analysis for the putative intermediated of the FlsO1 reaction with 8. The reaction was performed by incubation of 200 μM **8** in the presence of 2 mM NADPH and 10 μM FlsO1, in 50 mM PBS buffers (pH 6.0) at 30 °C for 5 min. (**a**) The HPLC analysis of FlsO1 reaction products. (**b**) The MS profiles, UV-Vis spectra and putative structures of compounds **21**, **17**, **18** and **20**. (**c**) The MS profiles, UV-Vis spectra and structures of compounds **19**, **22**, **8** and **9**.

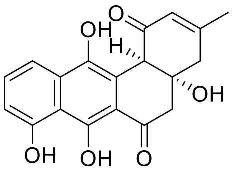

19

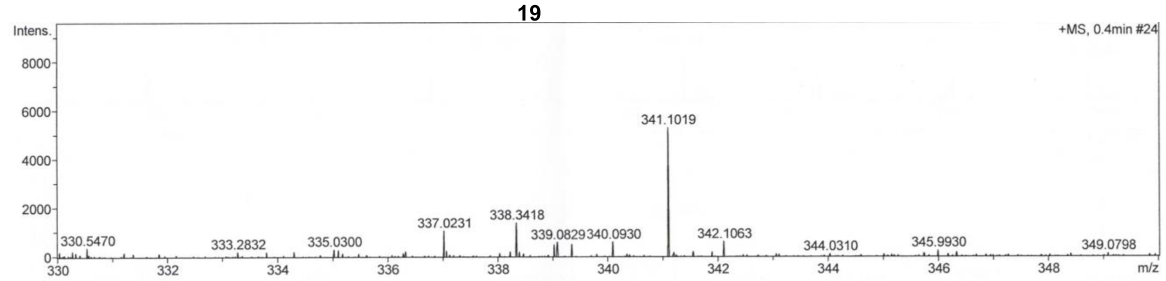

Meas. m/z	#	Ion Formula	Score	m/z	err [ppm]	err [mDa]	mSigma	rdb	e⁻ Conf	N-Rule
341.1019	1	C19H17O6	100.00	341.1020	0.2	0.1	45.5	11.5	even	ok
681.1961	1	C38H33O12	100.00	681.1967	-0.9	-0.6	16.7	22.5	even	ok

Supplementary Fig. 28. HRESIMS spectrum of 12-hydroxyl-prejadomycin (19).

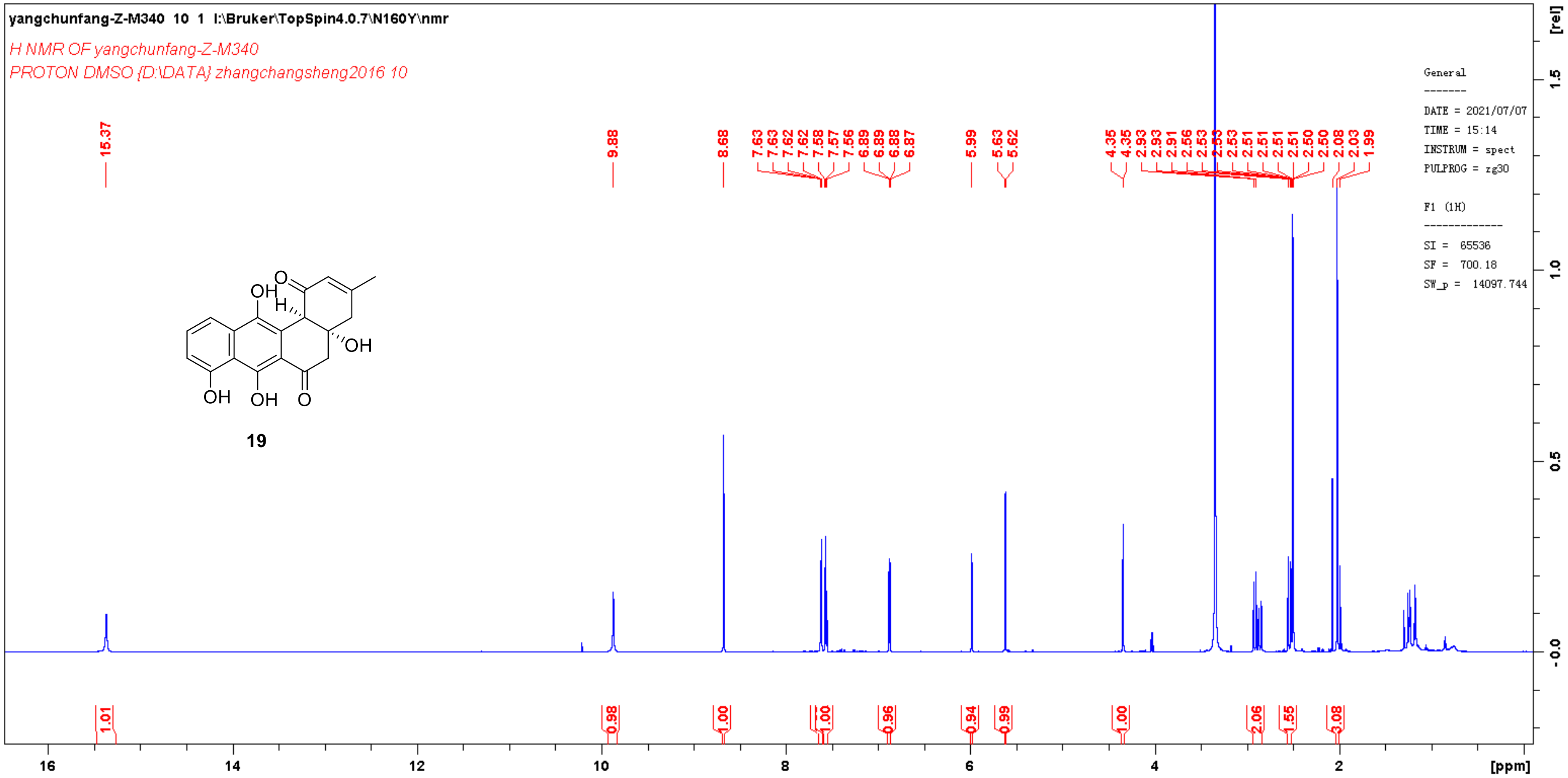

Supplementary Fig. 29. The ^{1}H NMR (700 MHz) spectrum of 12-hydroxyl-prejadomycin (19) in DMSO-d_6.

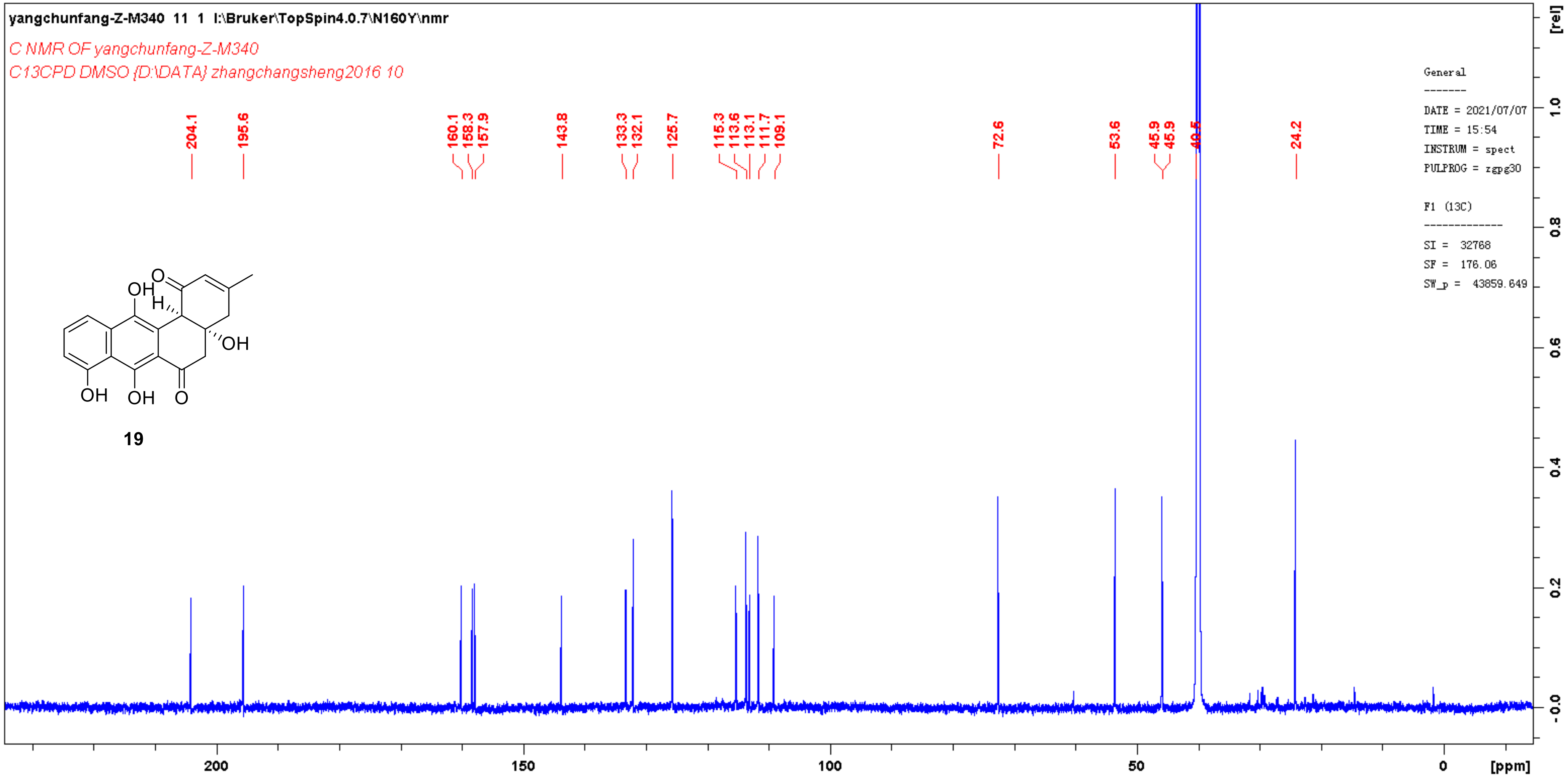

Supplementary Fig. 30. The ^{13}C (176 MHz) spectrum of 12-hydroxyl-prejadomycin (19) in DMSO-d_6.

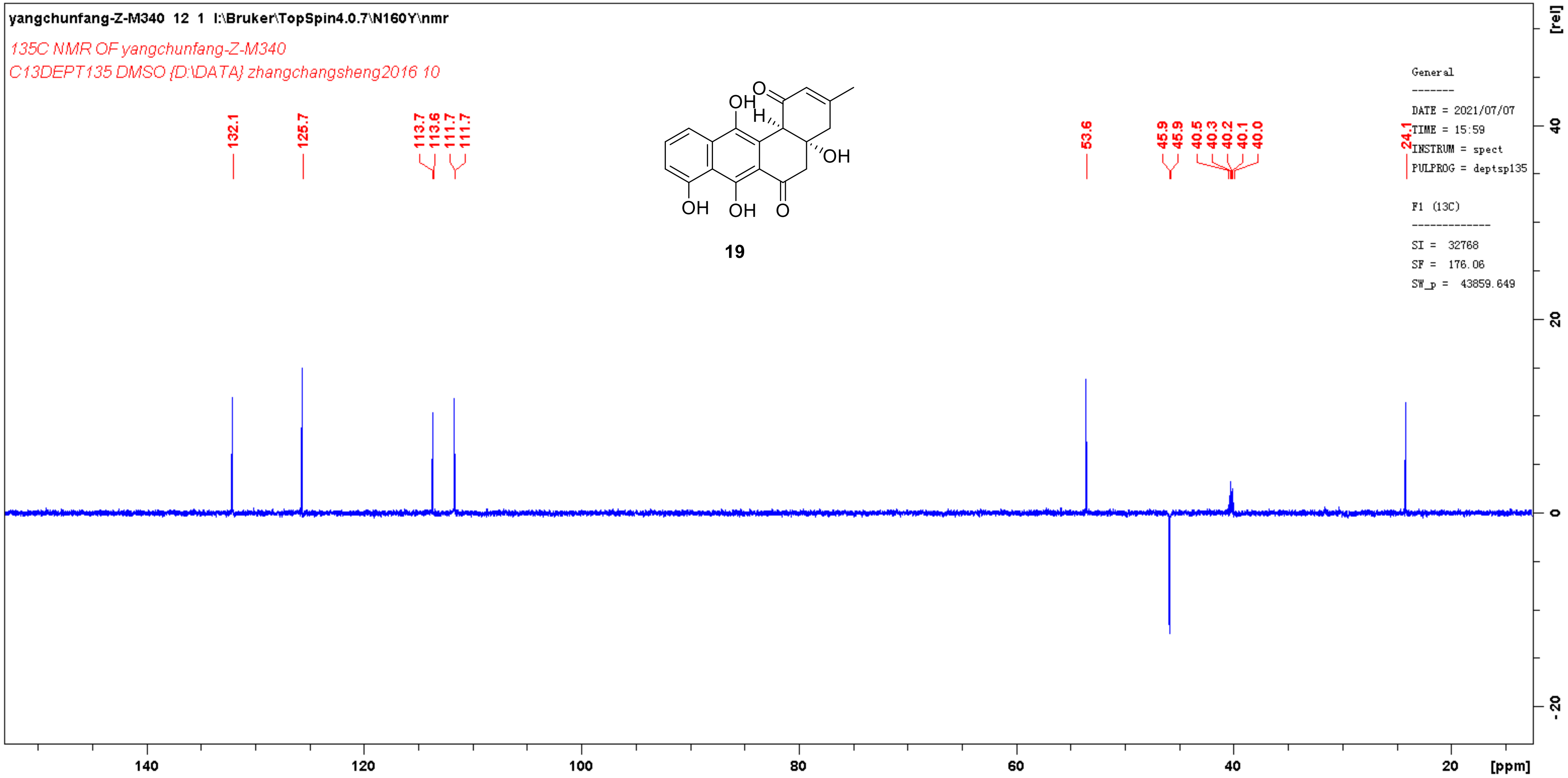

Supplementary Fig. 31. The DEPT 135 spectrum of 12-hydroxyl-prejadomycin (19) in DMSO-d_6.

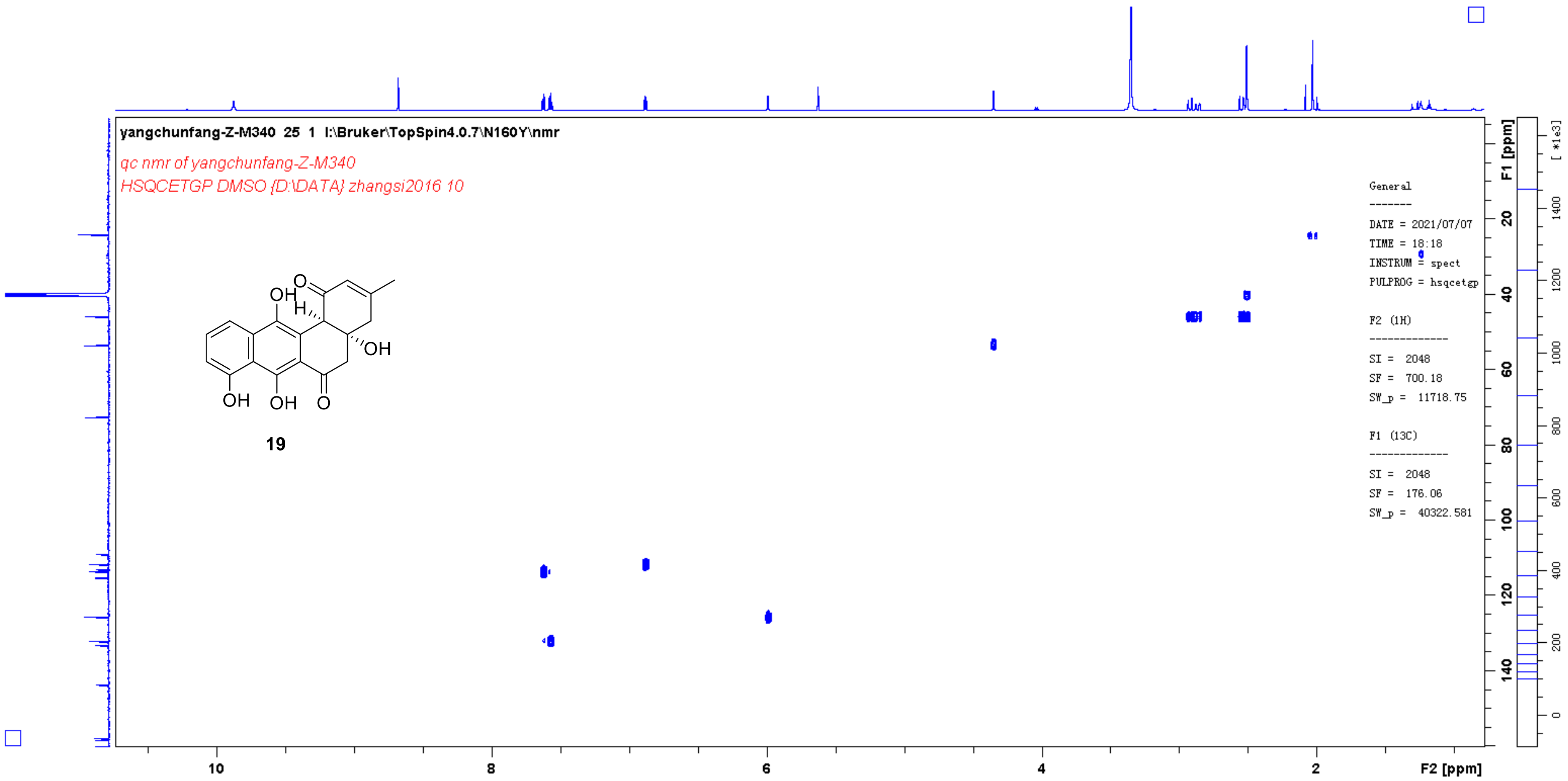

Supplementary Fig. 32. The HSQC spectrum of 12-hydroxyl-prejadomycin (19) in DMSO-d_6.

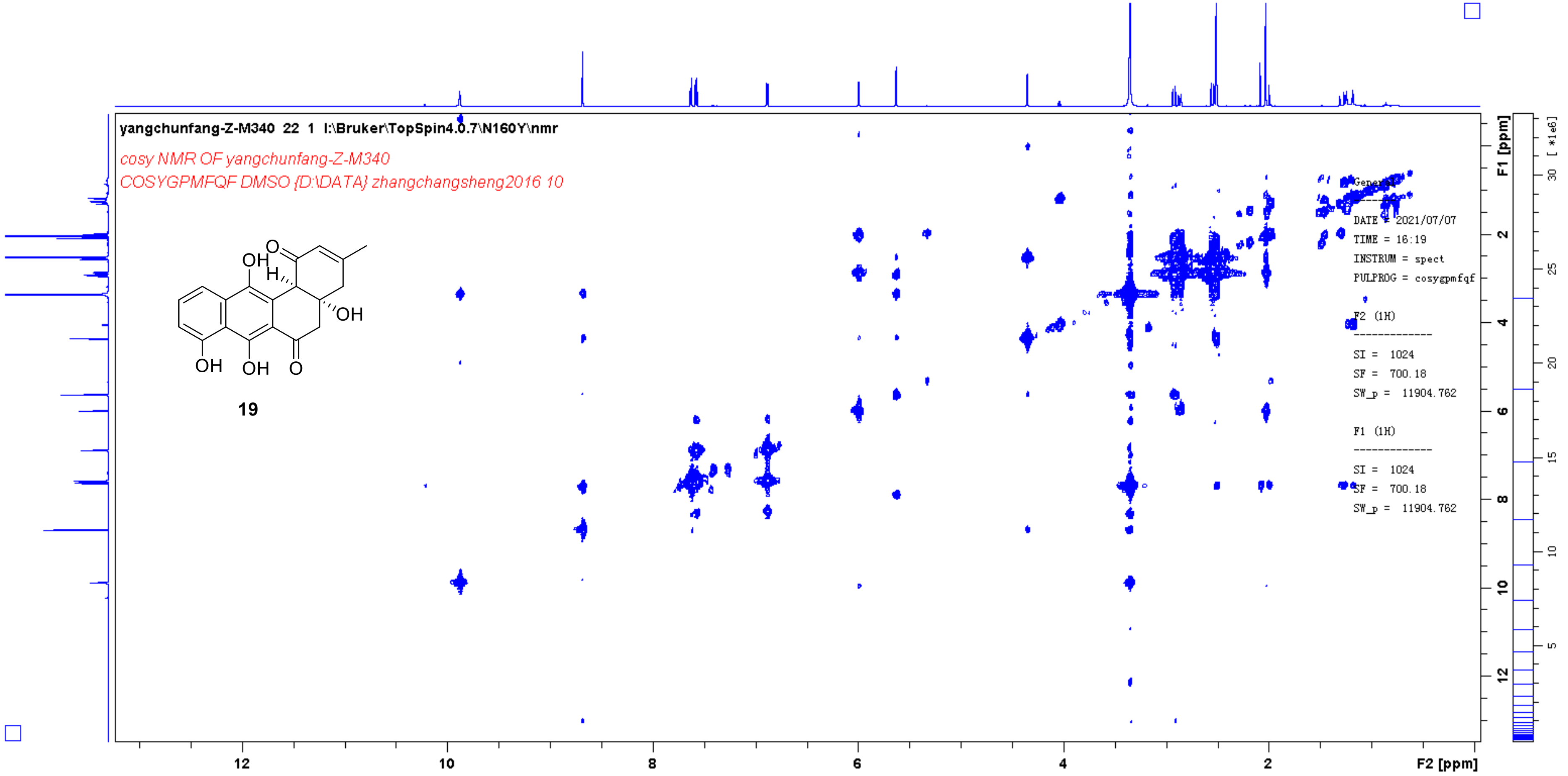

Supplementary Fig. 33. The COSY spectrum of 12-hydroxyl-prejadomycin (19) in DMSO-d_6.

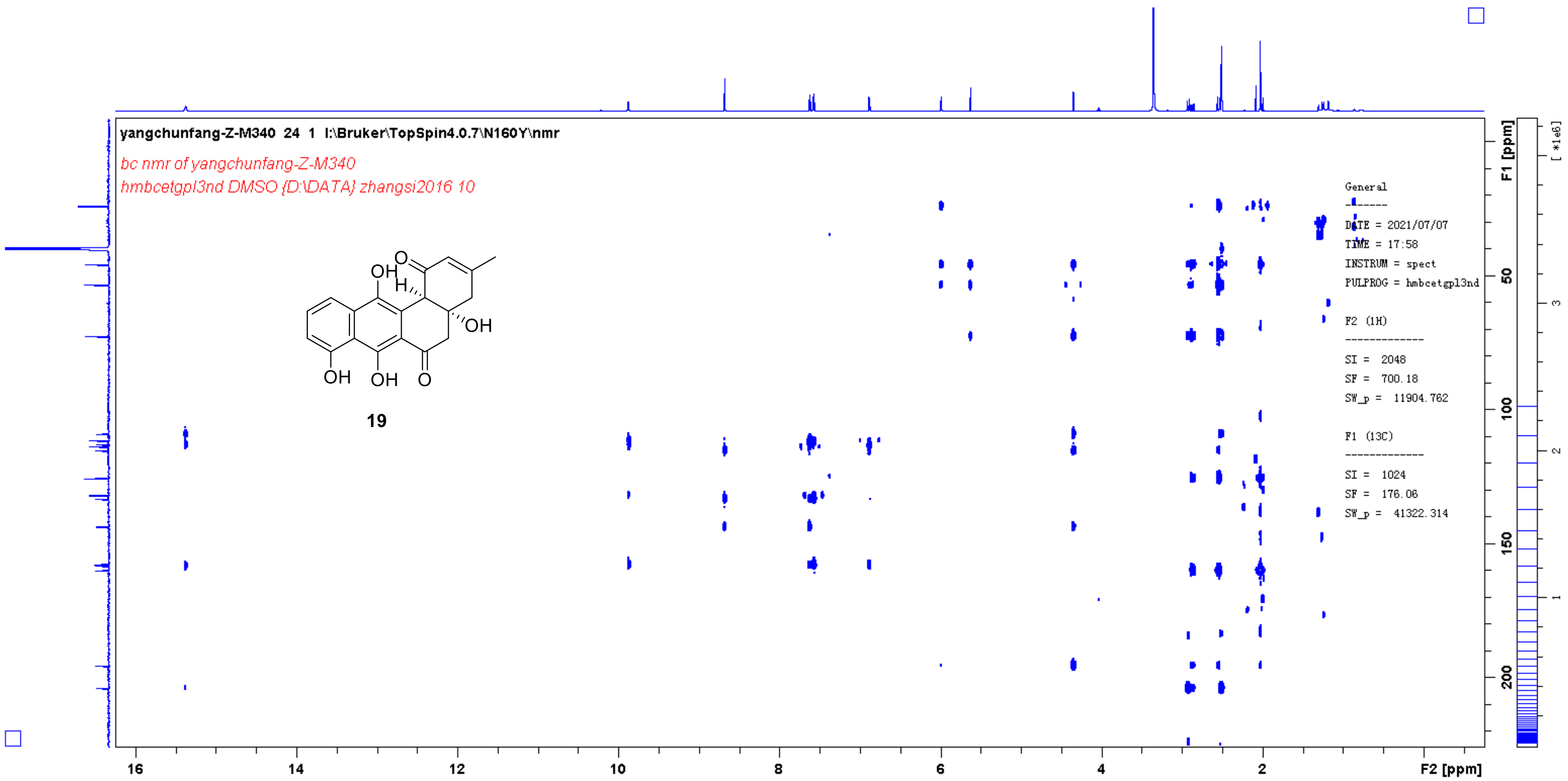

Supplementary Fig. 34. The HMBC spectrum of 12-hydroxyl-prejadomycin (19) in DMSO-d_6.

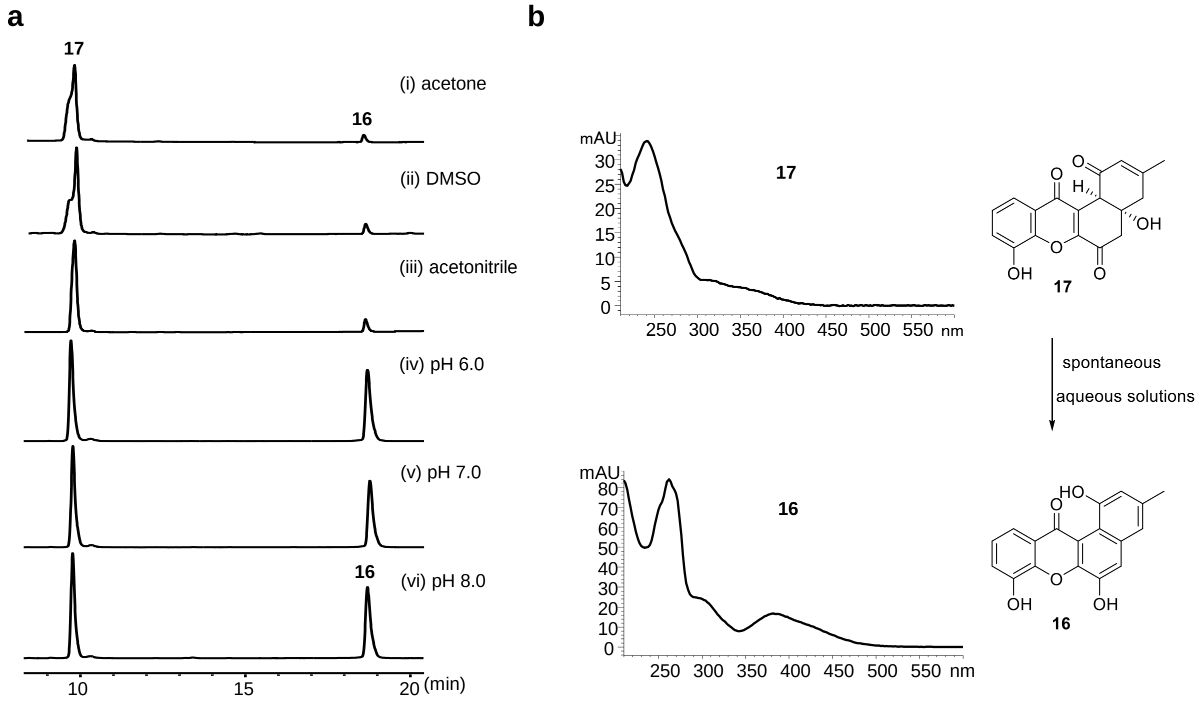

Supplementary Fig. 35. The stability of 17 under diverse conditions. (**a**) HPLC analysis for the stability of **17** in diverse solvents (i) acetone; (ii) DMSO; (iii) acetonitrile; and in 50 mM PBS buffers of (iv) pH 6.0; (v) pH 7.0; (vi) pH 8.0 at 30 °C for 2h. (**b**) The structures and UV-Vis spectra of **16** and **17**.

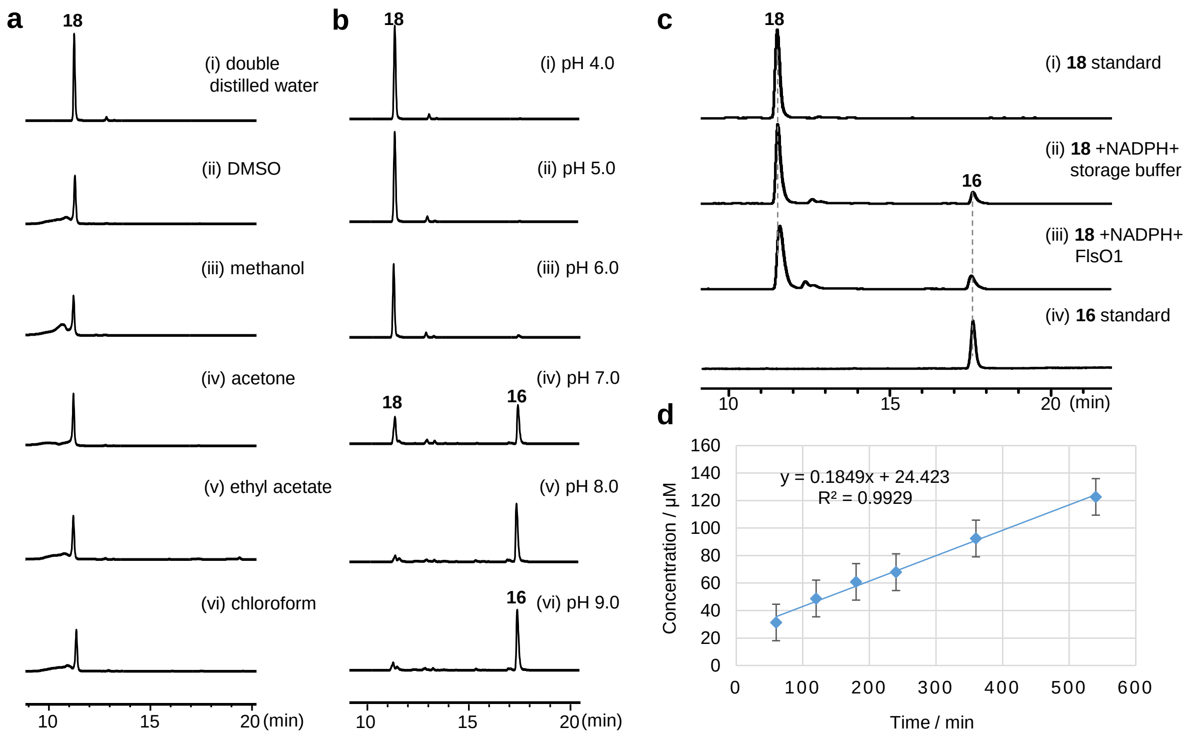

Supplementary Fig. 36. The stability of 18 in diverse solvents and the spontaneous conversation of 18 to 16. (**a**) HPLC analysis of **18** treated in diverse solvents at 30 °C for 10 h. (**b**) HPLC analysis of **18** treated in 50 mM buffers (citric acid or sodium citrate buffer) with pH values ranging from 4.0 to 9.0 at 30 °C for 5 h. (**c**) HPLC analysis of the reaction of FlsO1 with **18** in the presence of 2 mM NADPH, in 50 mM PBS buffers (pH 7.0) at 30 °C for 2 h. (i) **18** standard; (ii) control (no enzyme); (iii) 10 μM FlsO1; (iv) **16** standard. (**d**) Determination of the rate of the spontaneous conversation of **18** to **16** in 50 mM PBS buffers (pH 7.0). To test the conversion rate of nonenzymatic conversion of **18** to **16**, a time course assay was conducted by incubation of 100 μM **18** at 30 °C in 50 mM phosphate buffer (pH 7.0), and samples were taken at 1 h, 2 h, 3 h, 4 h, 6 h, and 9 h. The assays were done in triplicates. Data are presented as mean values ± SD (standard deviation). Source data are provided as a Source Data file. The curve of the incubation time versus the remaining concentrations of **18** was obtained by comparing with the concentration of FST C (**7**) as an internal standard.

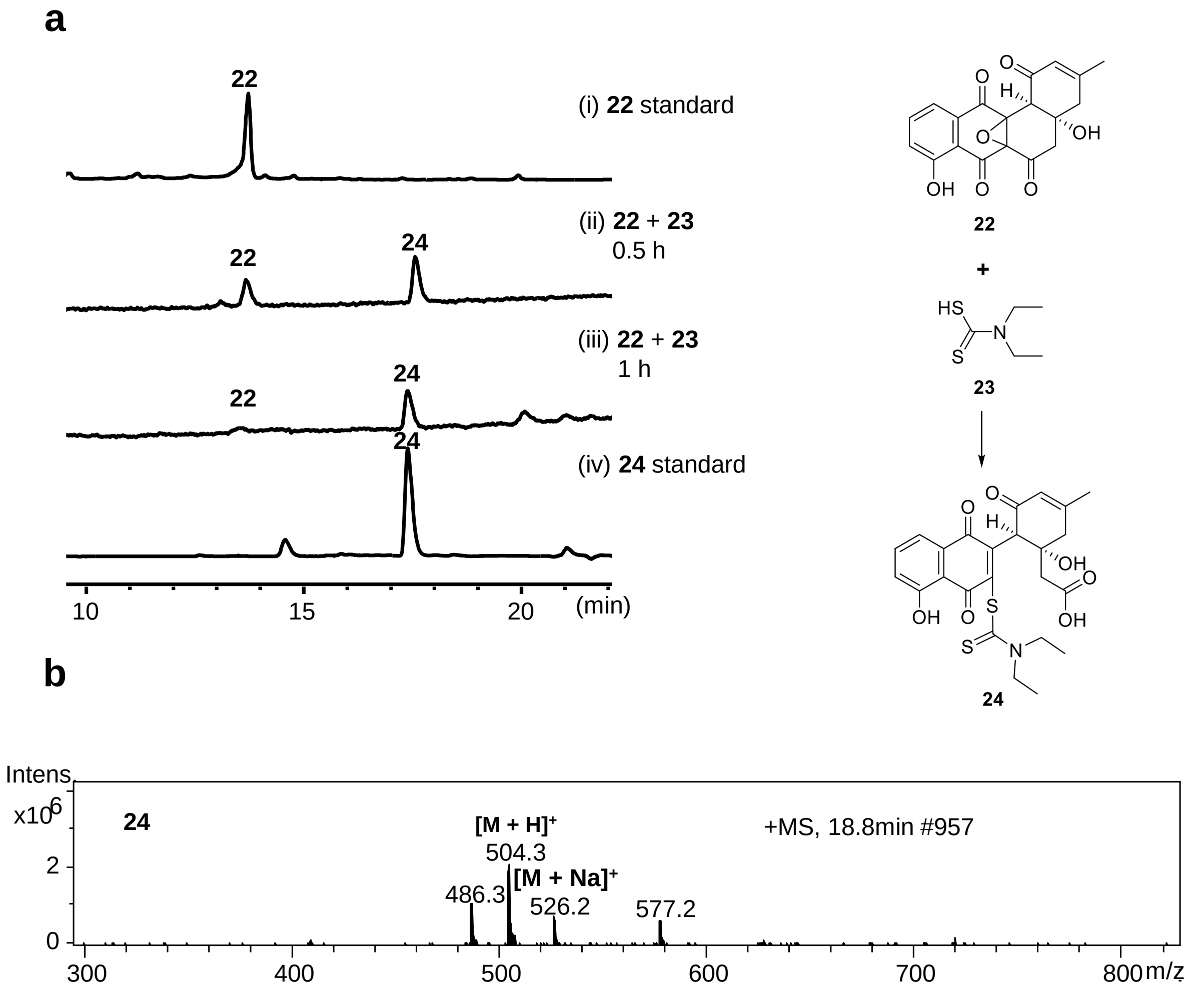

Supplementary Fig. 37. HPLC analysis of the reaction of 22 with DTC (23) and the MS analysis of the captured product 24. (**a**) HPLC analysis of the reaction of **22** and **23** in 50 mM PBS buffers (pH 7.0) at 30 °C. (i) **22** standard; (ii) **22** + **23** for 0.5 h; (iii) **22** + **23** for 1 h; (iv) **24** standard. (**b**) The MS analysis of the captured product **24**.

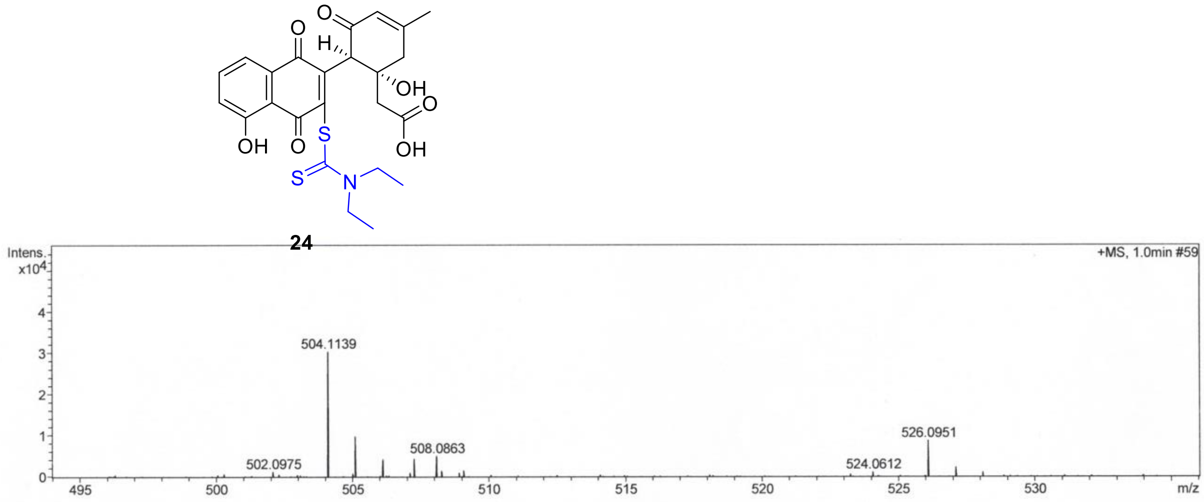

Meas. m/z	#	Ion Formula	Score	m/z	err [ppm]	err [mDa]	mSigma	rdb	e⁻ Conf	N-Rule
504.1139	1	C24H26NO7S2	100.00	504.1145	1.2	0.6	17.2	12.5	even	ok
526.0951	1	C24H25NNaO7S2	100.00	526.0965	2.5	1.3	10.5	12.5	even	ok

Supplementary Fig. 38. HRESIMS spectrum of DTC-fluostacid A (24).

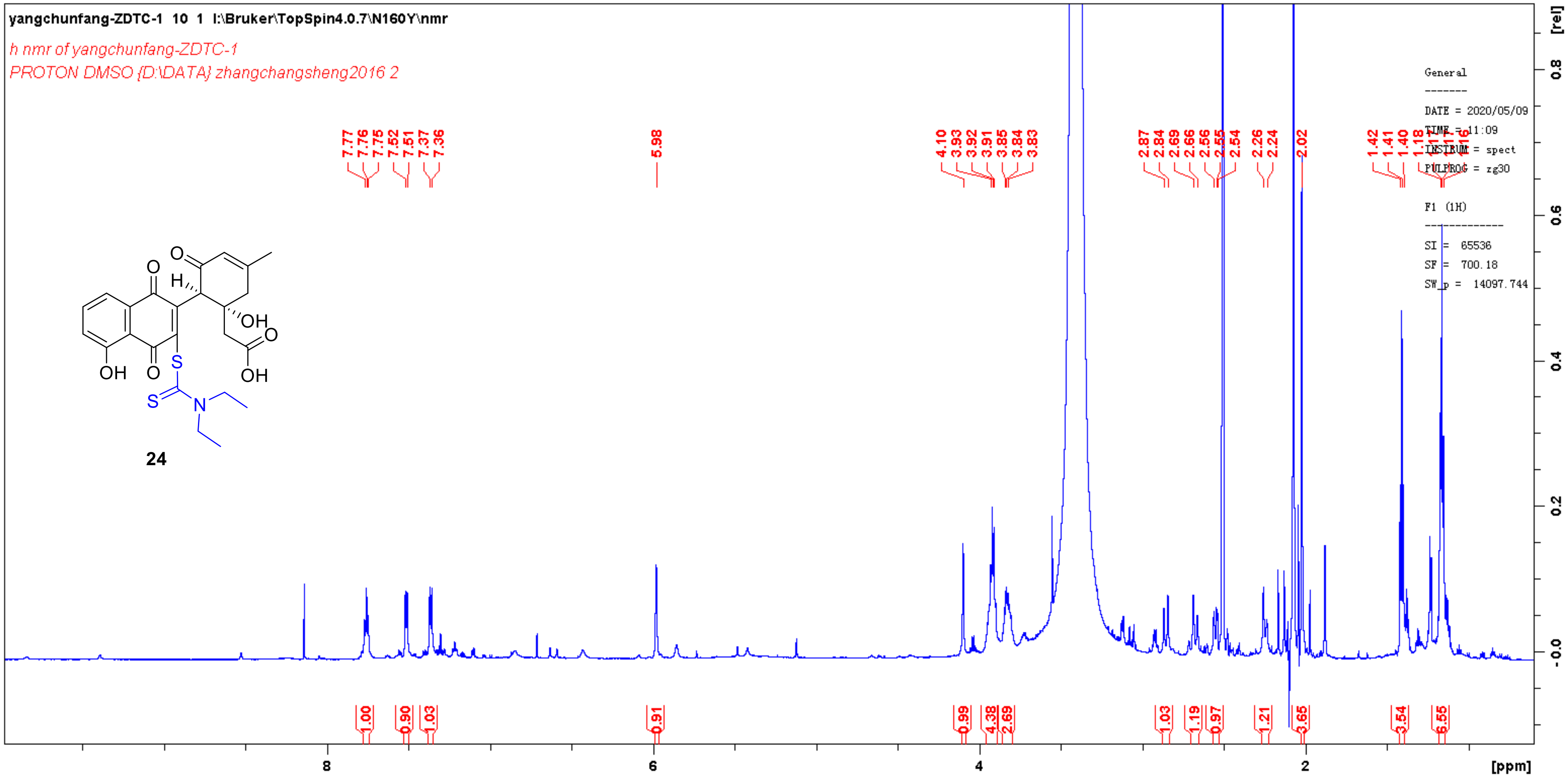

Supplementary Fig. 39. The ^{1}H NMR (700 MHz) spectrum of DTC-fluostacid A (24) in DMSO-d_6.

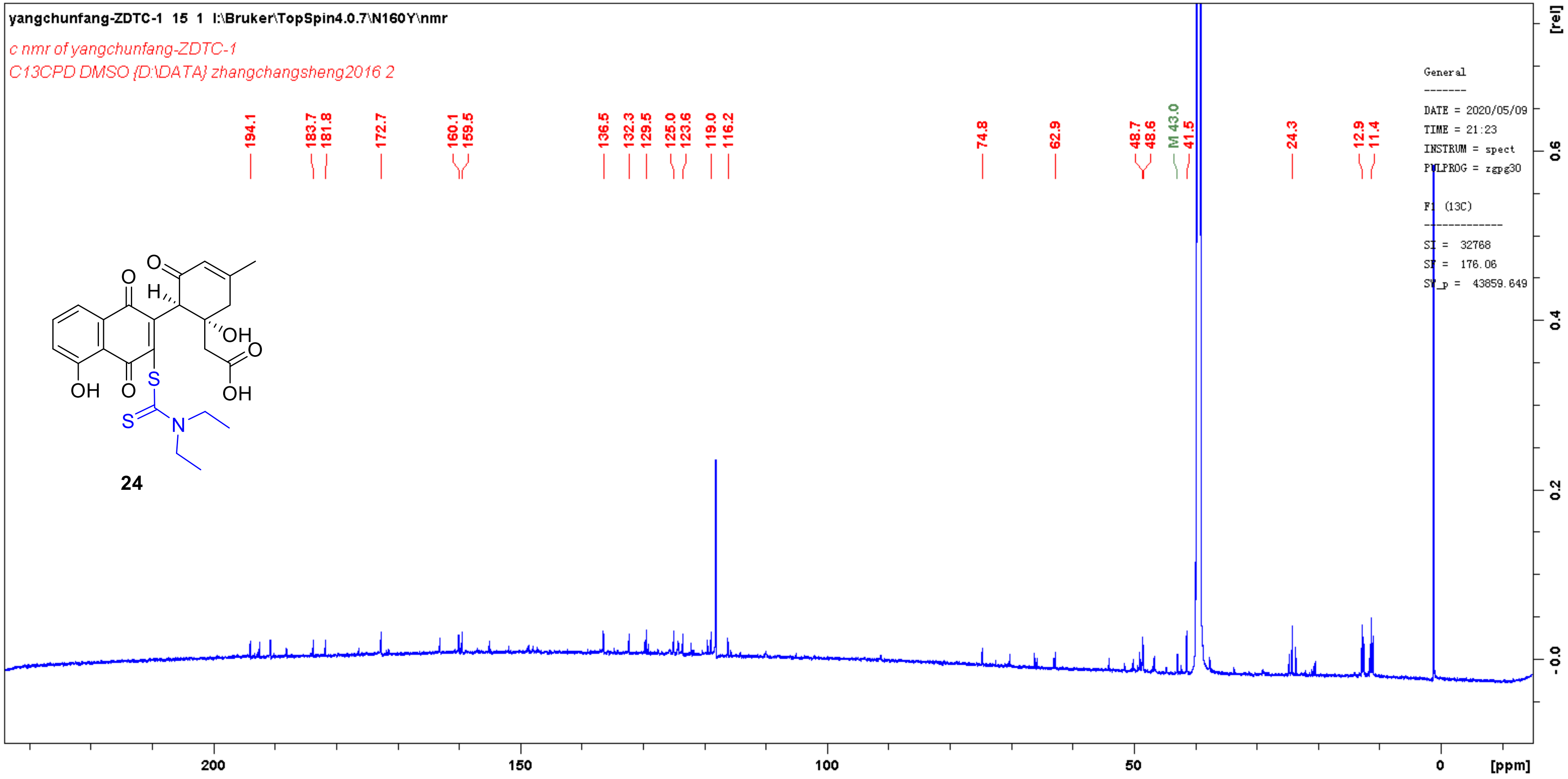

Supplementary Fig. 40. The ^{13}C (176 MHz) spectrum of DTC-fluostacid A (24) in DMSO-d_6.

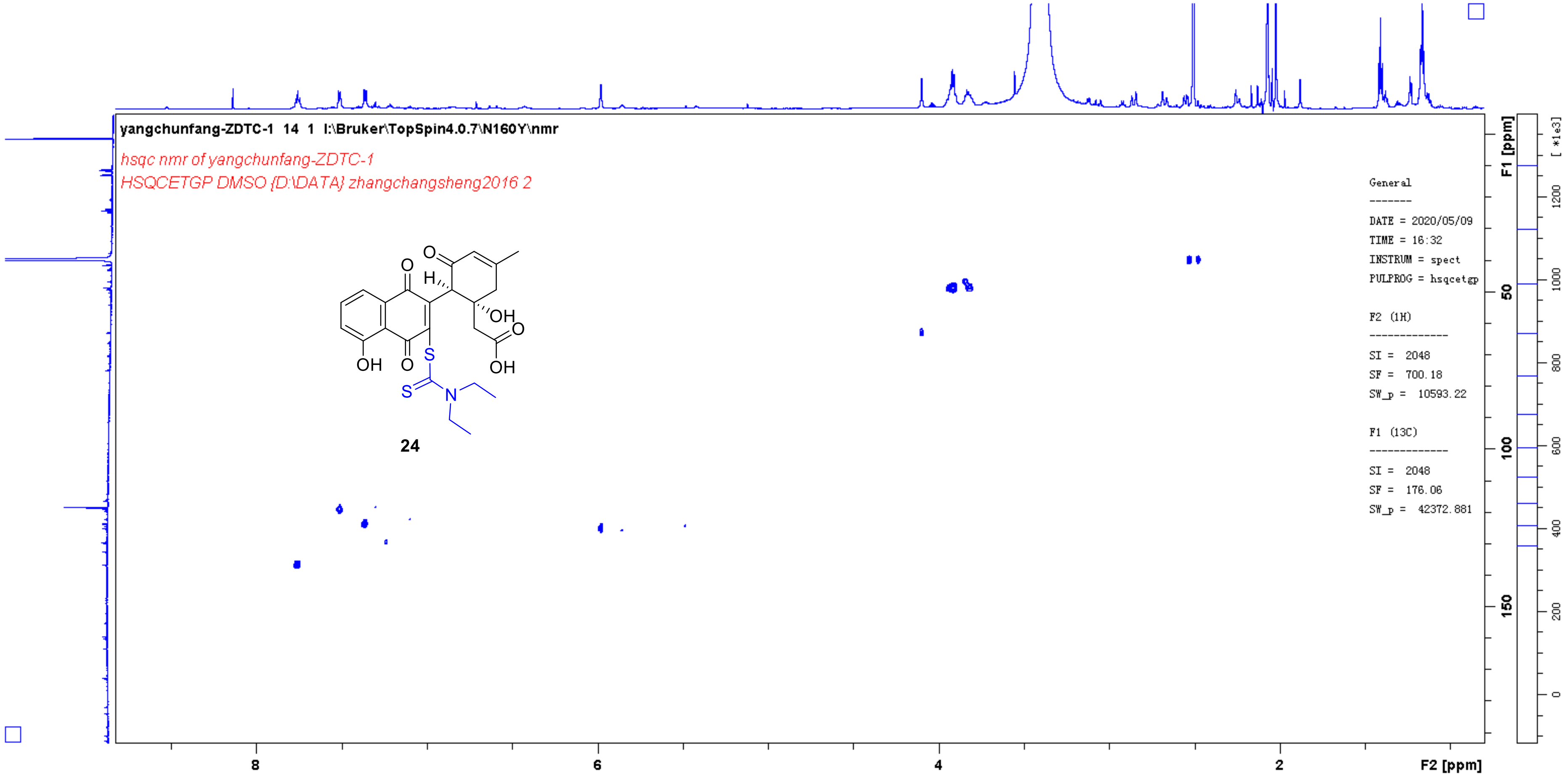

Supplementary Fig. 41. The HSQC spectrum of DTC-fluostacid A (24) in DMSO-d_6.

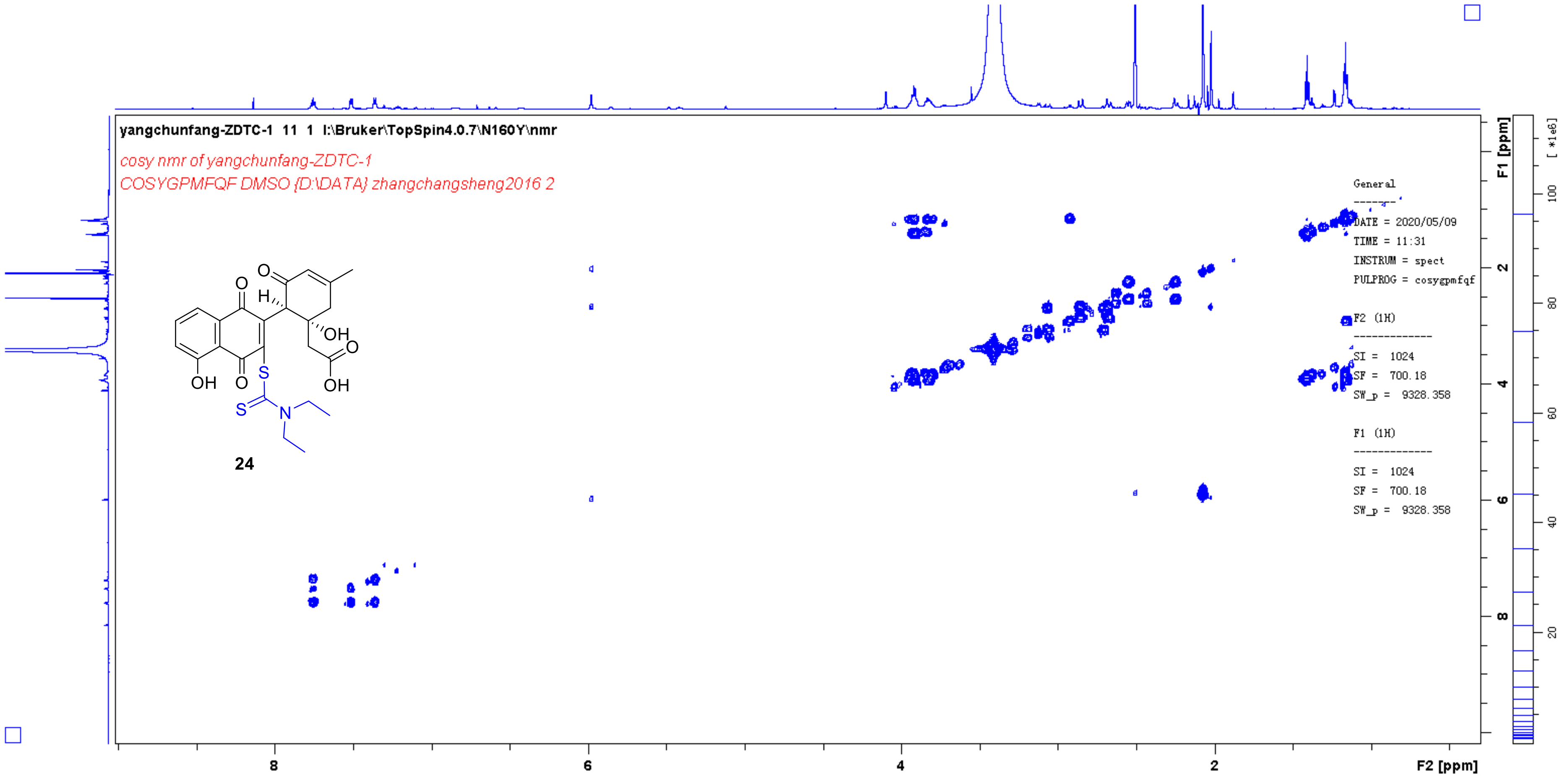

Supplementary Fig. 42. The COSY spectrum of DTC-fluostacid A (24)in DMSO-d_6.

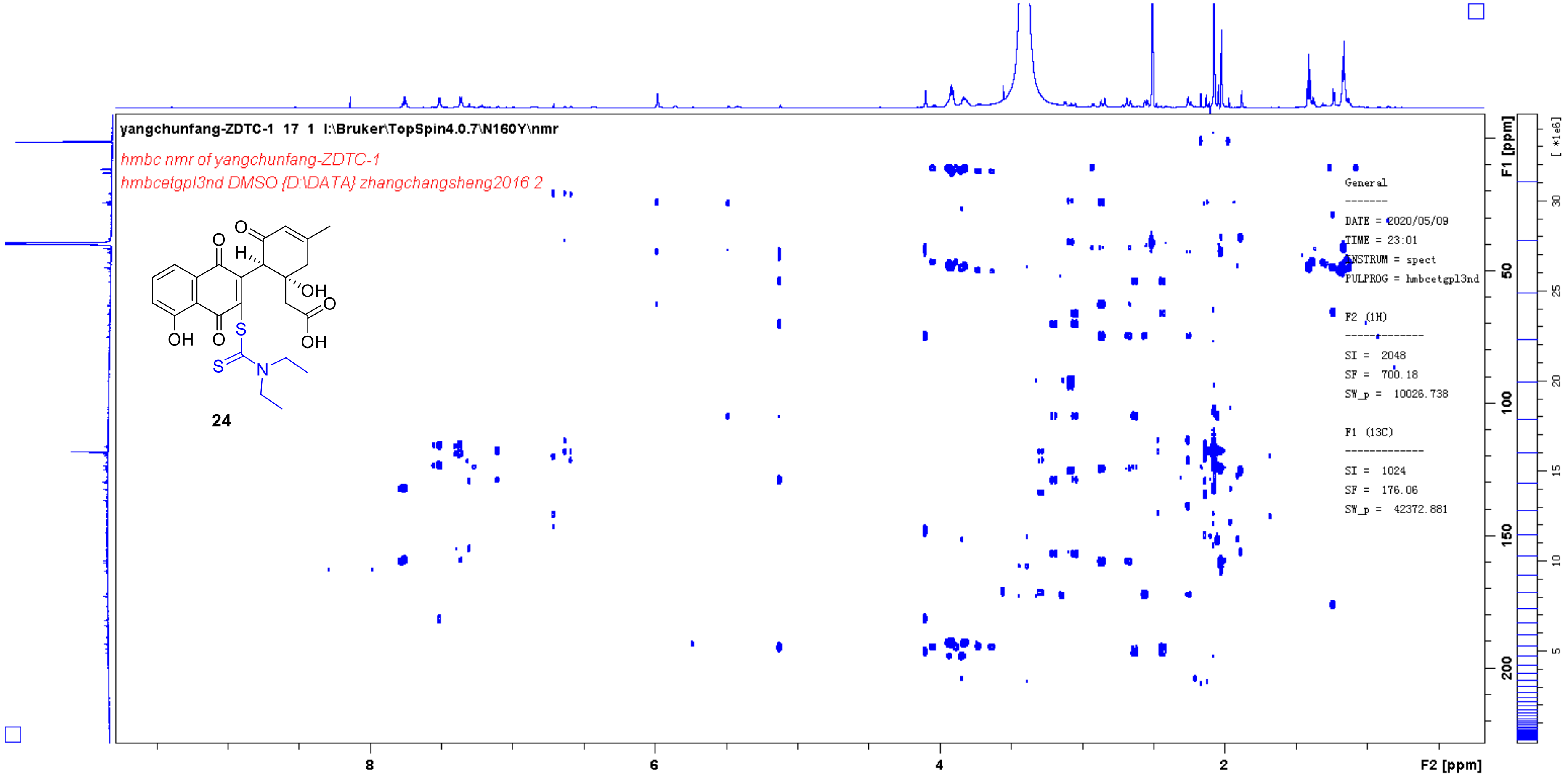

Supplementary Fig. 43. The HMBC spectrum of DTC-fluostacid A (24) in DMSO-d_6.

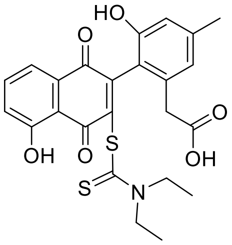

25

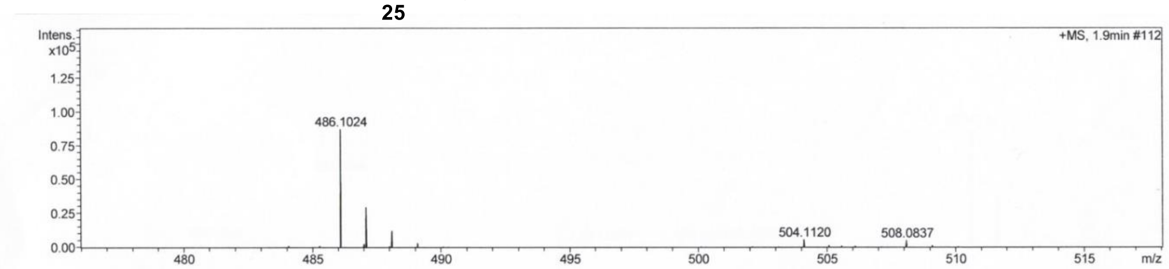

Meas. m/z	#	Ion Formula	Score	m/z	err [ppm]	err [mDa]	mSigma	rdb	e⁻ Conf	N-Rule
486.1024	1	C24H24NO6S2	100.00	486.1040	3.2	1.6	24.1	13.5	even	ok
508.0837	1	C24H23NNaO6S2	100.00	508.0859	-4.4	-2.2	13.8	13.5	even	ok

Supplementary Fig. 44. HRESIMS spectrum of DTC-fluostacid B (25).

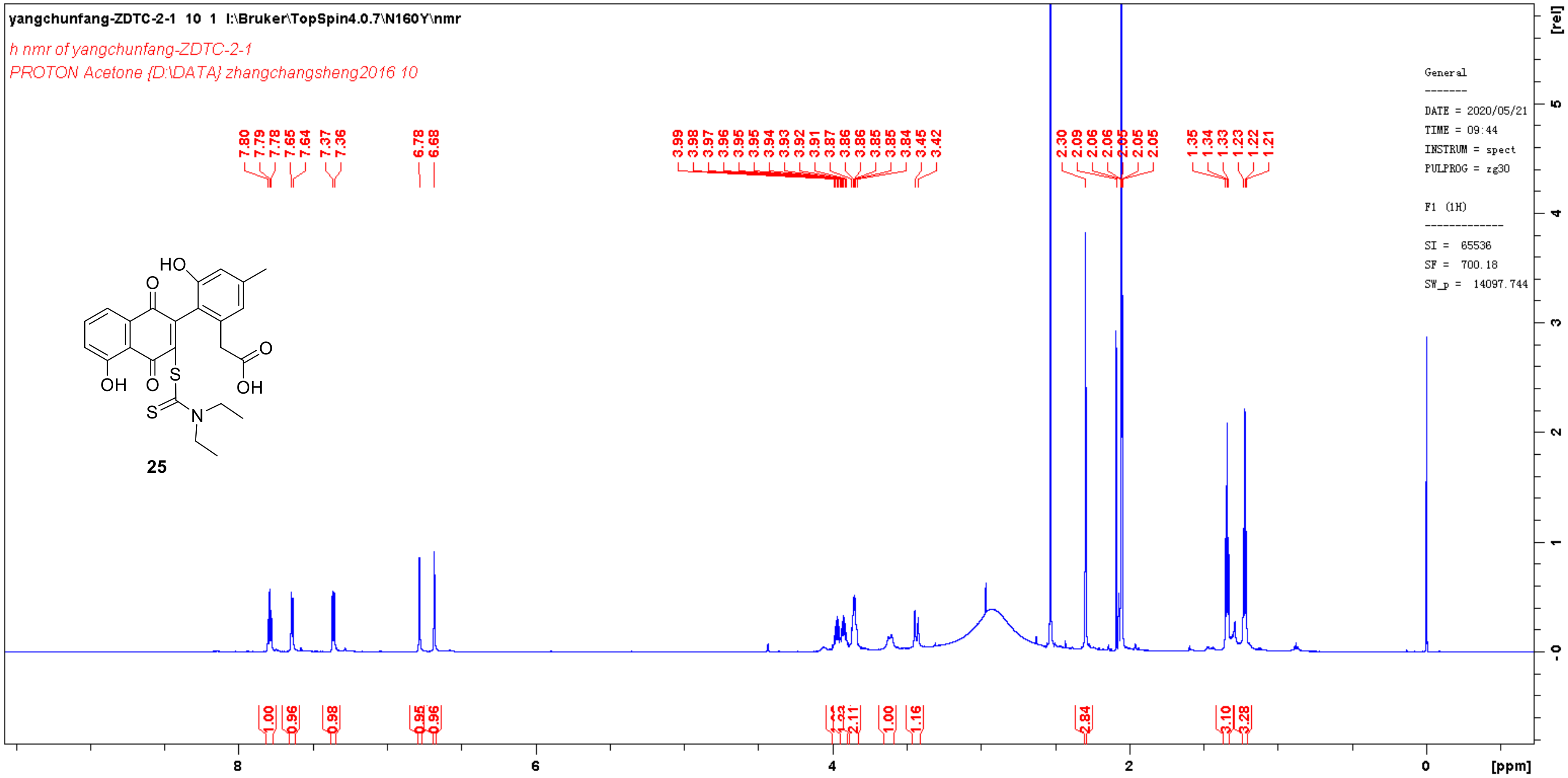

Supplementary Fig. 45. The ^{1}H NMR (700 MHz) spectrum of DTC-fluostacid B (25) in acetone-d_6.

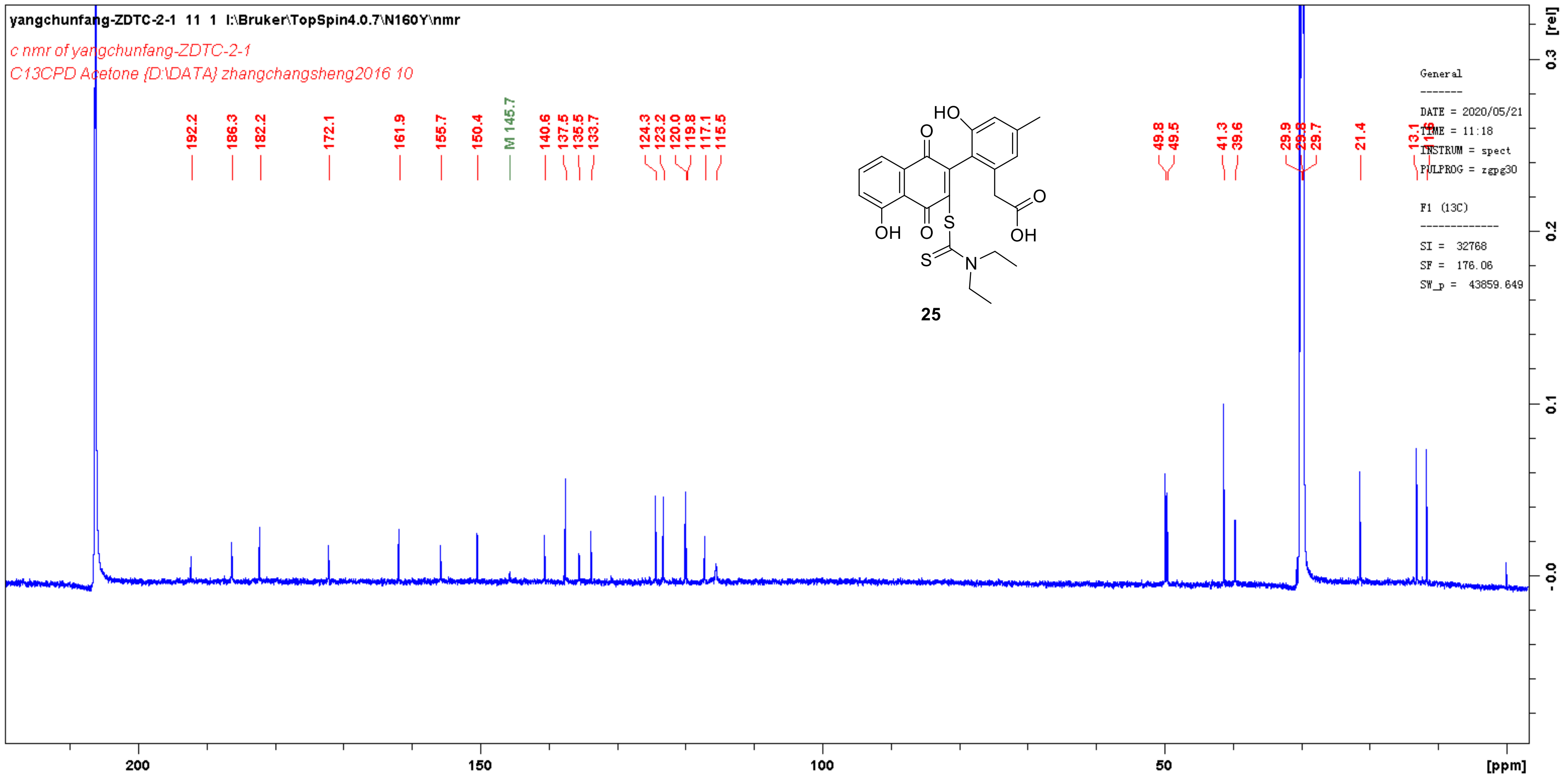

Supplementary Fig. 46. The ^{13}C (176 MHz) spectrum of DTC-fluostacid B (25) in acetone-d_6.

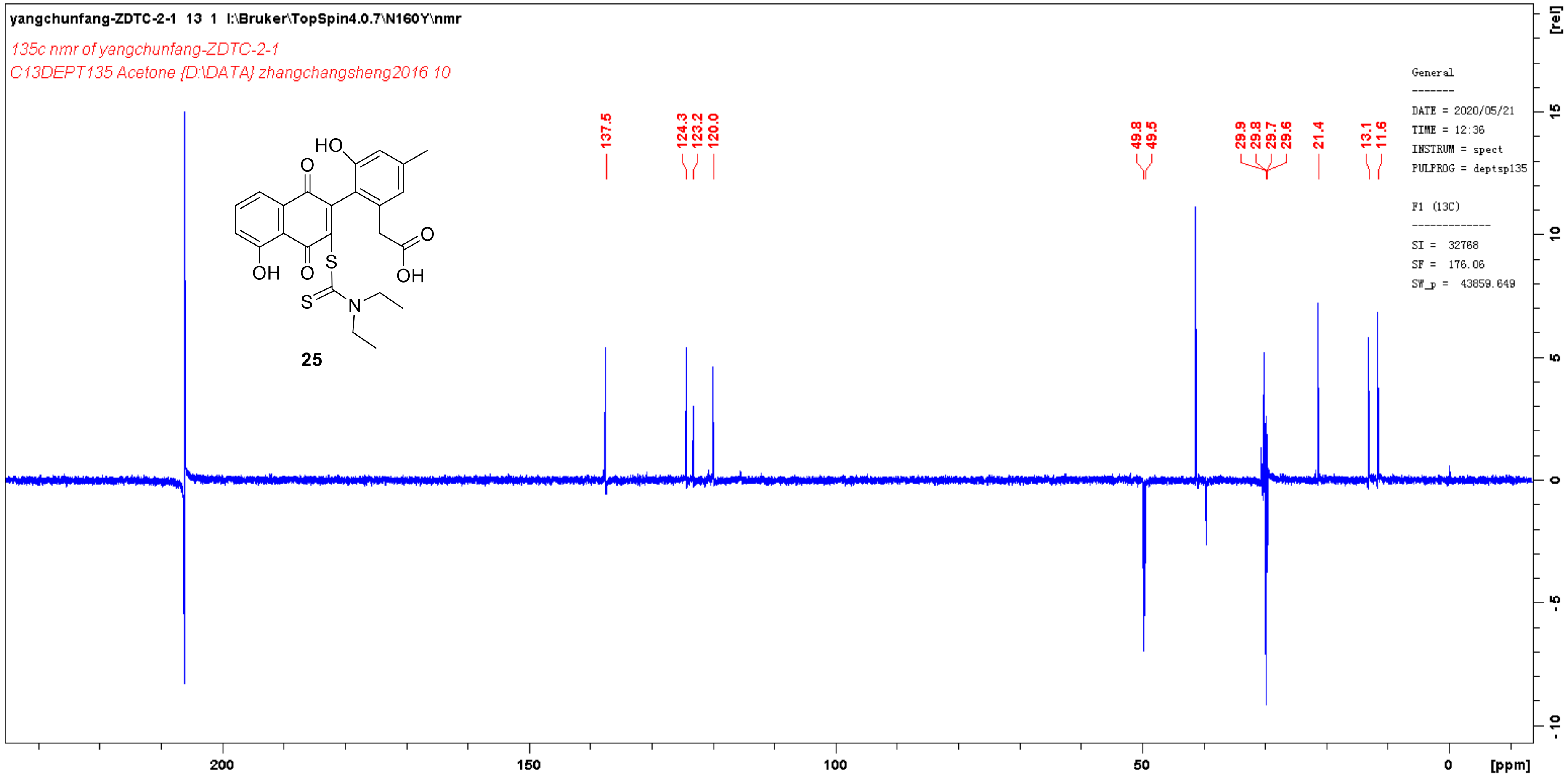

Supplementary Fig. 47. The DEPT 135 spectrum of DTC-fluostacid B (25) in acetone-d_6.

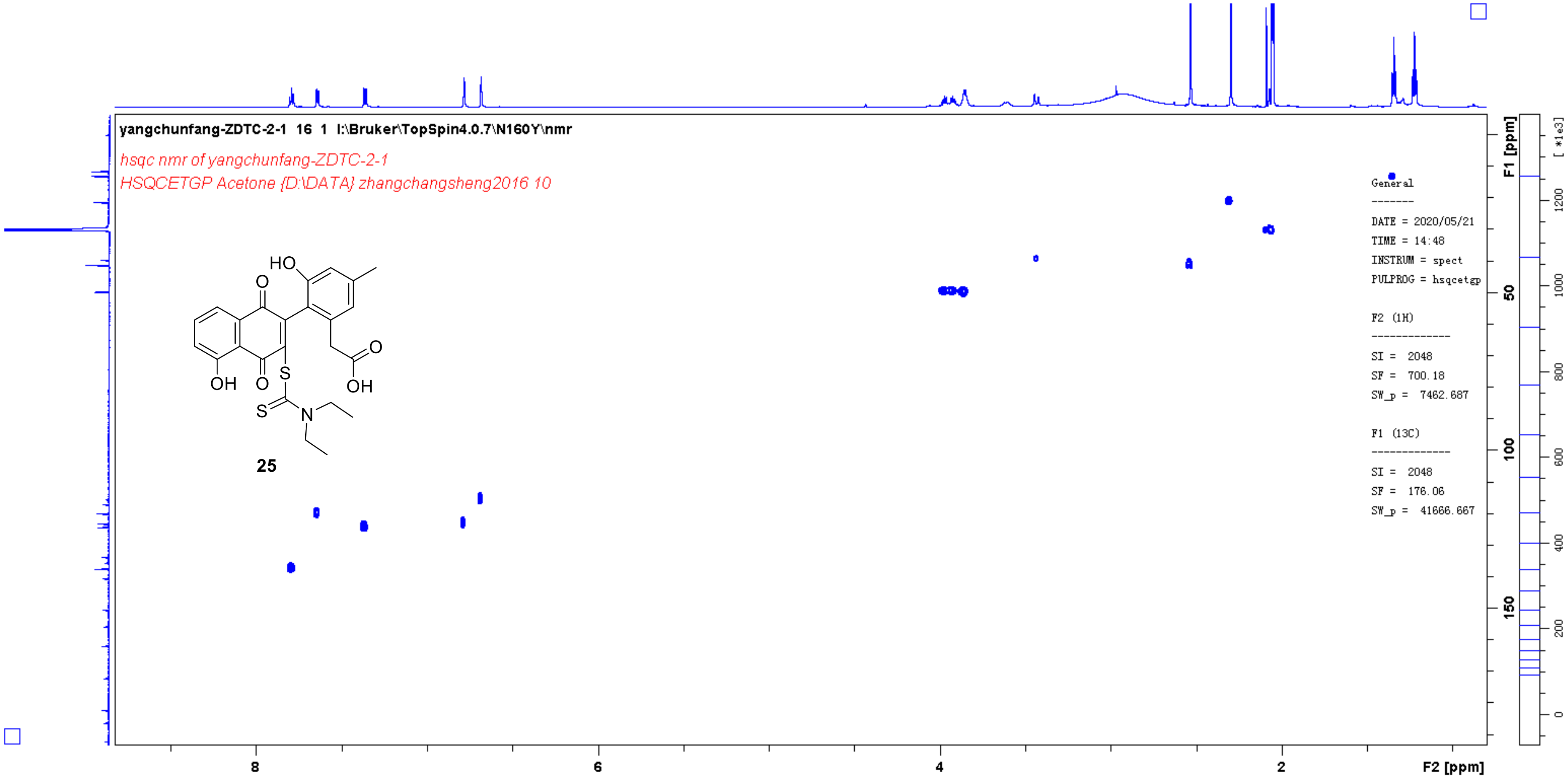

Supplementary Fig. 48. The HSQC spectrum of DTC-fluostacid B (25) in acetone-d_6.

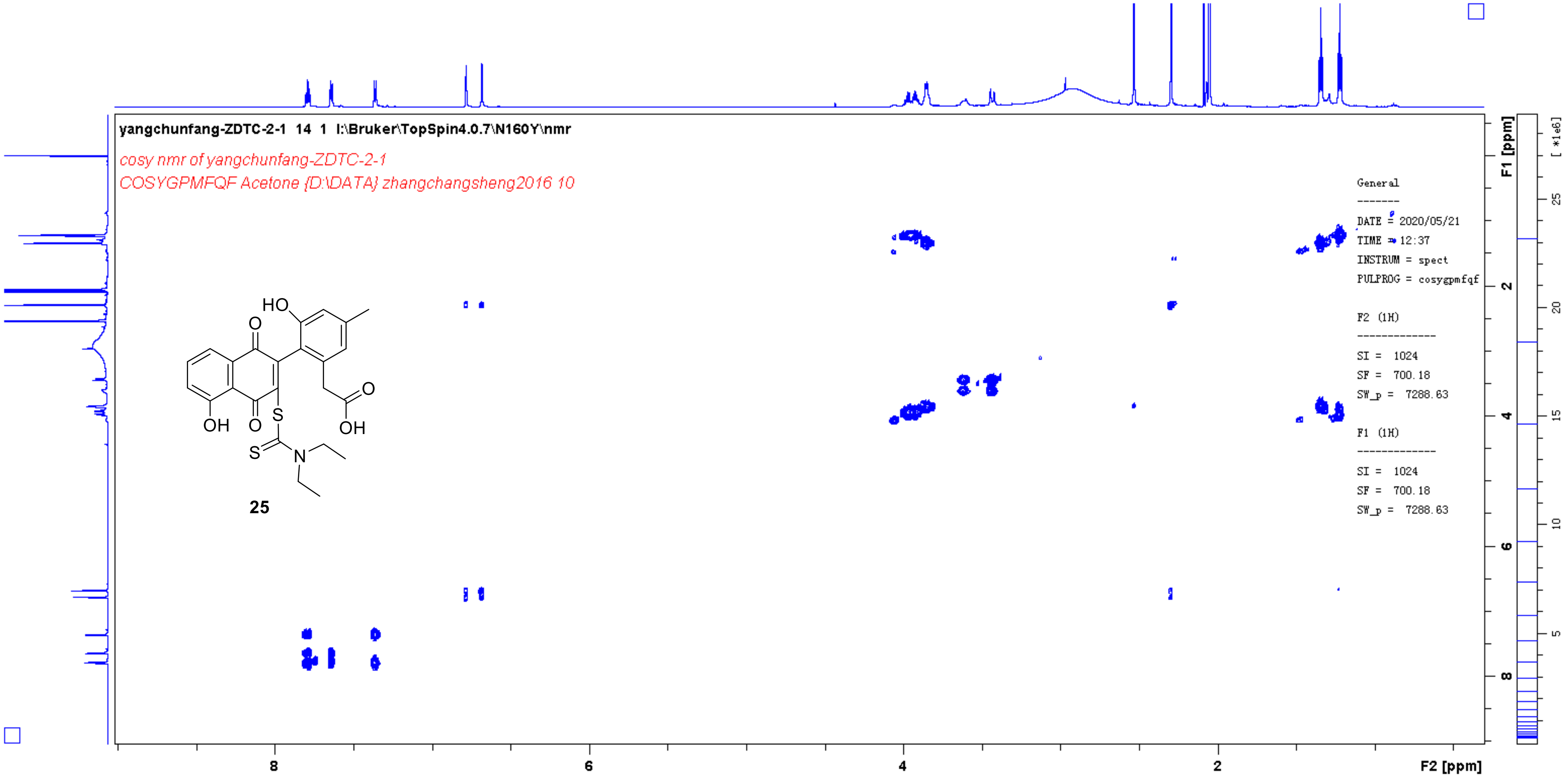

Supplementary Fig. 49. The COSY spectrum of DTC-fluostacid B (25) in acetone-d_6.

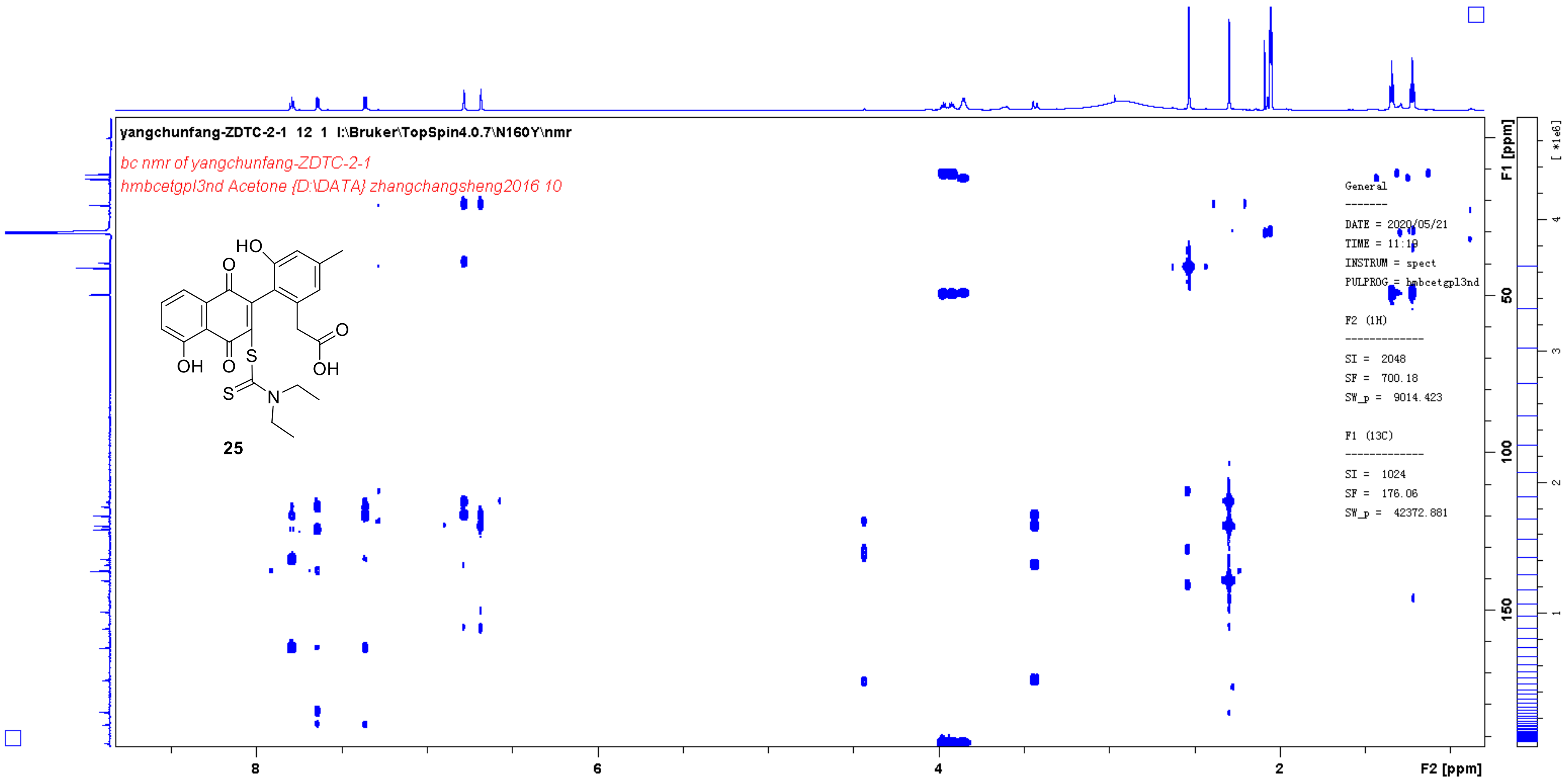

Supplementary Fig. 50. The HMBC spectrum of DTC-fluostacid B (25) in acetone-d_6.

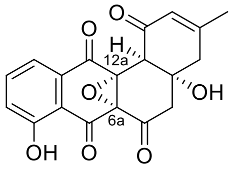

a

22

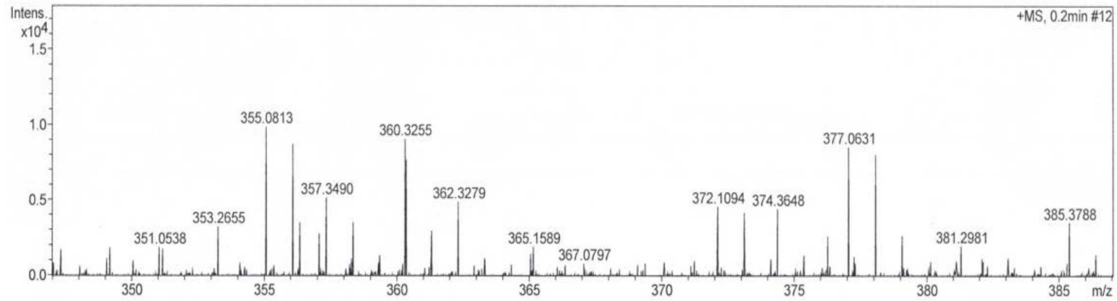

Meas. m/z	#	Ion Formula	Score	m/z	err [ppm]	err [mDa]	mSigma	rdb	e⁻ Conf	N-Rule
355.0813	1	C19H15O7	100.00	355.0812	0.1	0.0	359.8	12.5	even	ok
377.0631	1	C19H14NaO7	100.00	377.0632	0.3	0.1	392.4	12.5	even	ok

b

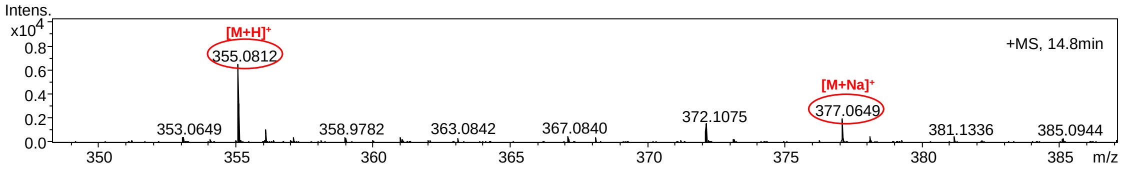

Supplementary Fig. 51. The HRESIMS spectrum of epoxy-prejadomycin (22). (**a**) The HRESIMS spectrum of **22**. (**b**) LC-HRESIMS analysis of compound **22**.

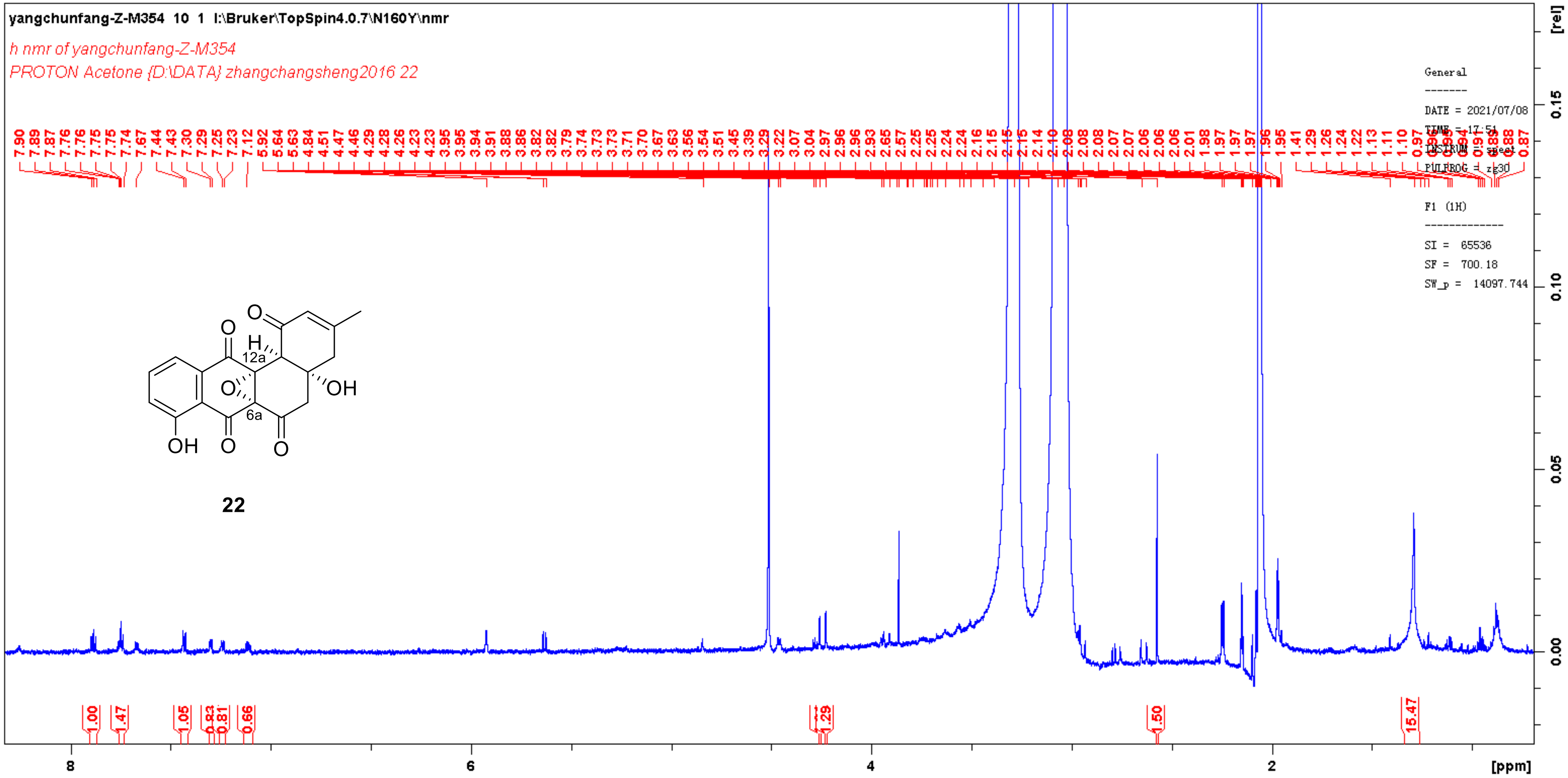

Supplementary Fig. 52. The ^{1}H NMR (700 MHz) spectrum of epoxy-prejadomycin (22) in acetone-d_6.

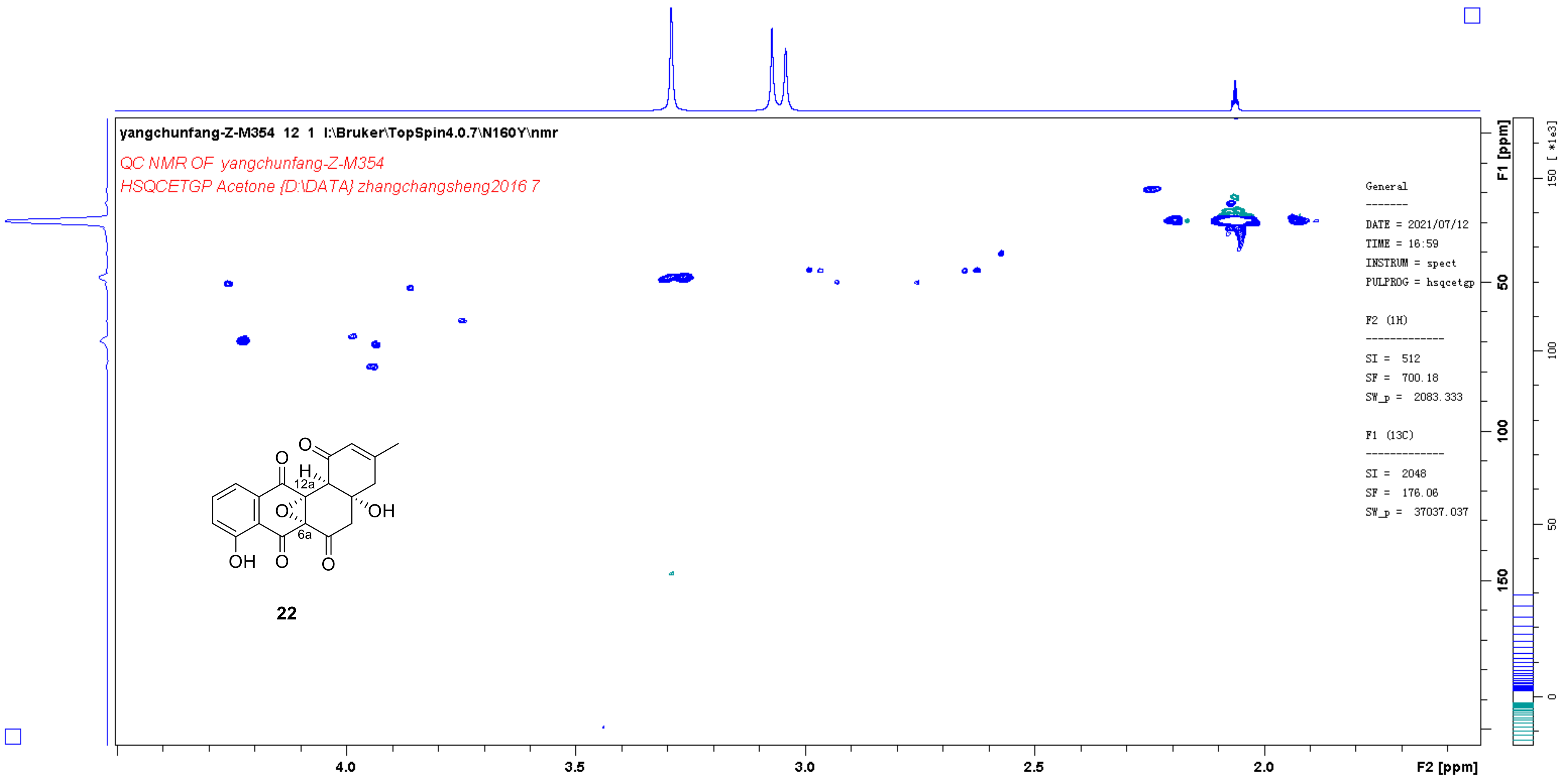

Supplementary Fig. 53. The HSQC spectrum of epoxy-prejadomycin (22) in acetone-d_6.

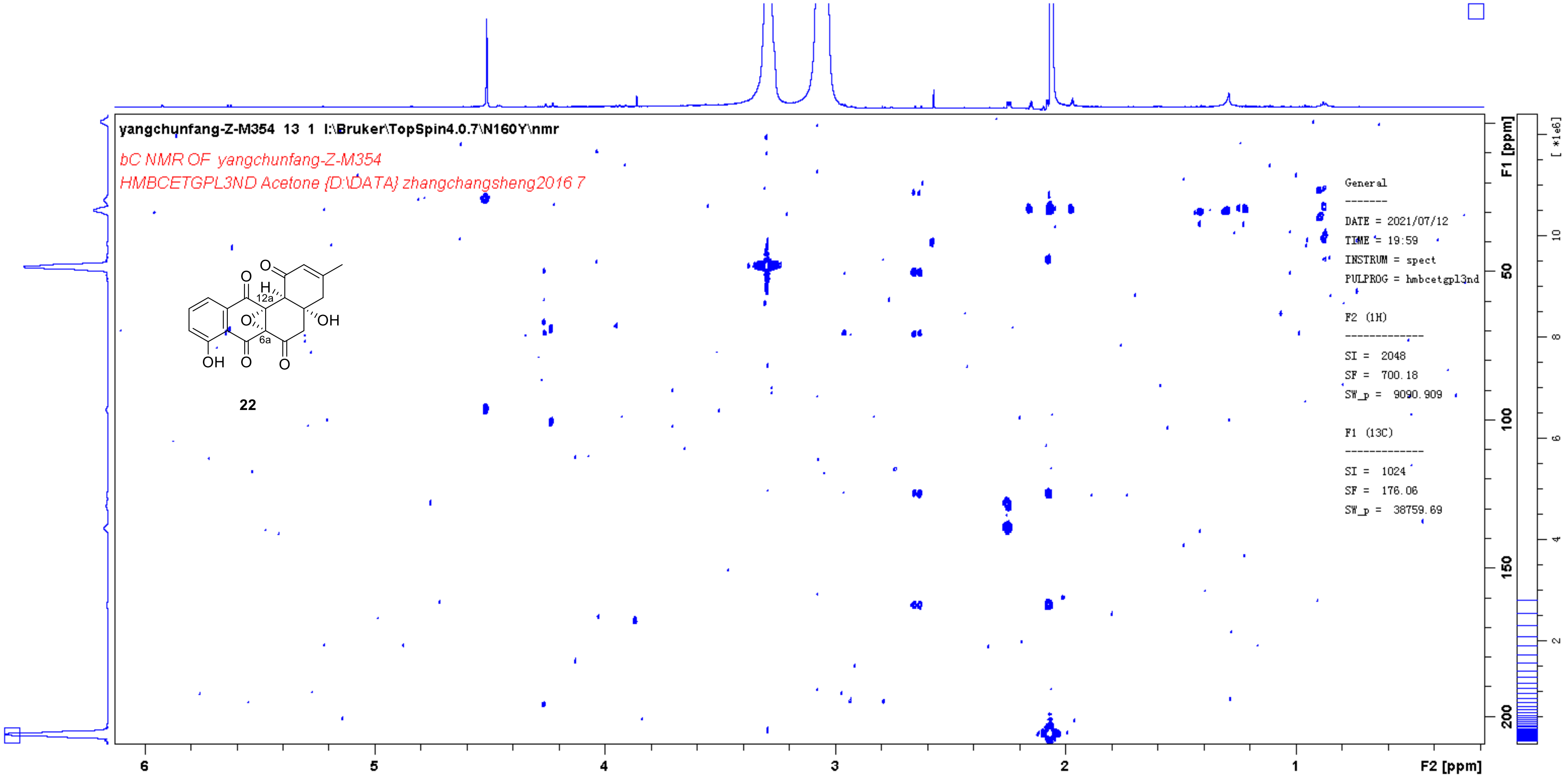

Supplementary Fig. 54. The HMBC spectrum of epoxy-prejadomycin (22) in acetone-d_6.

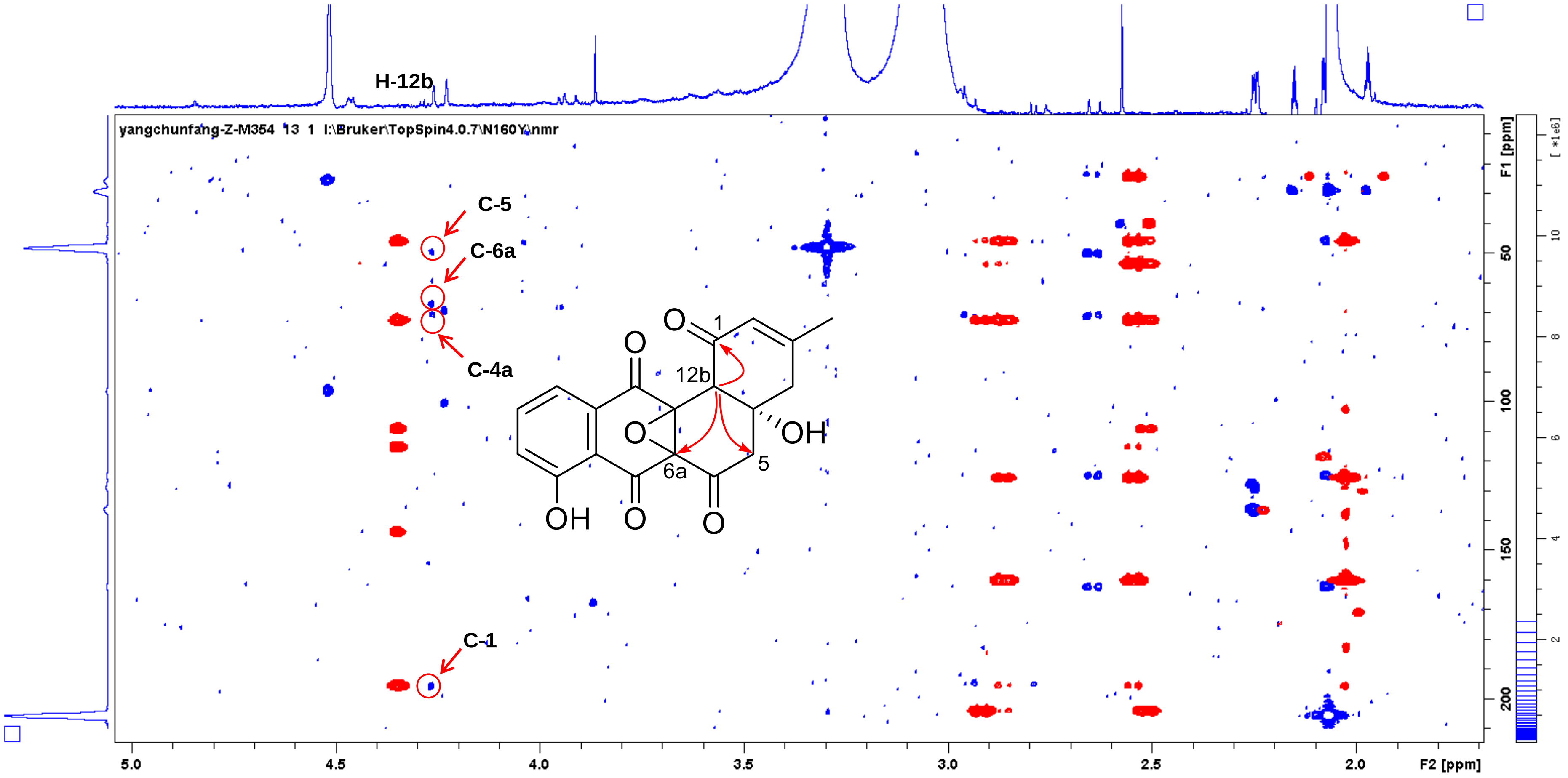

Red: The HMBC spectrum of **19**.

Blue: The HMBC spectrum of **22**.

Supplementary Fig. 55. Comparison of the the HMBC spectrum of 22 and 19.

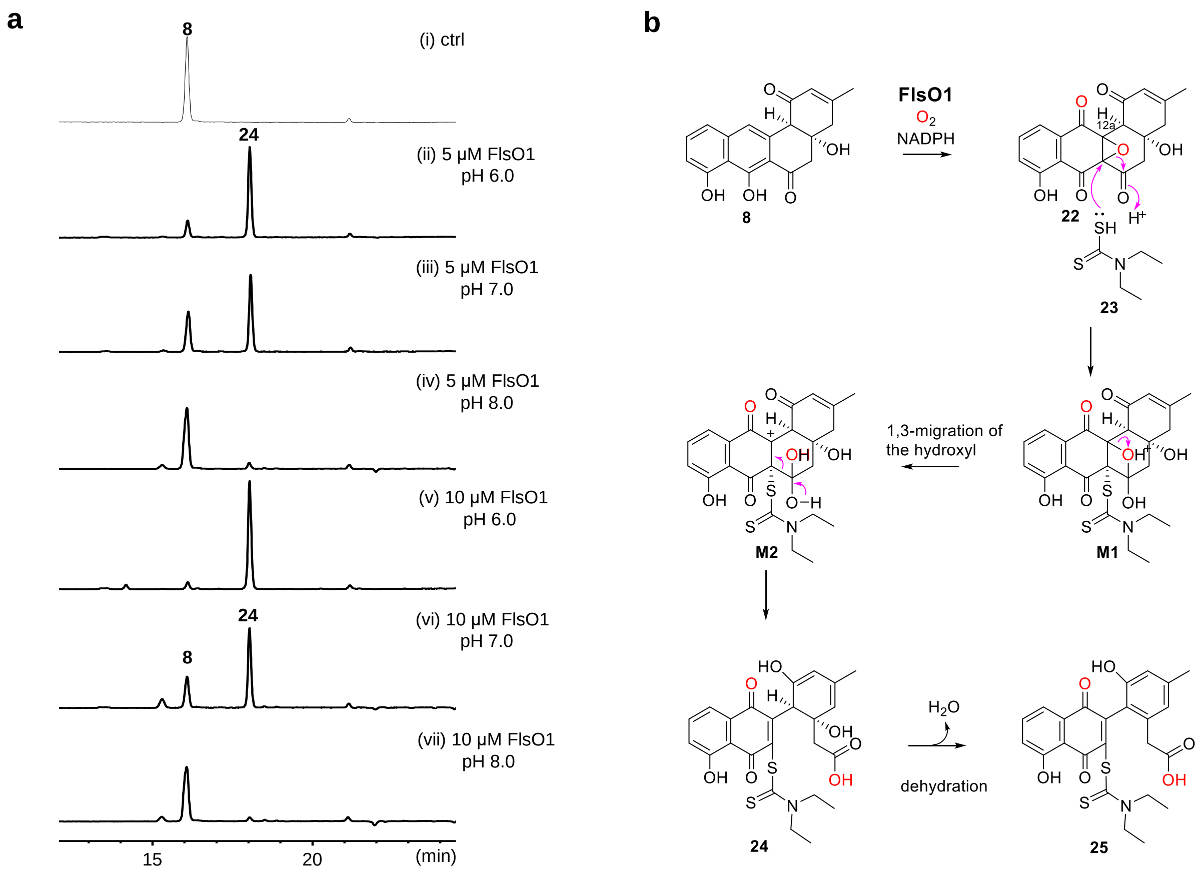

Supplementary Fig. 56. The proposed mechanism for the formation of 24. (**a**) HPLC analysis of FlsO1 enzyme assays. The assays were performed by incubation of 200 μM **8** in the presence of 2 mM NADPH and 5 mM DTC (**23**): (i) control (no enzyme); (ii) pH 6.0; (iii) pH 7.0; (iv) pH 8.0, (ii–iv) assays with 5 μM FlsO1. (v) pH 6.0; (vi) pH 7.0; (vii) pH 8.0, (v–vii) assays with 10 μM FlsO1. The reactions were performed in PBS buffers (50 mM) at 30 °C (iii-ix) for 6 min. (**b**) The proposed mechanism for the formation of **24** from DTC-trapping reaction of **22**,

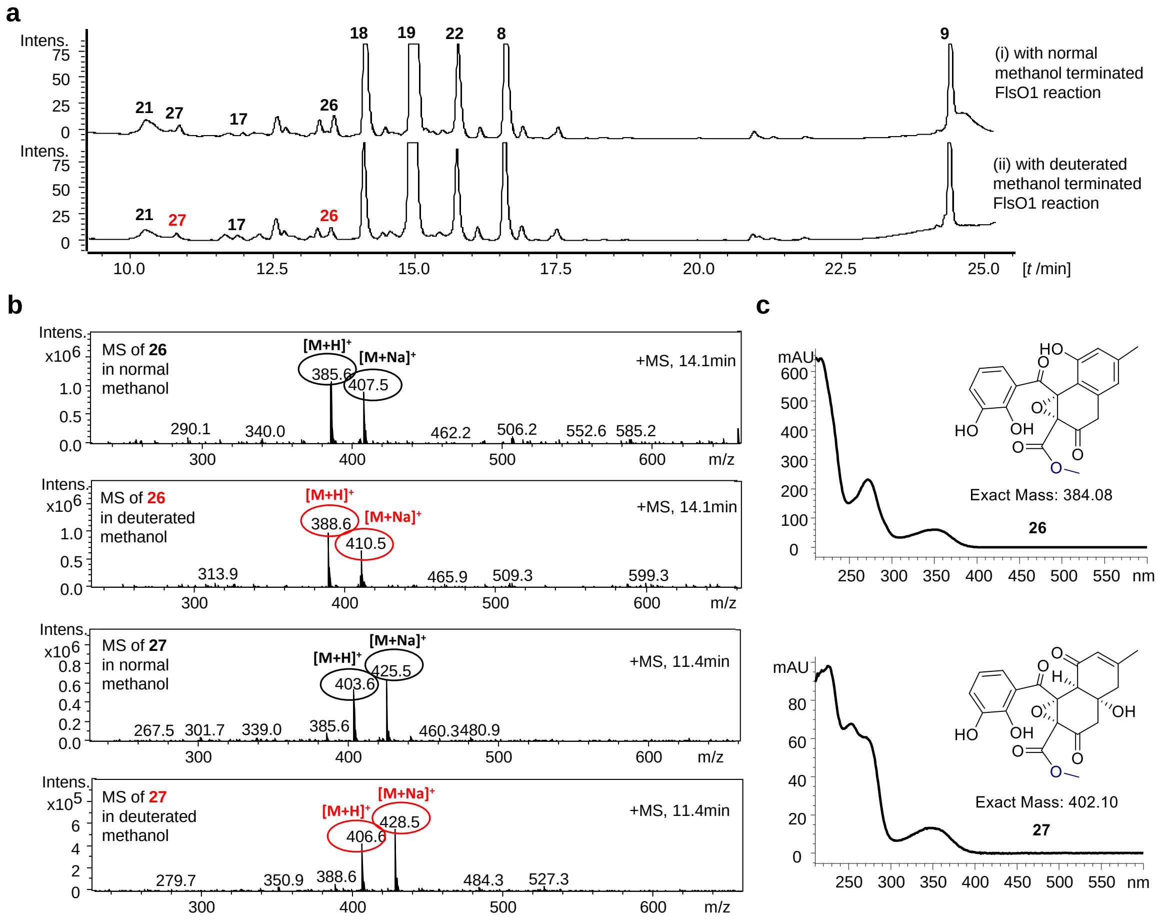

Supplementary Fig. 57. LC-MS analysis of the FlsO1 reactions terminated with normal and deuterated MeOH. (**a**) HPLC profiles of FlsO1 reactions terminated with normal and deuterated MeOH. (**b**) MS comparison of **26** and **27** with normal and deuterated methanol terminated the reaction respectively. (**c**) The UV spectrums and structures of **26** and **27**.

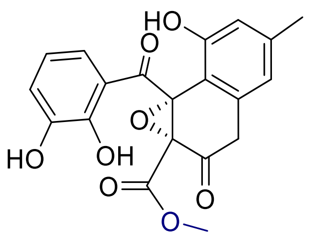

26

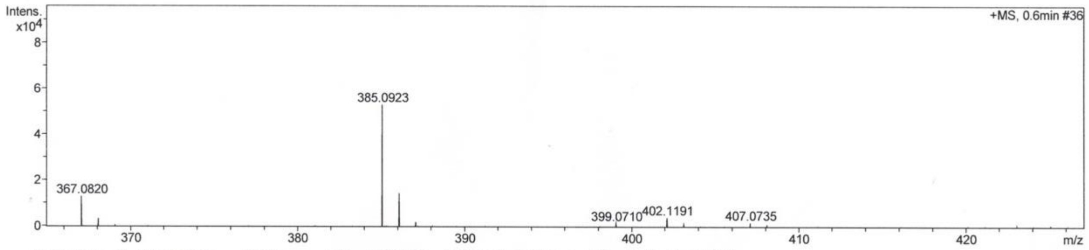

Meas. m/z	#	Ion Formula	Score	m/z	err [ppm]	err [mDa]	mSigma	rdb	e⁻ Conf	N-Rule
385.0923	1	C20H17O8	100.00	385.0918	-1.4	-0.5	26.4	12.5	even	ok
407.0735	1	C20H16NaO8	100.00	407.0737	0.7	0.3	110.8	12.5	even	ok
791.1592	1	C40H32NaO16	100.00	791.1583	1.2	0.9	53.6	24.5	even	ok

Supplementary Fig. 58. HRESIMS spectrum of epoxyfluoxanester B (26).

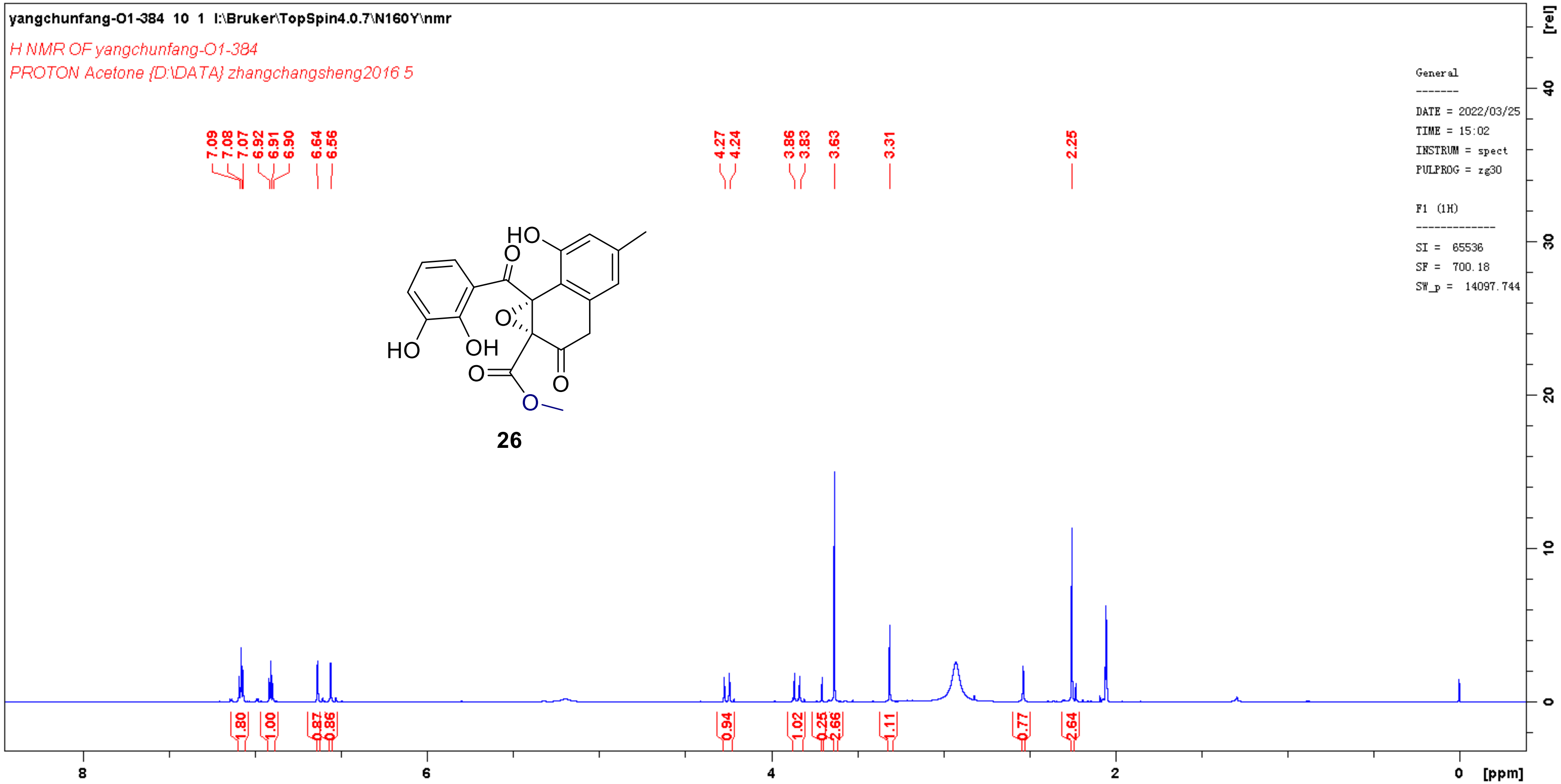

Supplementary Fig. 59. The ^{1}H NMR (700 MHz) spectrum of epoxyfluoxanester B (26) in acetone-d_6.

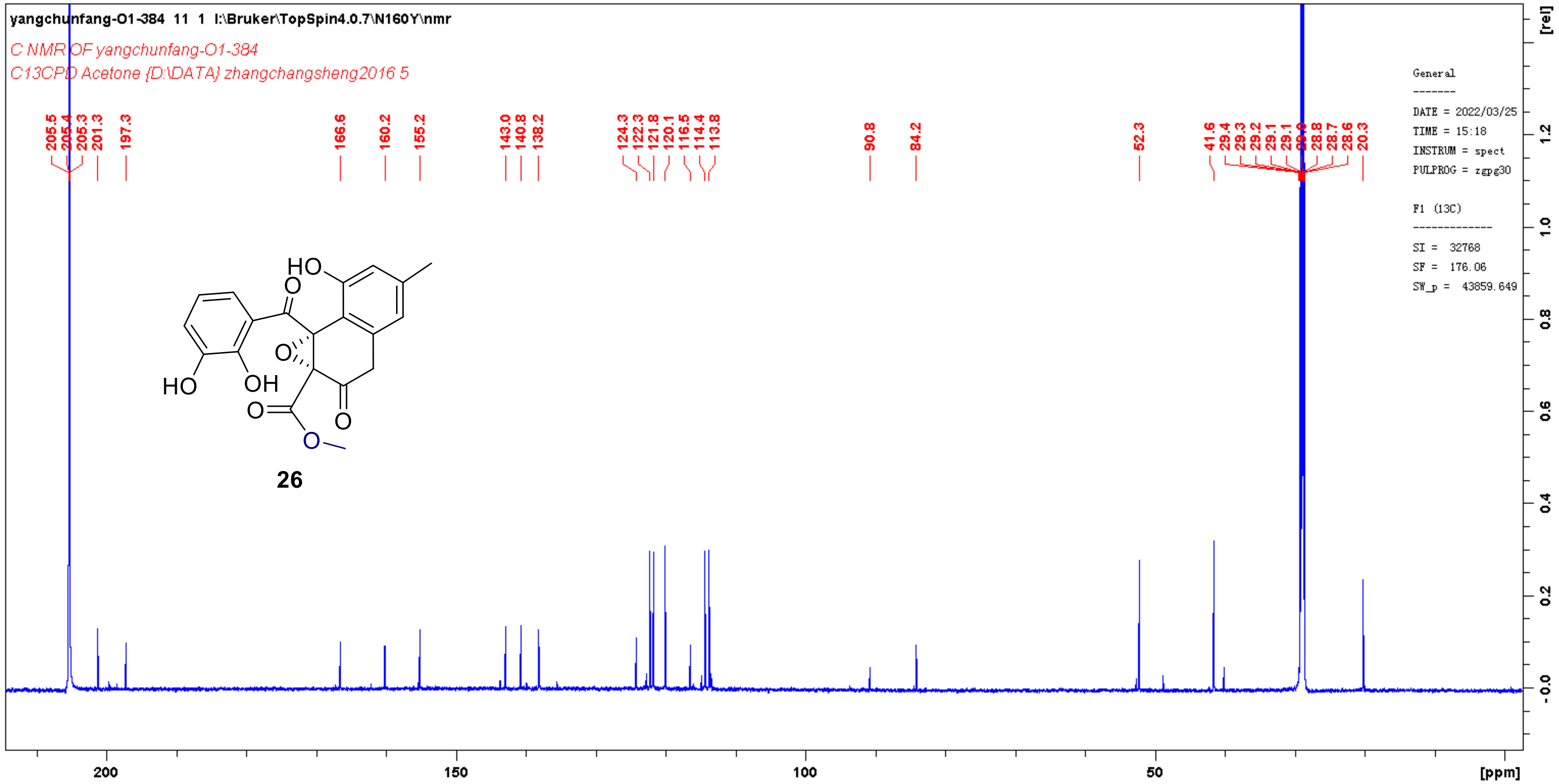

Supplementary Fig. 60. The ^{13}C (176 MHz) spectrum of epoxyfluoxanester B (26) in acetone-d_6.

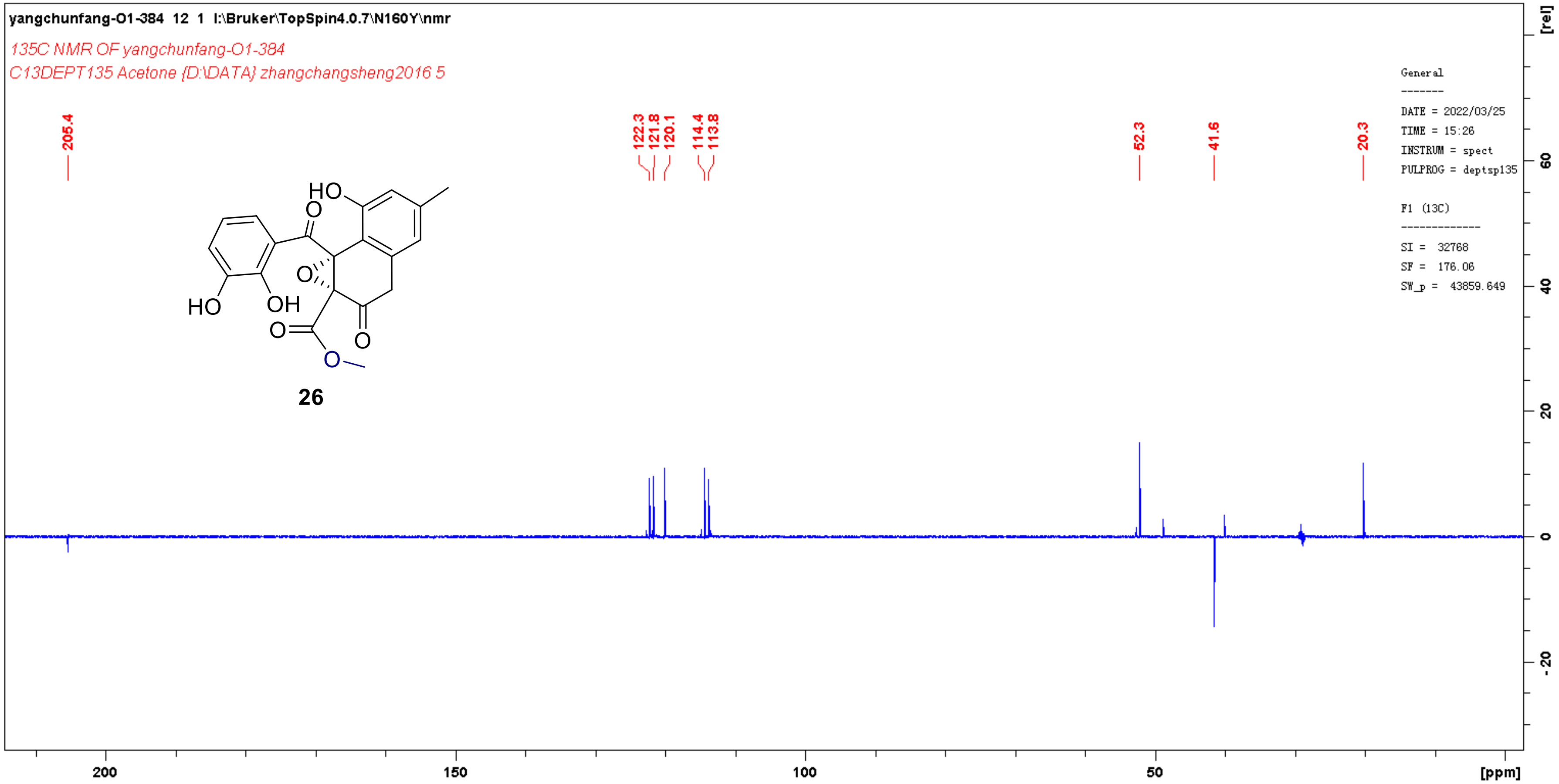

Supplementary Fig. 61. The DEPT 135 spectrum of epoxyfluoxanester B (26) in acetone-d_6.

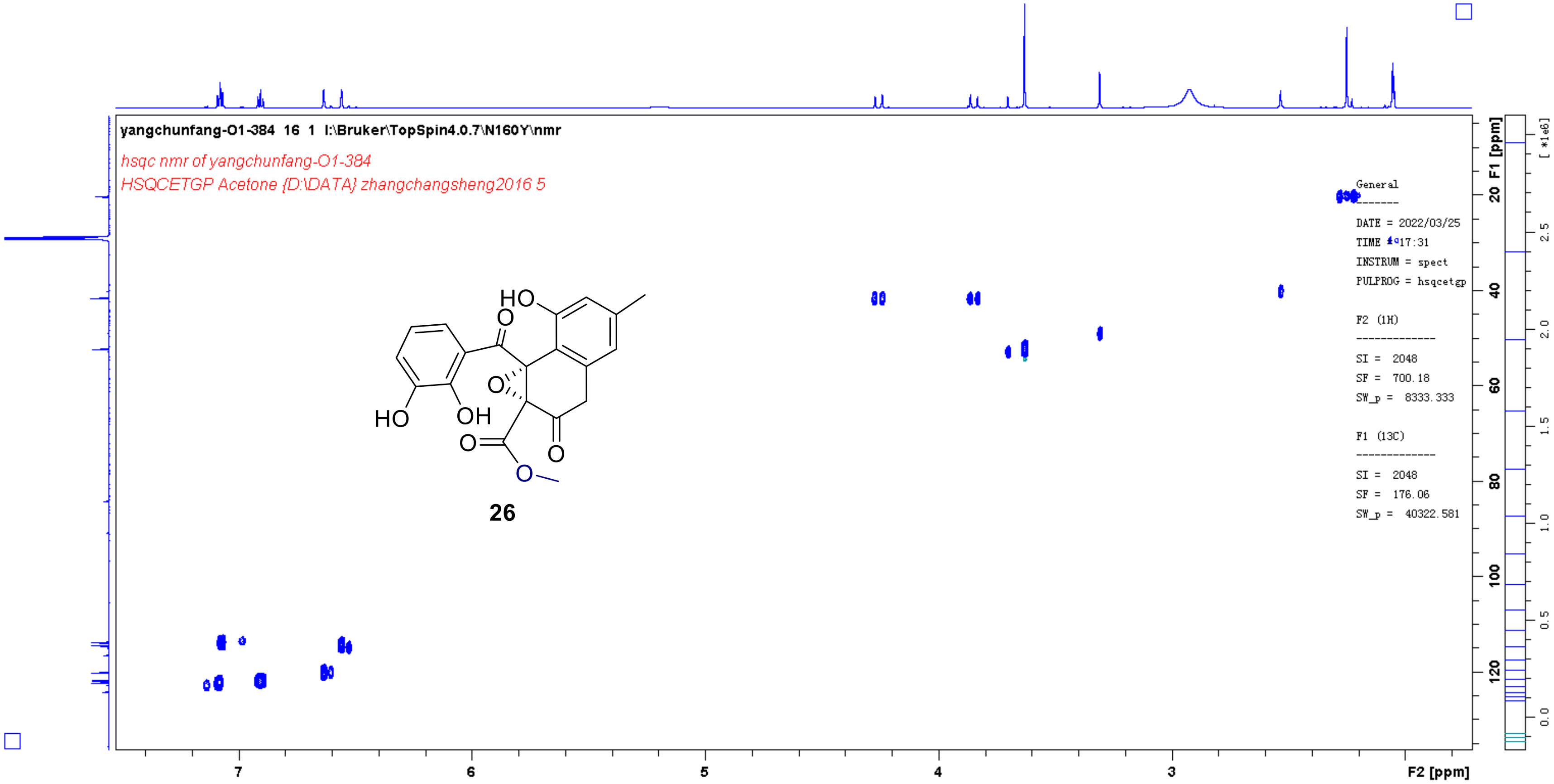

Supplementary Fig. 62. The HSQC spectrum of epoxyfluoxanester B (26) in acetone-d_6.

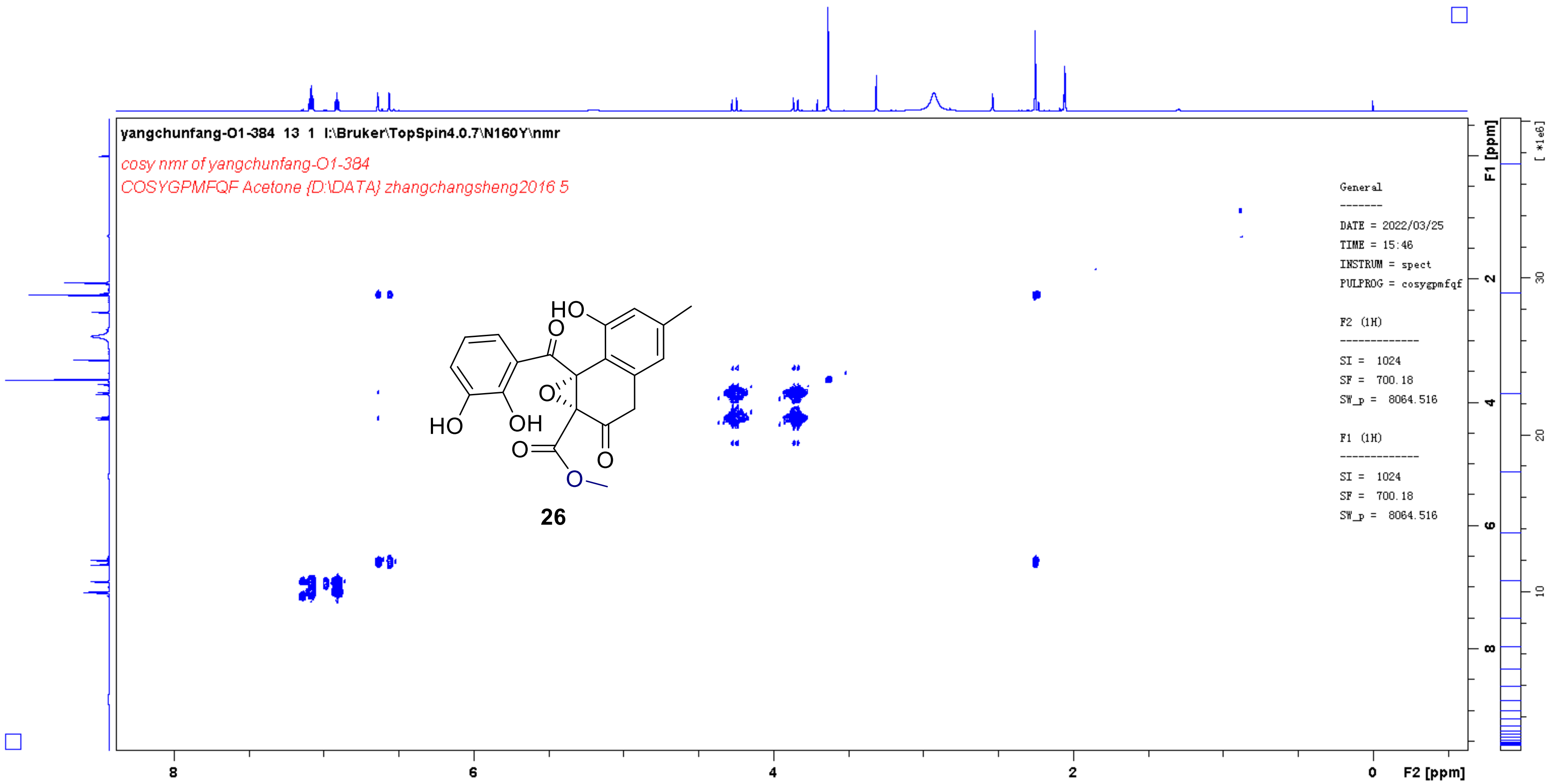

Supplementary Fig. 63. The COSY spectrum of epoxyfluoxanester B (26) in acetone-d_6.

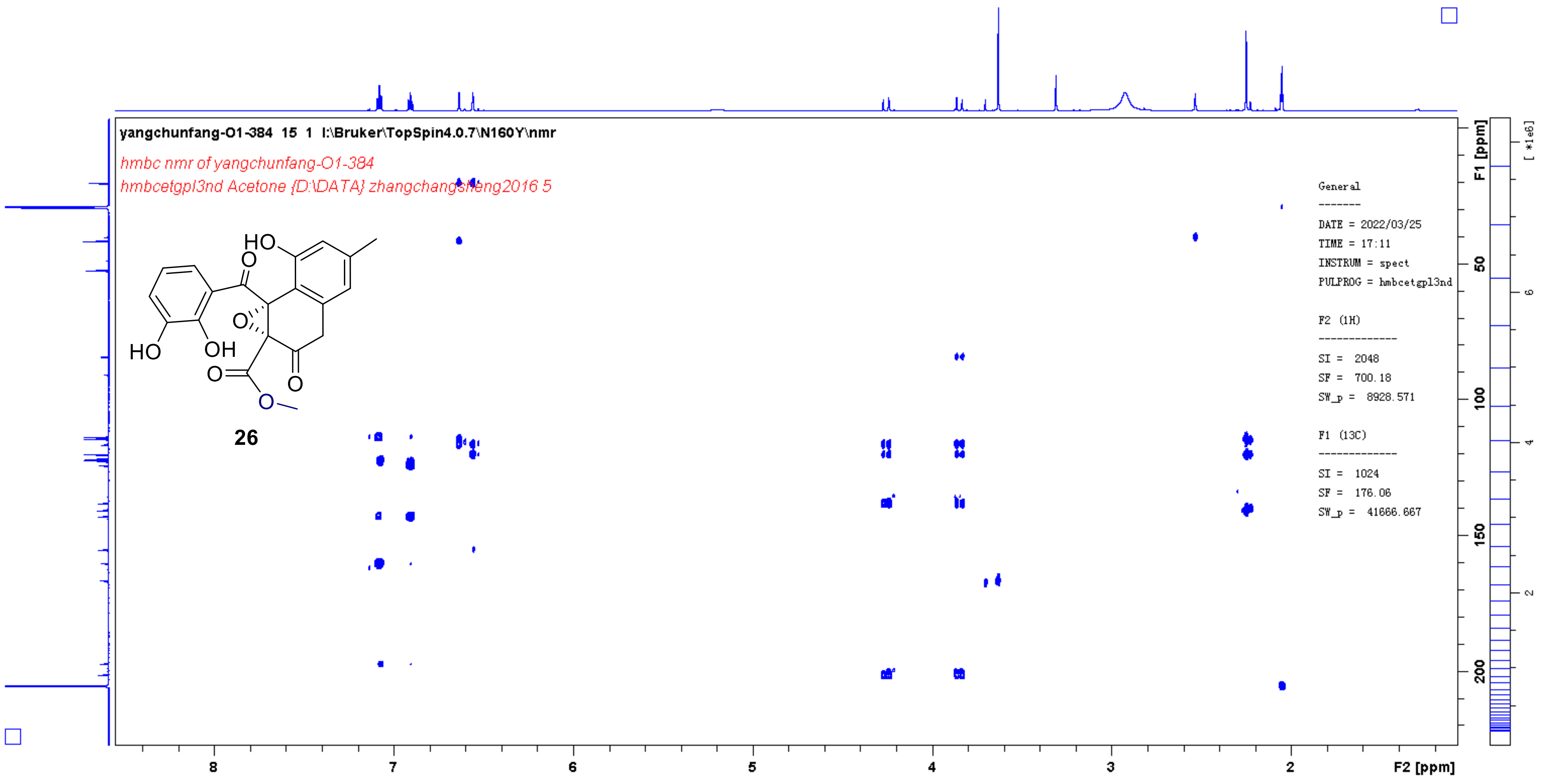

Supplementary Fig. 64. The HMBC spectrum of epoxyfluoxanester B (26) in acetone-d_6

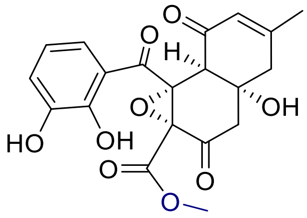

27

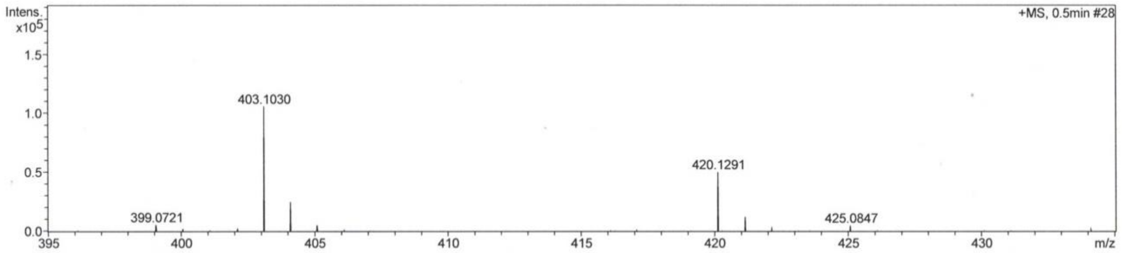

Meas. m/z	#	Ion Formula	Score	m/z	err [ppm]	err [mDa]	mSigma	rdb	e⁻ Conf	N-Rule
403.1030	1	C20H19O9	100.00	403.1024	1.6	0.6	8.1	11.5	even	ok
420.1291	1	C20H22NO9	100.00	420.1289	0.5	0.2	8.5	10.5	even	ok
425.0847	1	C20H18NaO9	100.00	425.0843	0.8	0.3	34.4	11.5	even	ok

Supplementary Fig. 65. HRESIMS spectrum of epoxyfluoxanester A (27).

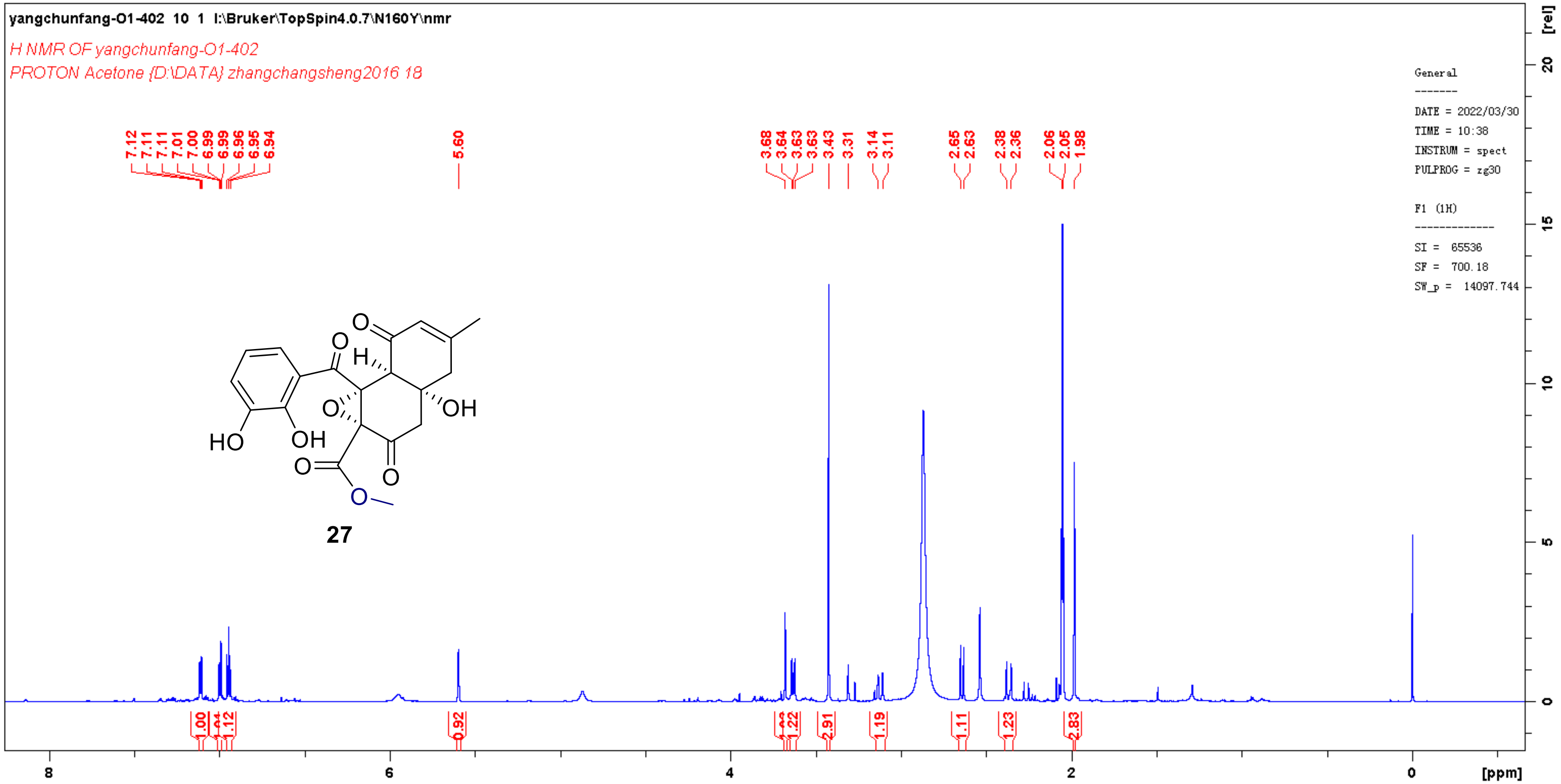

Supplementary Fig. 66. The ^{1}H NMR (700 MHz) spectrum of epoxyfluoxanester A (27) in acetone-d_6.

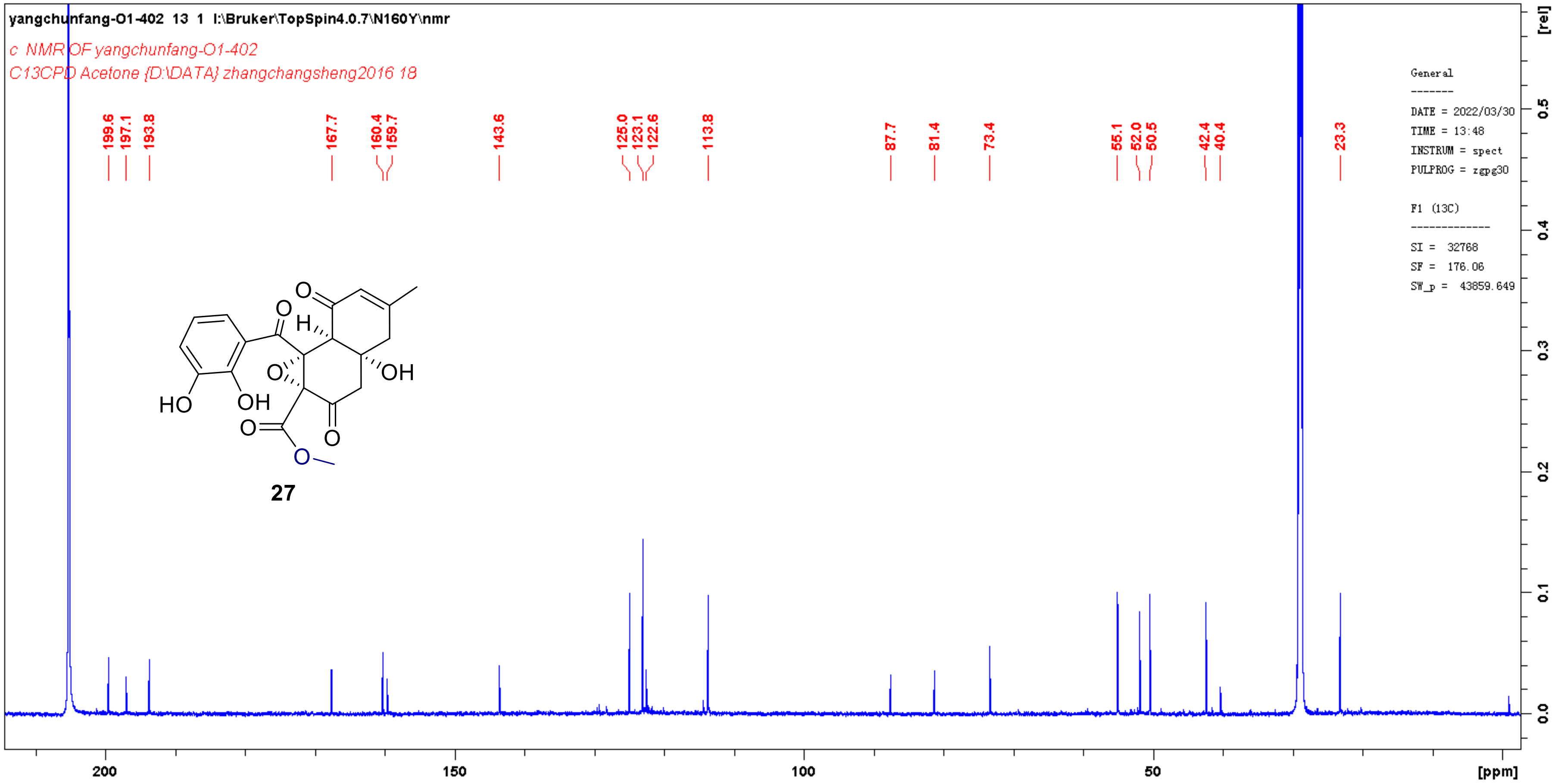

Supplementary Fig. 67. The ^{13}C (176 MHz) spectrum of epoxyfluoxanester A (27) in acetone-d_6.

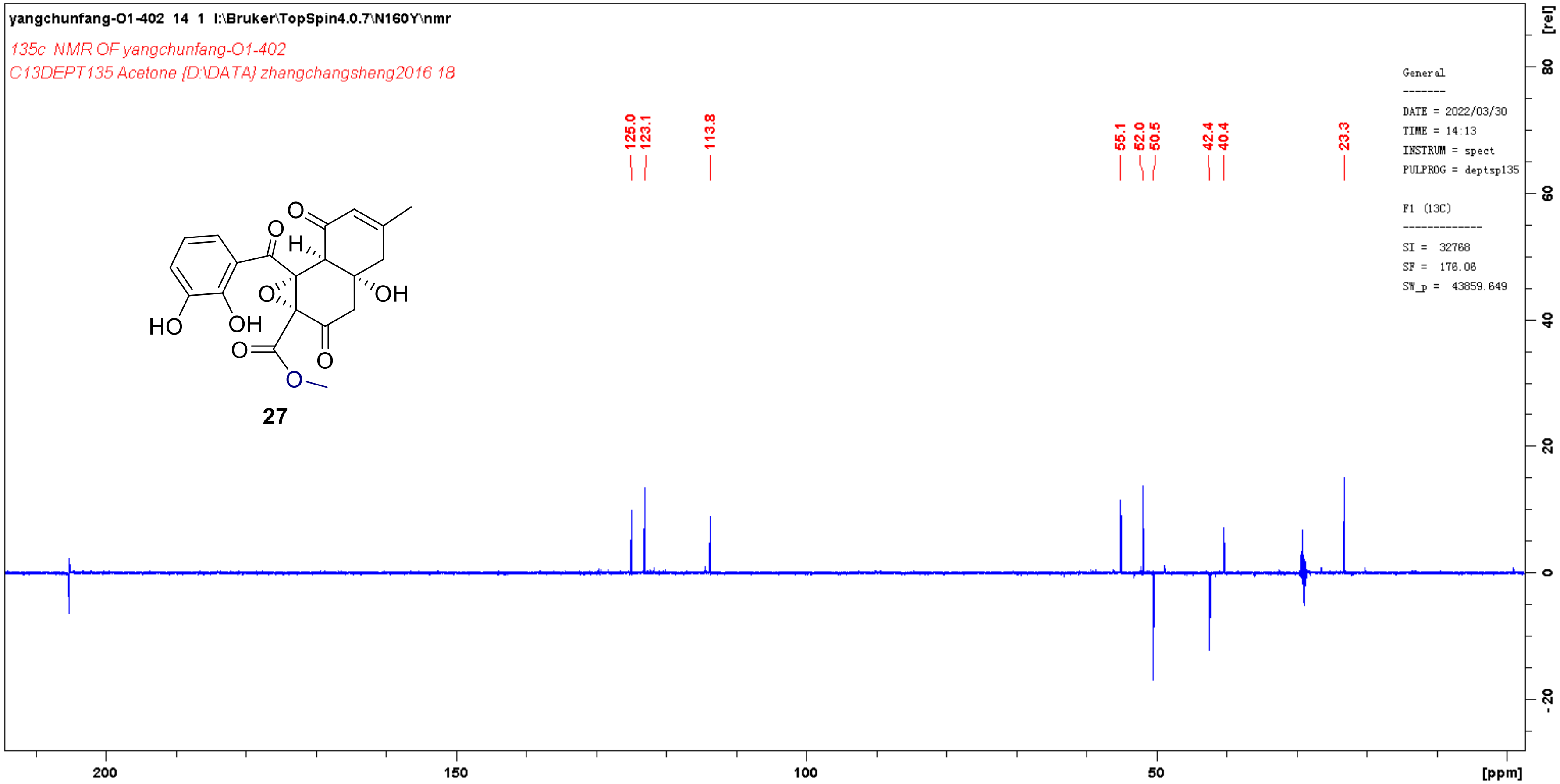

Supplementary Fig. 68. The DEPT 135 spectrum of epoxyfluoxanester A (27) in acetone-d_6.

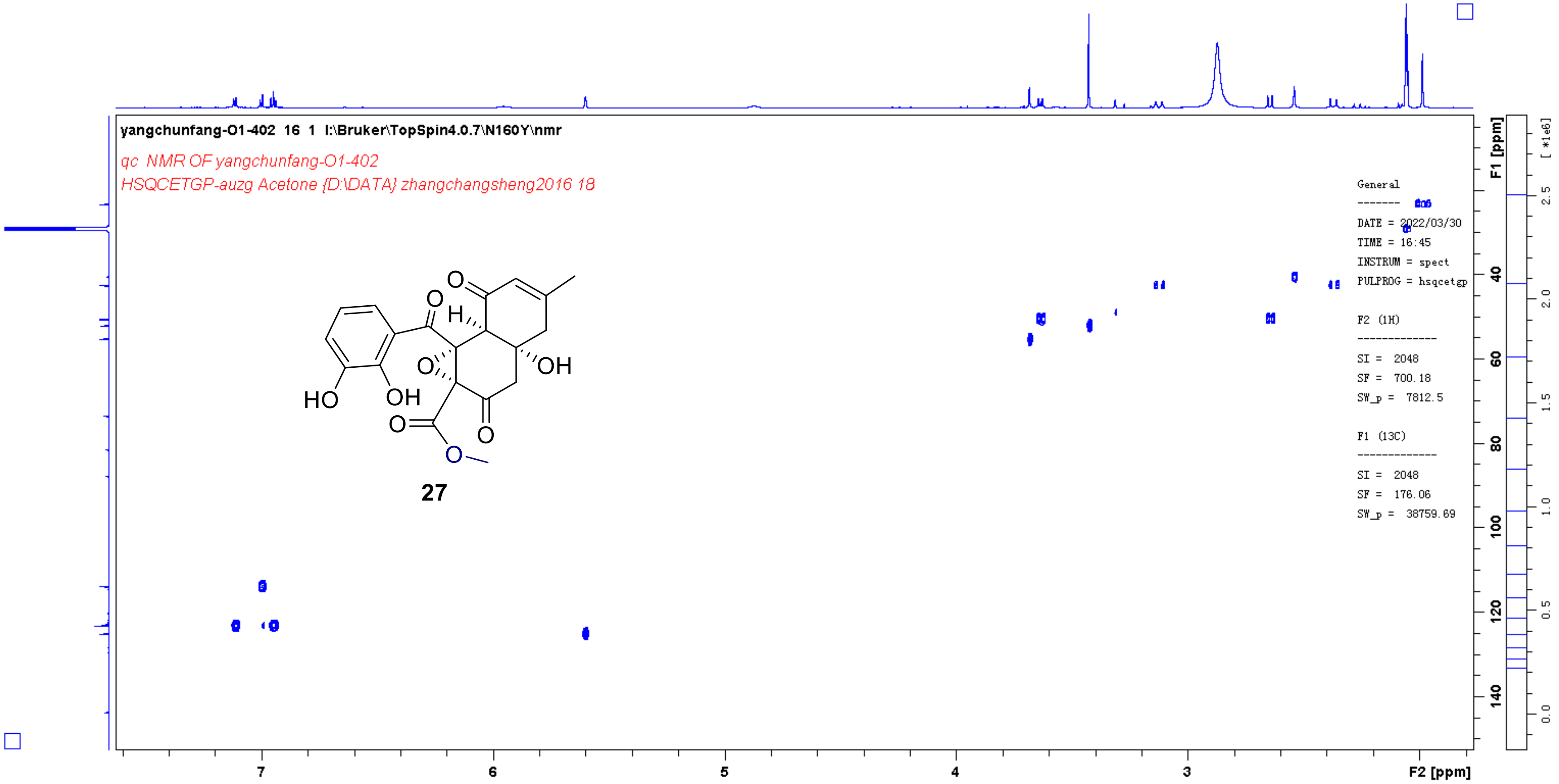

Supplementary Fig. 69. The HSQC spectrum of epoxyfluoxanester A (27) in acetone-d_6.

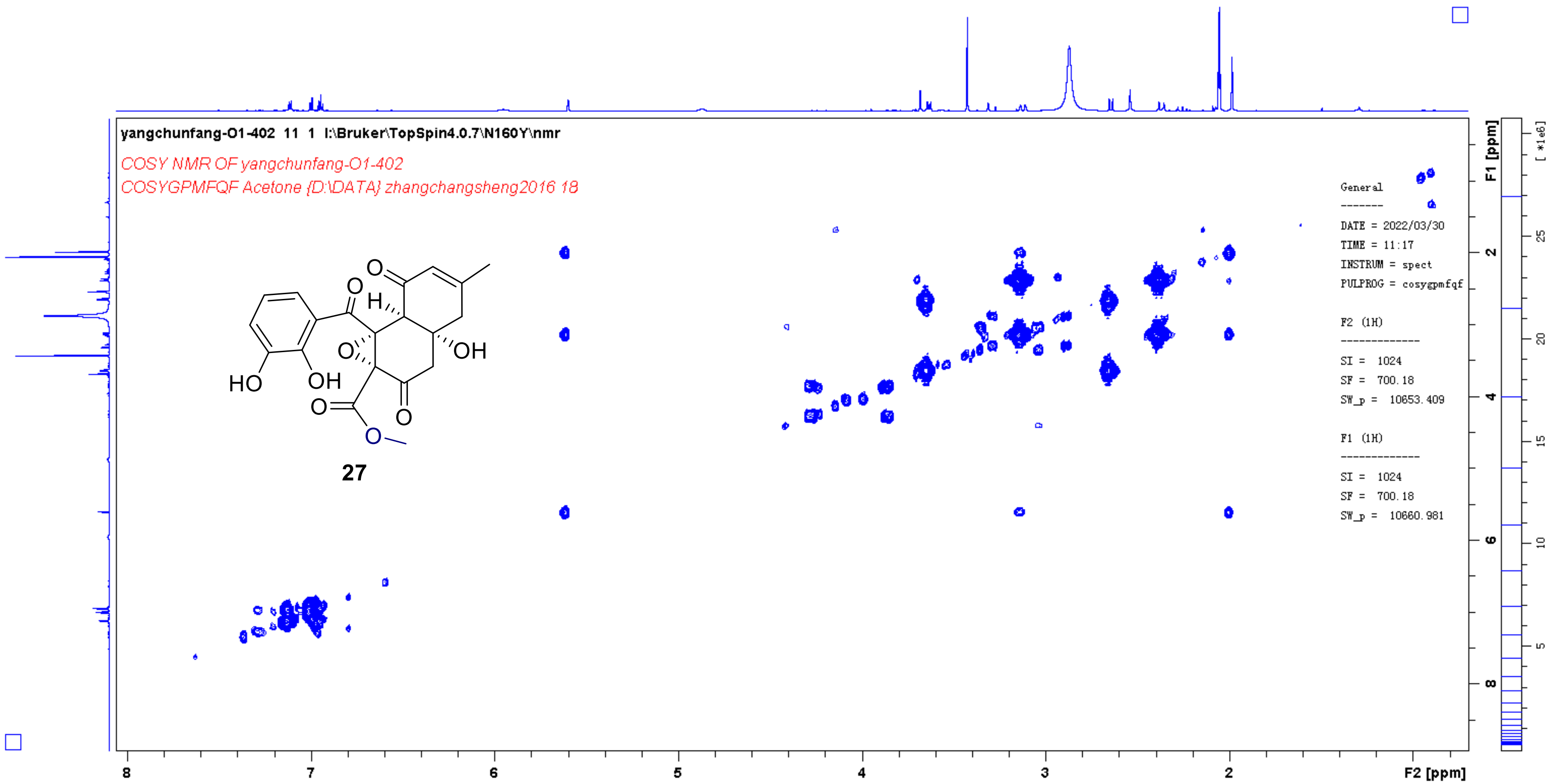

Supplementary Fig. 70. The COSY spectrum of epoxyfluoxanester A (27) in acetone-d_6.

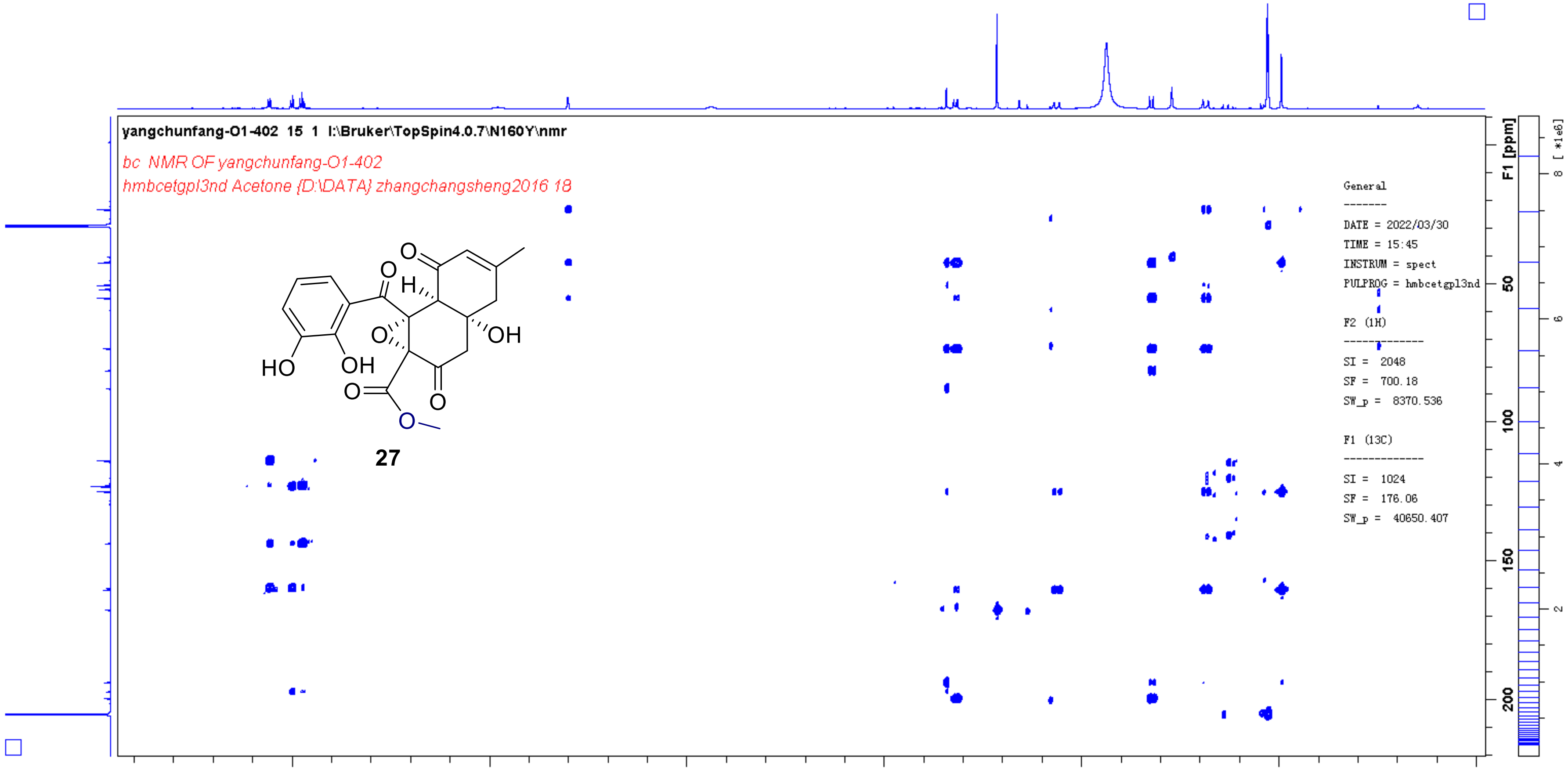

Supplementary Fig. 71. The HMBC spectrum of epoxyfluoxanester A (27) in acetone-d_6

a

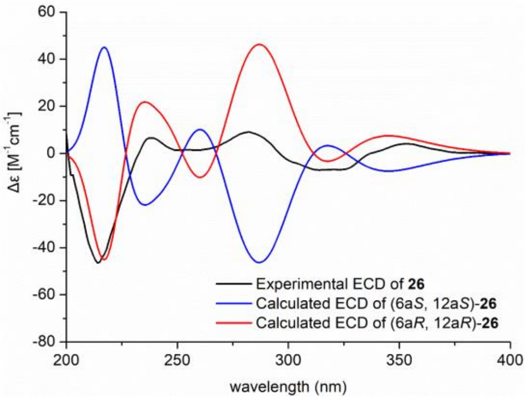

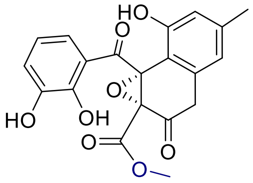

26

b

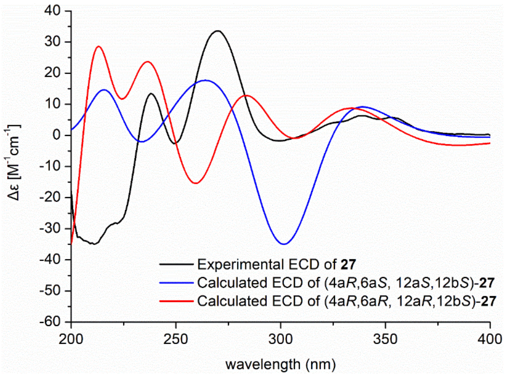

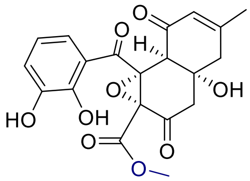

27

Supplementary Fig. 72. Comparison of the experimental and calculated ECD spectra of 26 and 27. (a) 26; (b) 27.

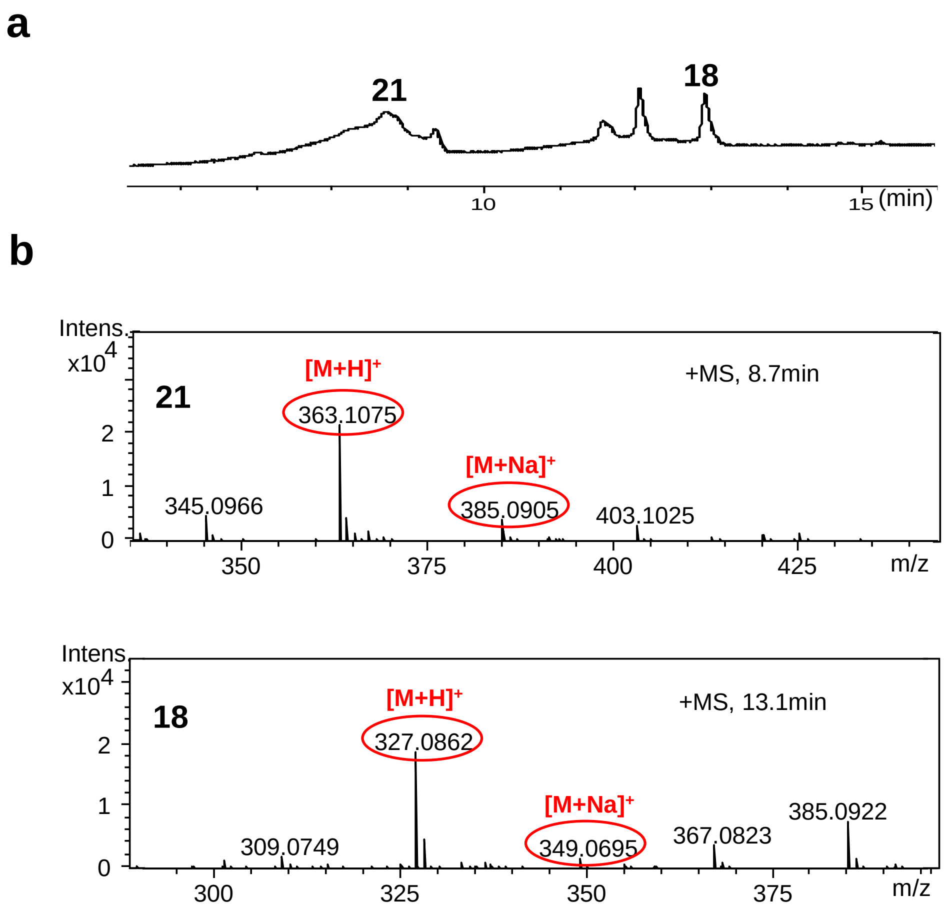

Supplementary Fig. 73. LC-HRMS analysis of the spontaneous conversion of 21 to 18. **(a)** HPLC analysis of **21** after freeze-drying. **(b)** The HRMS profiles of **21** and **18**.

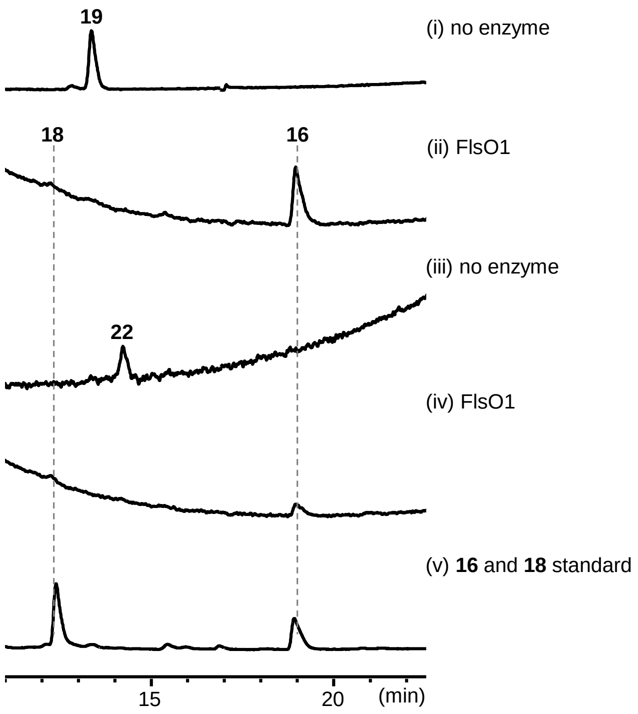

Supplementary Fig. 74. HPLC analysis of the FlsO1 reaction with 19 and 22 as the substrate. (i) control (no enzyme) + **19**; (ii) 10 μM FlsO1 + **19**; (iii) control (no enzyme) + **22**; (iv) 10 μM FlsO1 + **22**; (v) co-injection of **18** and **16** as standards. All reactions were supplemented with 2mM NADPH and conducted in 50 mM PBS buffers (pH 7.0) at 30 °C for 2h.

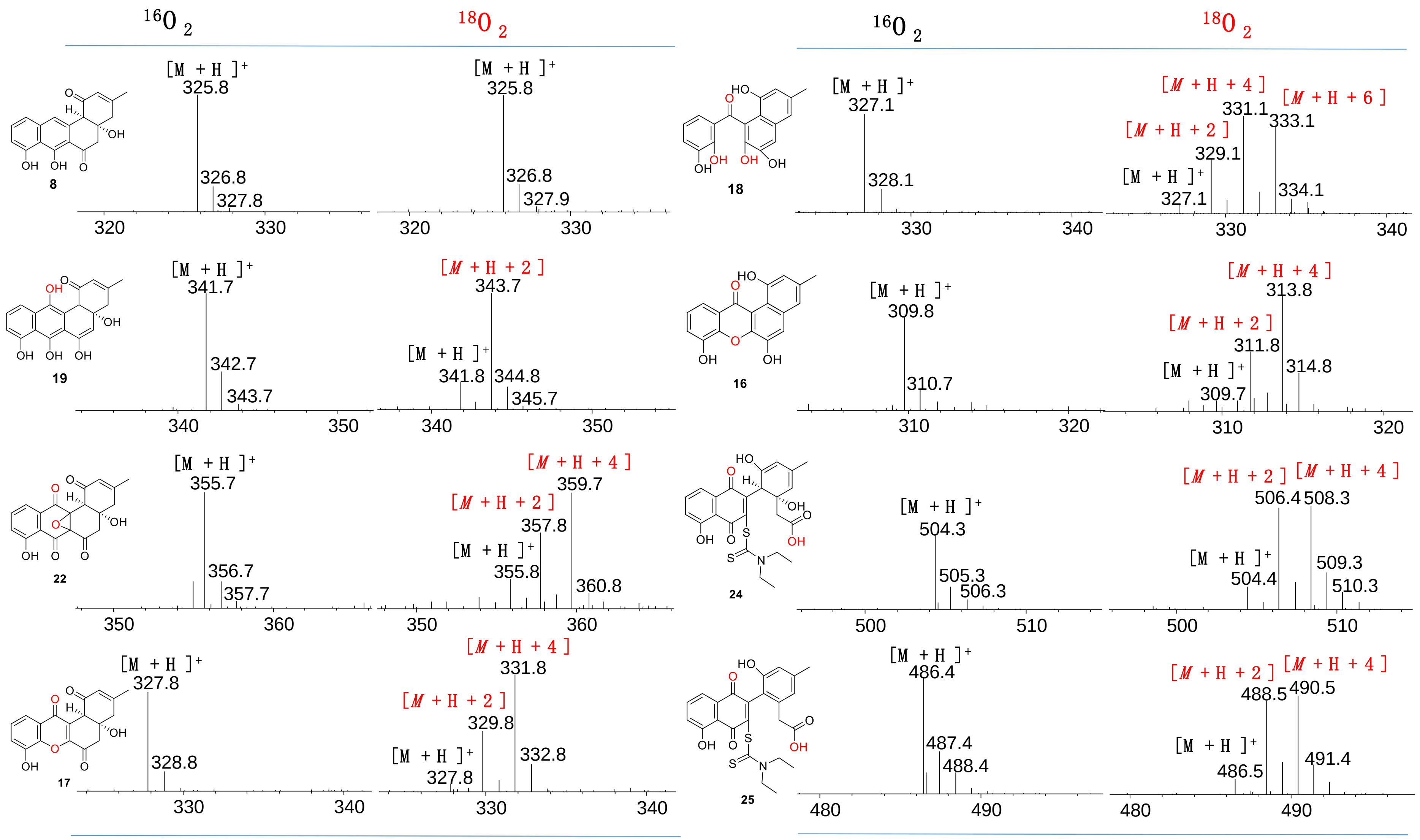

Supplementary Fig. 75. LC-MS analysis of products from FlsO1 reactions with 8 in atmospheric $^{16}O_2$ or $^{18}O_2$.

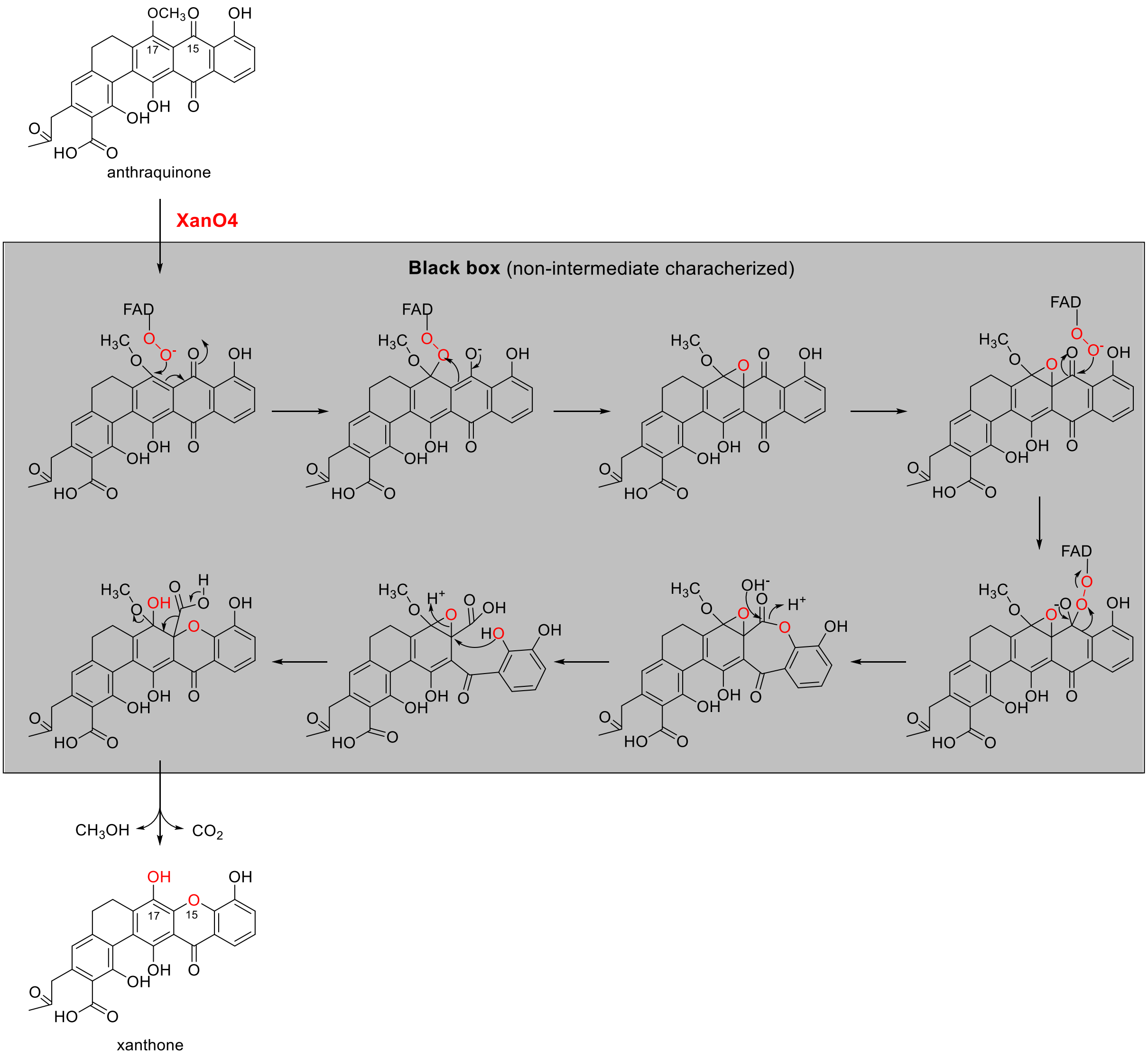

Supplementary Fig. 76. The proposed catalytic mechanism for the FPMO XanO4.

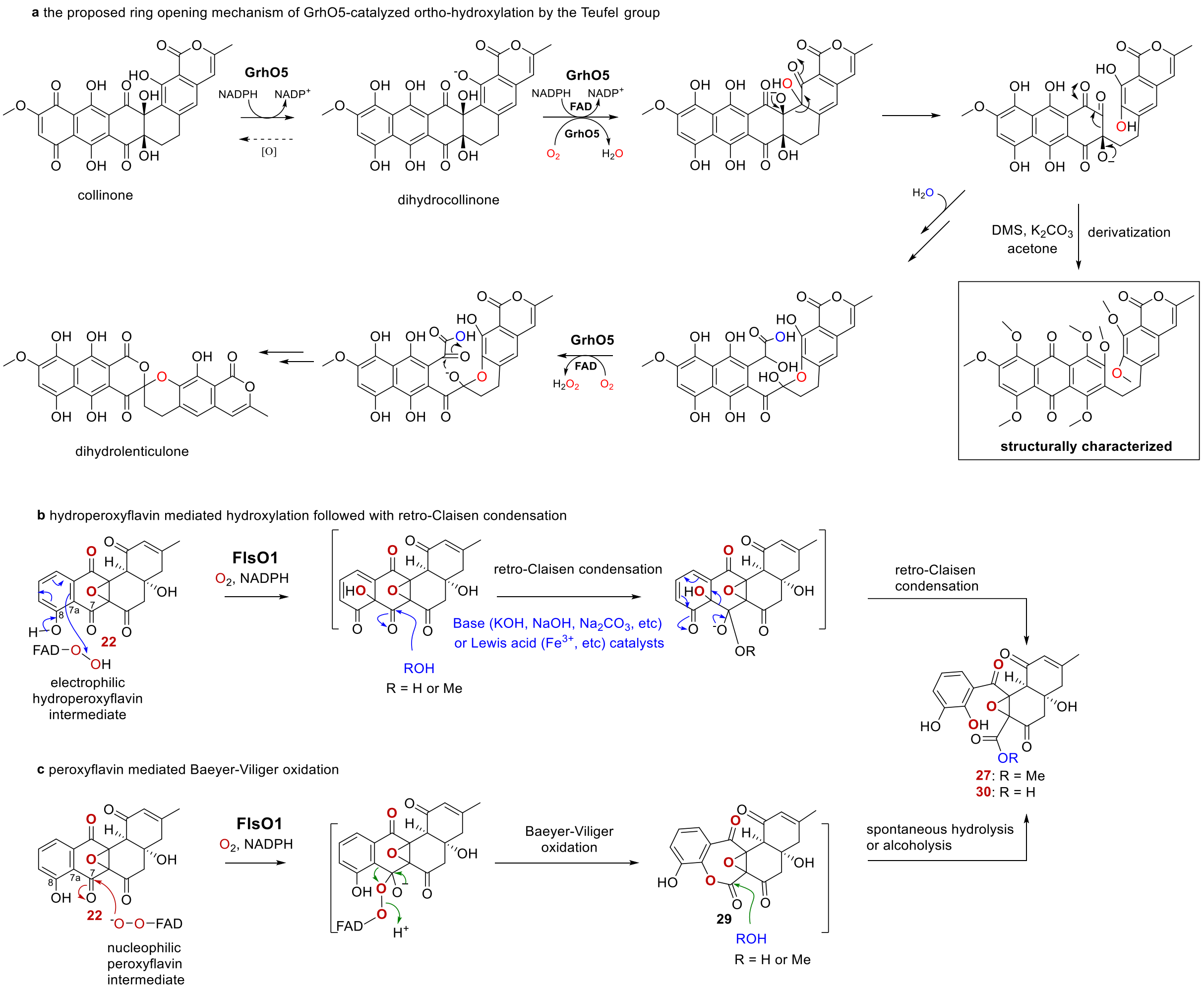

Supplementary Fig. 77. The comparison of mechanisms for FPMOs-catalyzed ring opening reaction. (**a**) The proposed ring opening mechanism of GrhO5-catalyzed *ortho*-hydroxylation by the Teufel group. (**b**) The proposed ring opening mechanism of hydroperoxyflavin mediated hydroxylation followed with retro-Claisen condensation. (**c**) The proposed ring opening mechanism of peroxyflavin mediated Baeyer-Viliger oxidation.

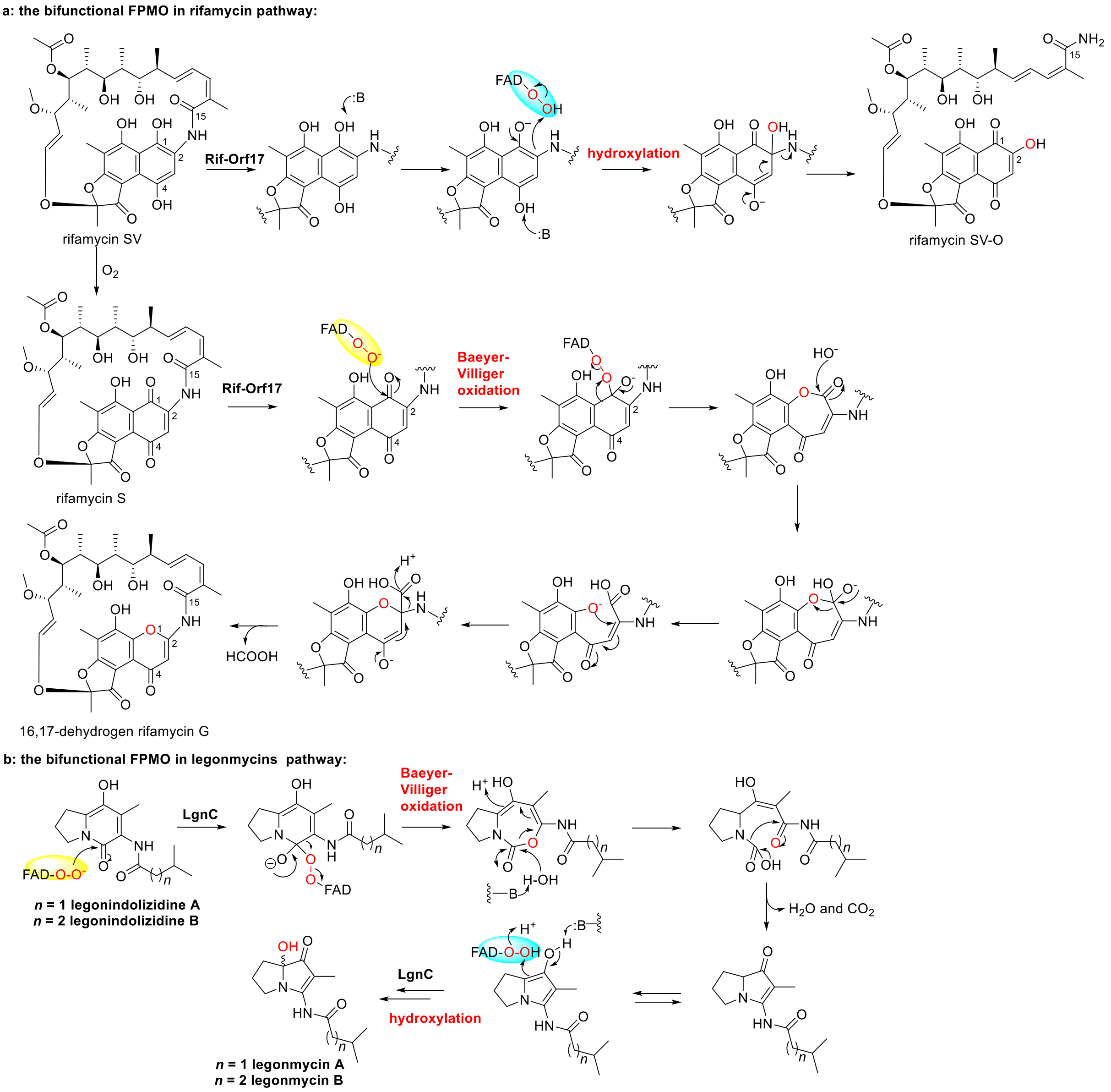

Supplementary Fig. 78. The proposed involvement of two forms of reactive flavin species hydroperoxyflavin (FADOOH) or a peroxyflavin (FADOO$^-$) in a single enzyme-catalyzed reactions in natural product biosynthesis. (**a**) The proposed mechanism of Rif-ORF17 in the biosynthesis of rifamycin. (**b**) The proposed mechanism of LgnC in the biosynthesis of legonmycin.

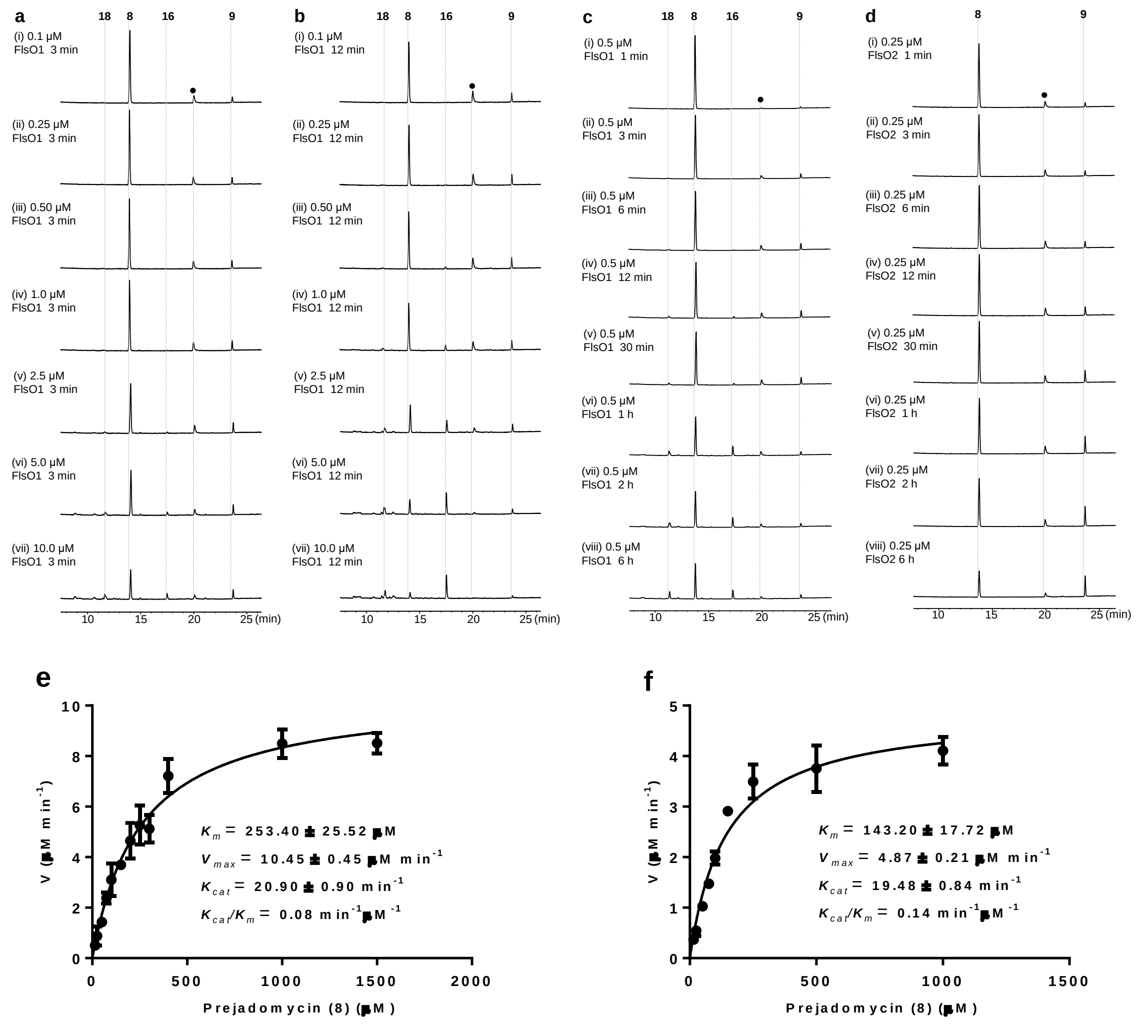

Supplementary Fig. 79. Determination of kinetic parameters of FlsO1 and FlsO2 reactions with PJM (8). (**a**) HPLC analysis of FlsO1 reactions with 50 μM **8** for 3 min in diverse concentrations of FlsO1 ranging from 0.1 to 10 μM. (**b**) HPLC analysis of FlsO1 reactions with 50 μM **8** for 12 min in diverse concentrations of FlsO1 ranging from 0.1 to 10 μM. (**c**) HPLC analysis of a time course reaction of 0.5 μM FlsO1 with 50 μM **8** from 1 min to 6 h. (**d**) HPLC analysis of a time course reaction of 0.25 μM FlsO2 with 50 μM **8** from 1 min to 6 h. (**e**) Determining kinetic parameters of the FlsO1 reaction with **8**. PJM (**8**) was set at 12 concentrations of 15, 25, 50, 75, 100, 150, 200, 250, 300, 400, 1000, and 1500 μM (*n* = 12). At each concentration of **8**, the assays were independently done in triplicates. (**f**) Knitetic parameters of the FlsO2 reaction with **8**. PJM (**8**) was set at 9 concentrations of 15, 25, 50, 75, 100, 150, 250, 500, and 1000 μM (*n* = 9). At each concentration of **8**, the assays were independently done in triplicates. For both (**e**) and (**f**), data are presented as mean values ± SD (standard deviation). Source data are provided as a Source Data file. All reactions were performed in 50 mM PBS buffers (pH 7.0) at 30 °C water bar. The symbol "•" represents an uncharacterized spontaneous product of PJM (**8**). Since very less products could be detected at higher concentrations of the substrate **8** when using FlsO1 lower than 0.5 μM, the FlsO1 assays for measuring the kinetic parameters was performed under the following conditions: diverse concentrations of PJM (**8**) incubated with 0.5 μM FlsO1 and 2 mM NADPH, in 50 mM phosphate buffer (pH 7.0), at 30 °C water bar for 4 min.

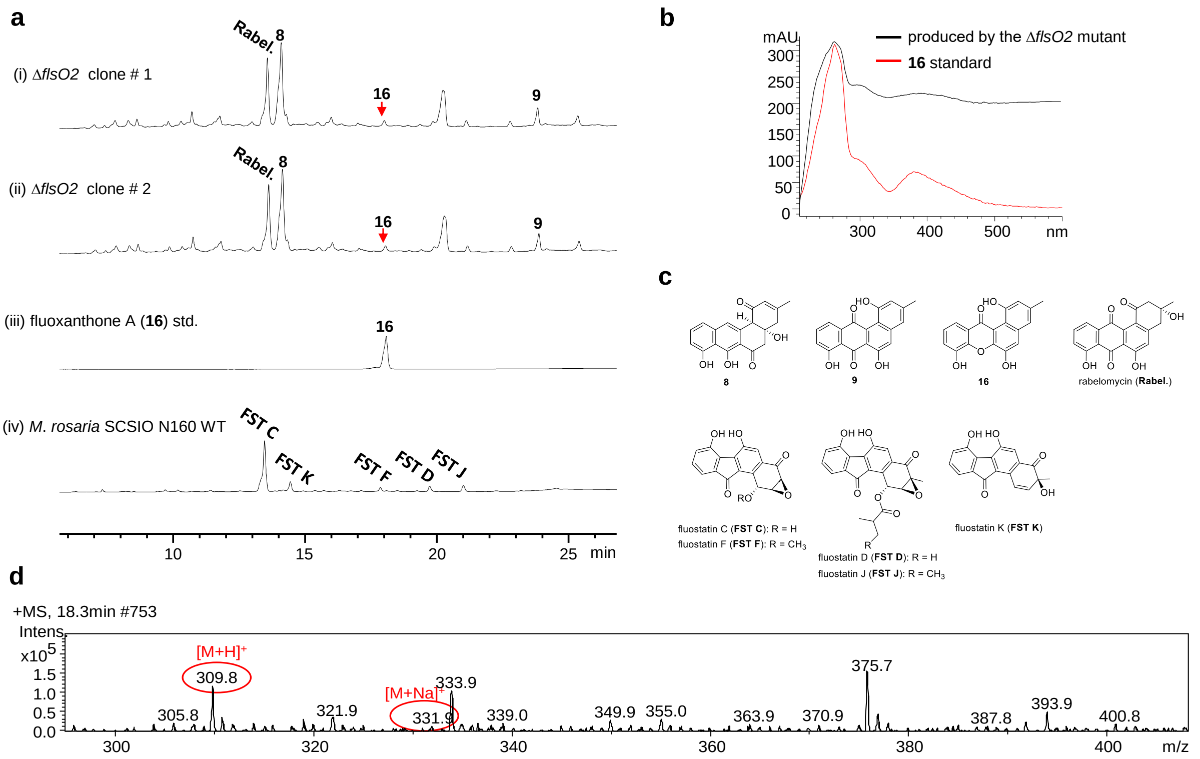

Supplementary Fig. 80. HPLC analysis of the production profiles of *M. rosaria* SCSIO N160 and the Δ*flsO2* mutant. (**a**) Production profiles of the wild type (WT) and the Δ*flsO2* mutant, (i) (Δ*flsO2*) clone #1, (ii) (Δ*flsO2*) clone #2, (iii) fluoxanthone A (**16**) standard, (iv) wild type (WT) strain. (**b**) Characterization of fluoxanthone A (**16**) produced by strain (Δ*flsO2*) via UV spectral comparison with the standard. (**c**) The structures of compounds showing in the HPLC profile of (a). (**d**) Characterization of fluoxanthone A (**16**) produced by the Δ*flsO2* mutant via LC-MS analysis.

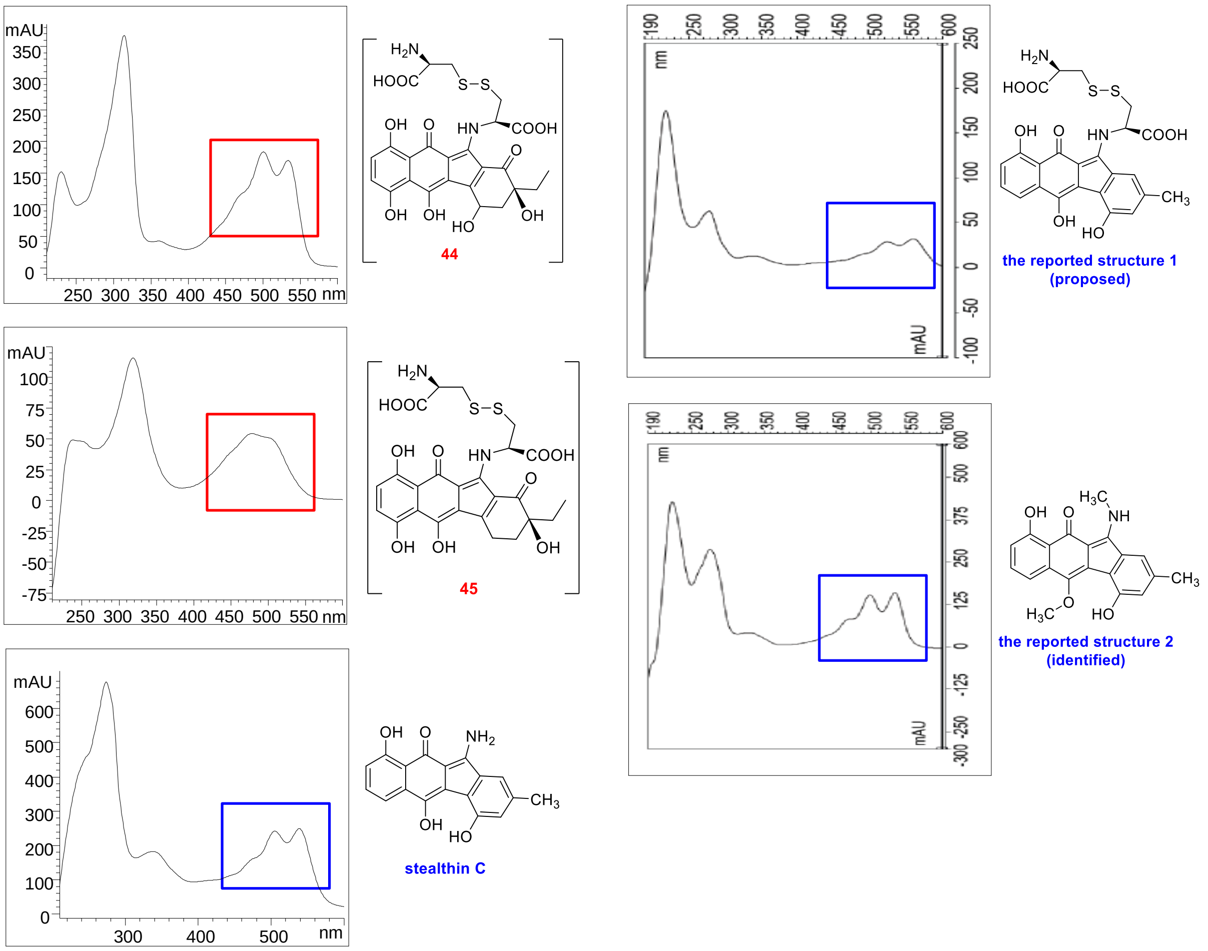

Supplementary Fig. 81. Comparison of UV-Vis spectra of 44 and 45 with those of known compounds. The red marked compounds **44** and **45** were isolated from the up scaled FlsO1-reaction with NEN C (**14**). The blue marked compounds were previously reported by The Balskus's group[7]. Stealthin C was previously identified by our group[8].

a

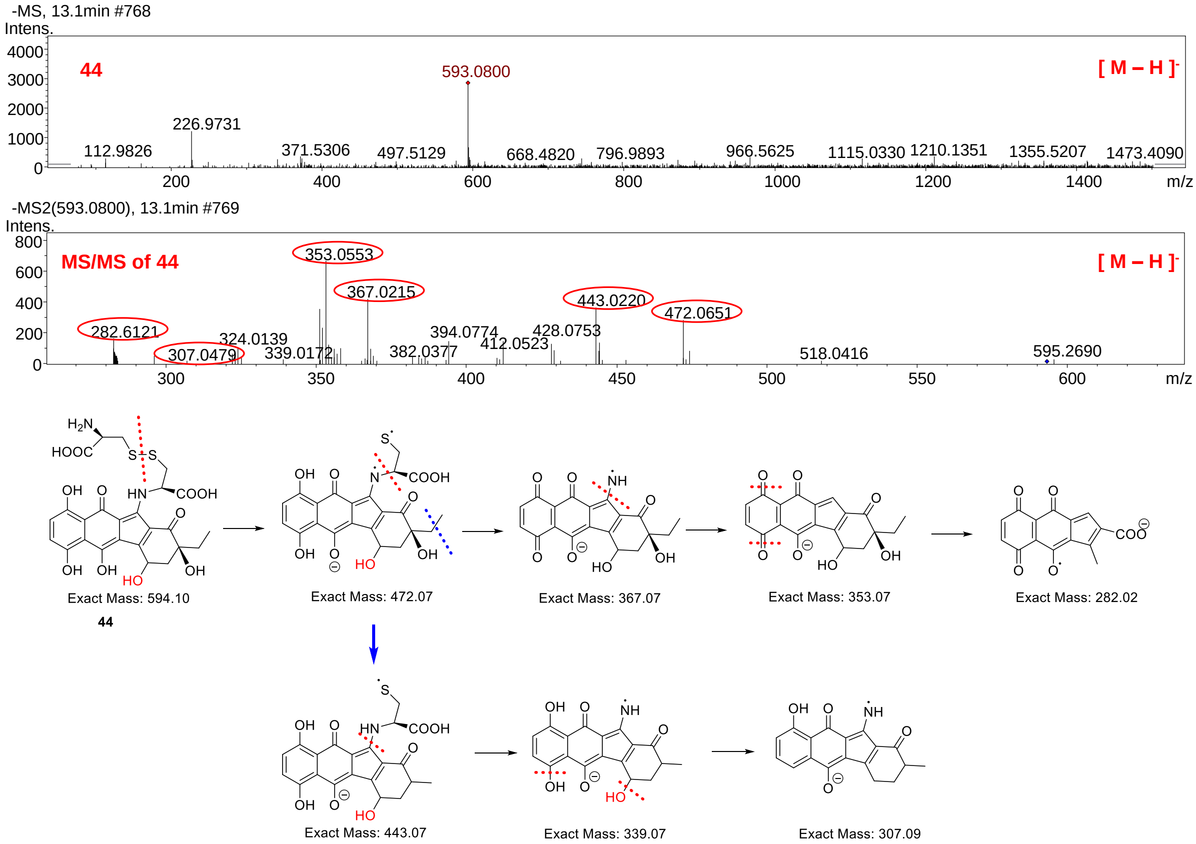

b

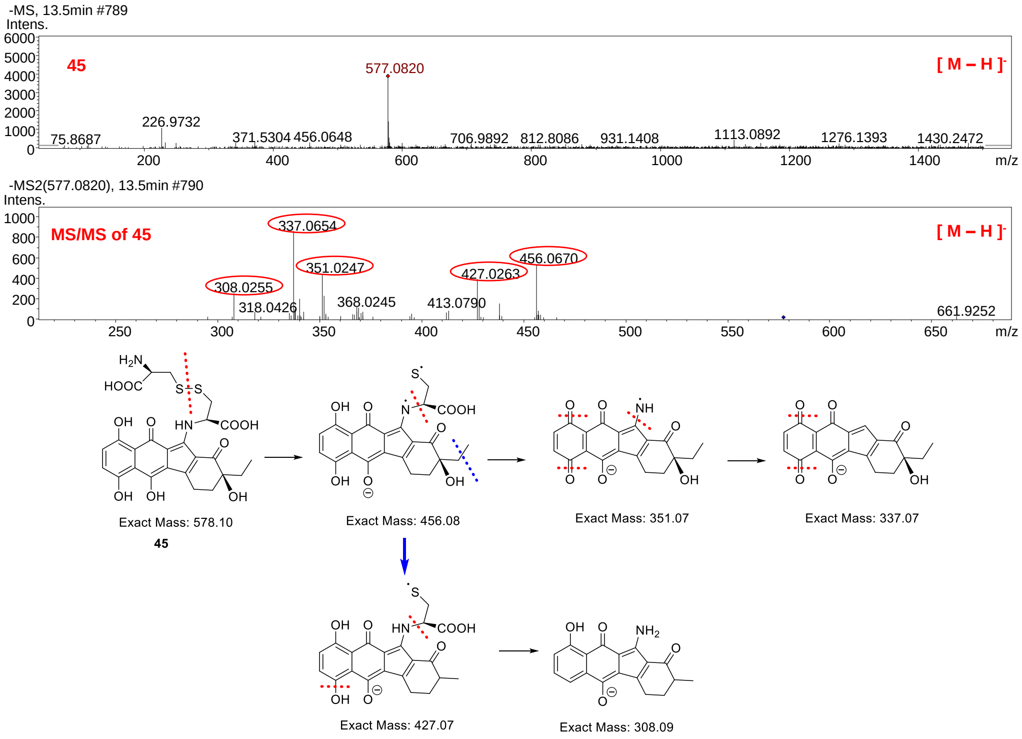

Supplementary Fig. 82. LC-HRMS/MS analysis of 44 and 45. (**a**) HRMS/MS analysis of **44**. (**b**) HRMS/MS analysis of **45**.

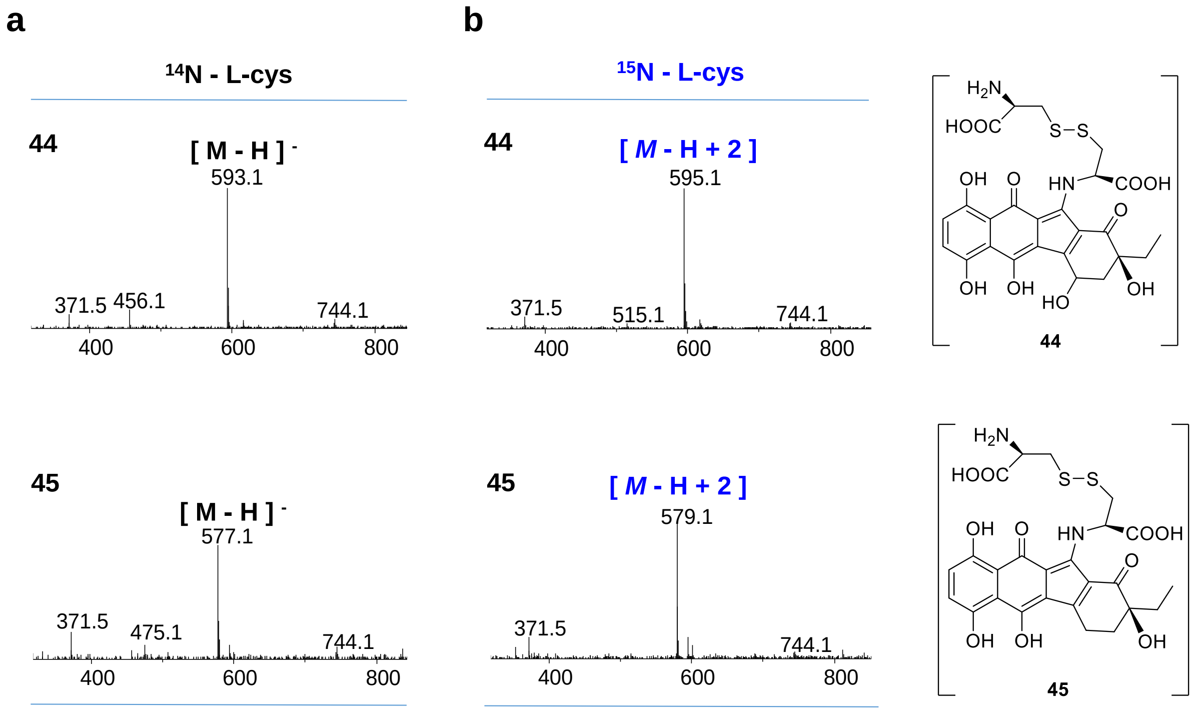

Supplementary Fig. 83. LC-MS analysis of the products 44 and 45 from the FlsO1 reaction with NEN C (14) in the presence of ^{14}N-L-cysteine or ^{15}N-L-cysteine. (**a**) LC-MS analysis of **44** and **45** when the FlsO1 reaction was conducted in the presence of ^{14}N-L-cysteine. (**b**) LC-MS analysis of **44** and **45** when the FlsO1 reaction was conducted in the presence of ^{15}N-L-cysteine.

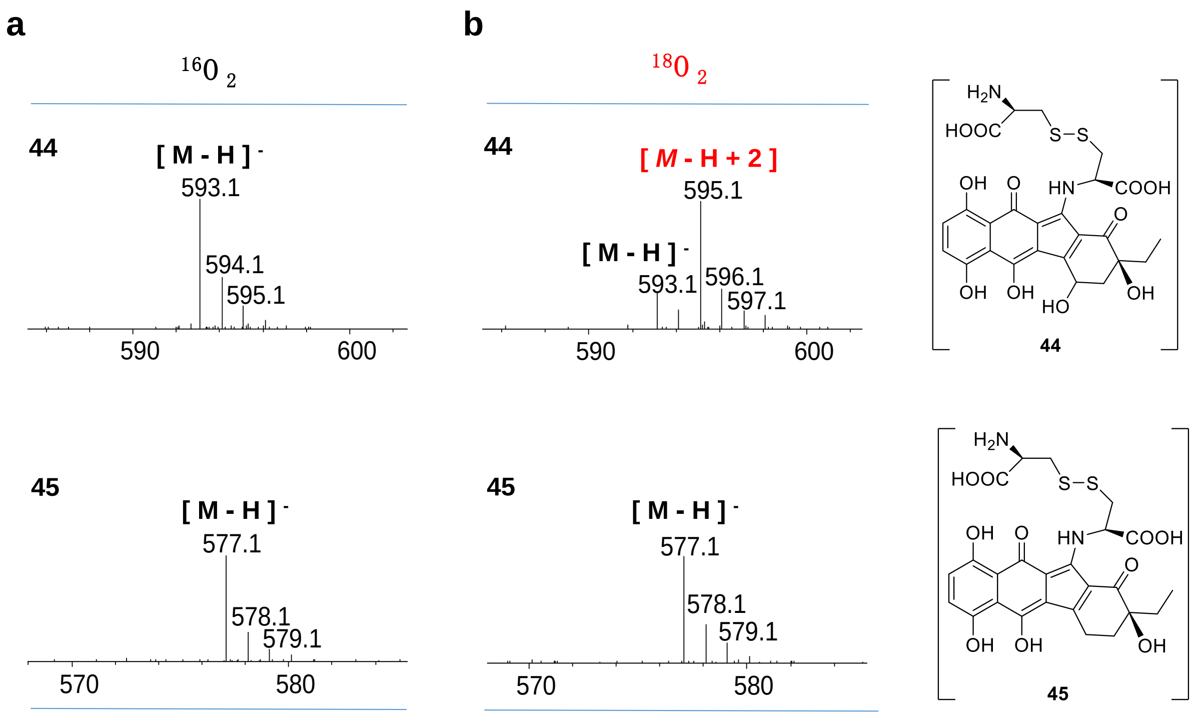

Supplementary Fig. 84. LC-MS analysis of the 44 and 45 of the FlsO1 reaction with 14 in atmospheric $^{16}O_2$ or $^{18}O_2$. (**a**) LC-MS analysis of **44** and **45** when the FlsO1 reaction was conducted in atmospheric $^{16}O_2$. (**b**) LC-MS analysis of **44** and **45** when the FlsO1 reaction was conducted in $^{18}O_2$.

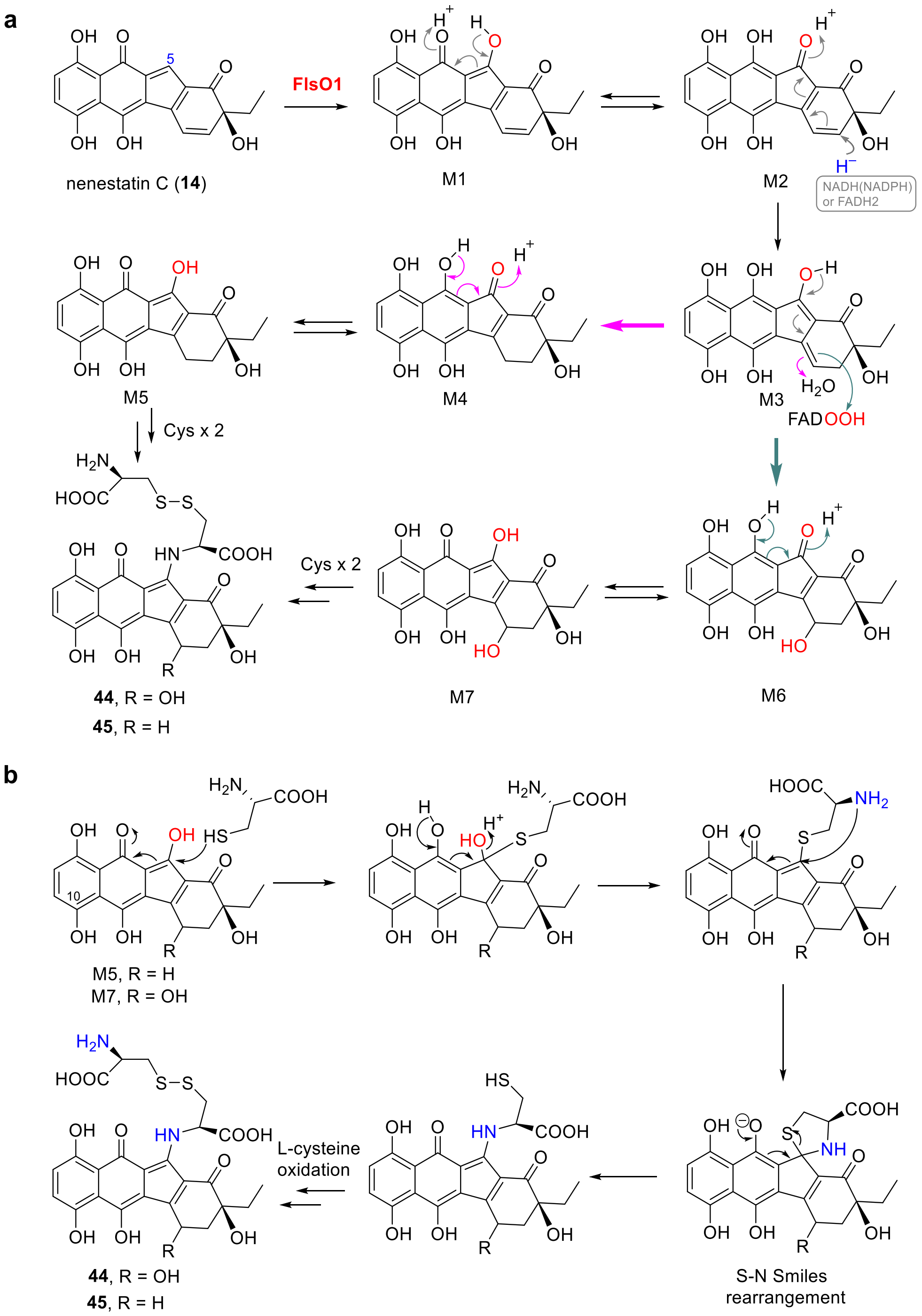

Supplementary Fig. 85. The proposed mechanism for the formation of 44 and 45. (**a**) The speculated FlsO1 reaction mechanism in generation of precursors leading to **44** and **45**. (**b**) The proposed S–N Smiles rearrangement to produce **44** and **45**.[7]

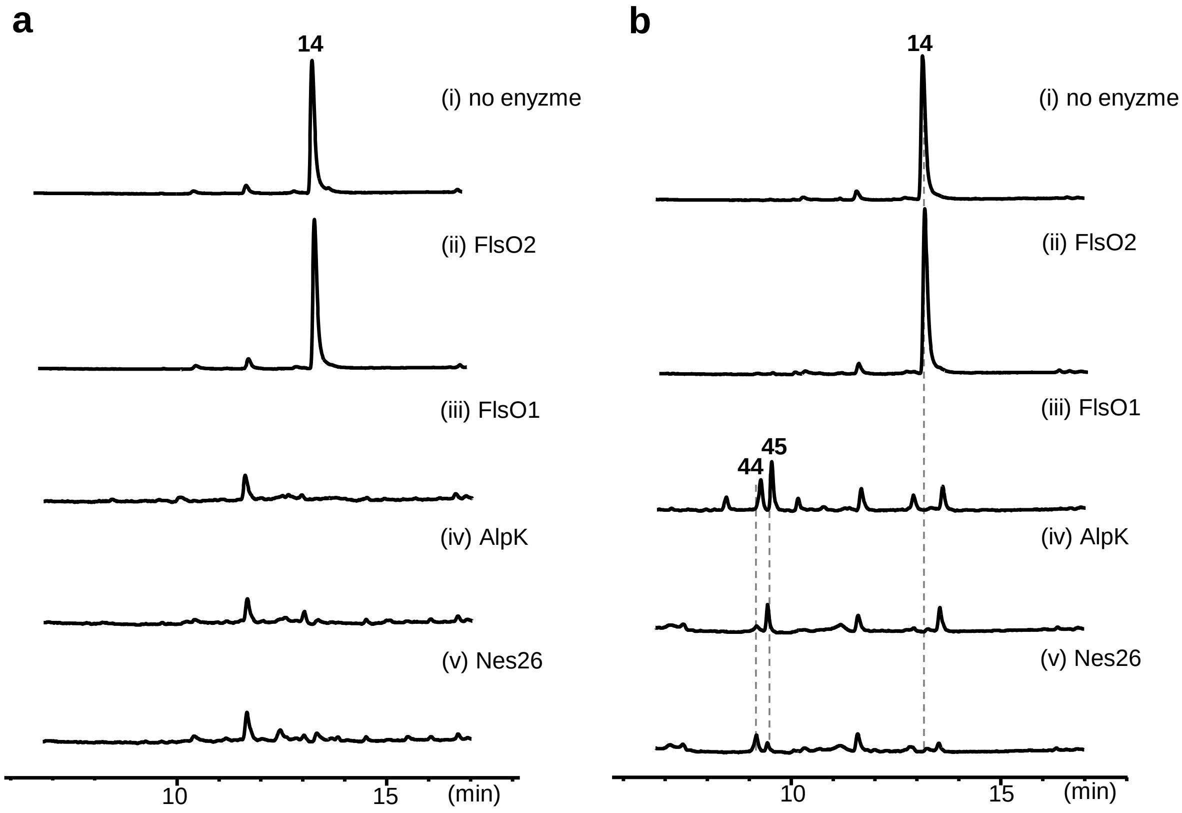

Supplementary Fig. 86. *In vitro* Characterization of the FlsO1 homologous enzymes AlpK and Nes26 using NEN C (14) as a substrate mimic. (**a**) HPLC analysis of enzyme assays. The assays were performed by incubation of 200 μM **14**, 2 mM NADPH, in the absence of L-cysteine, with (i) control (no enzyme); (ii) 10 μM FlsO2; (iii) 10 μM FlsO1; (iv) 10 μM AlpK; (v) 10 μM Nes26. (**b**) HPLC analysis of enzyme assays. The assays were performed by incubation of 200 μM **14**, 2 mM NADPH, in the presence of 4 mM L-cysteine, with (i) control (no enzyme); (ii) 10 μM FlsO2; (iii) 10 μM FlsO1; (iv) 10 μM AlpK; (v) 10 μM Nes26. All the reactions were performed in 50 mM PBS buffers (pH 7.0) at 30 °C for 30 min.

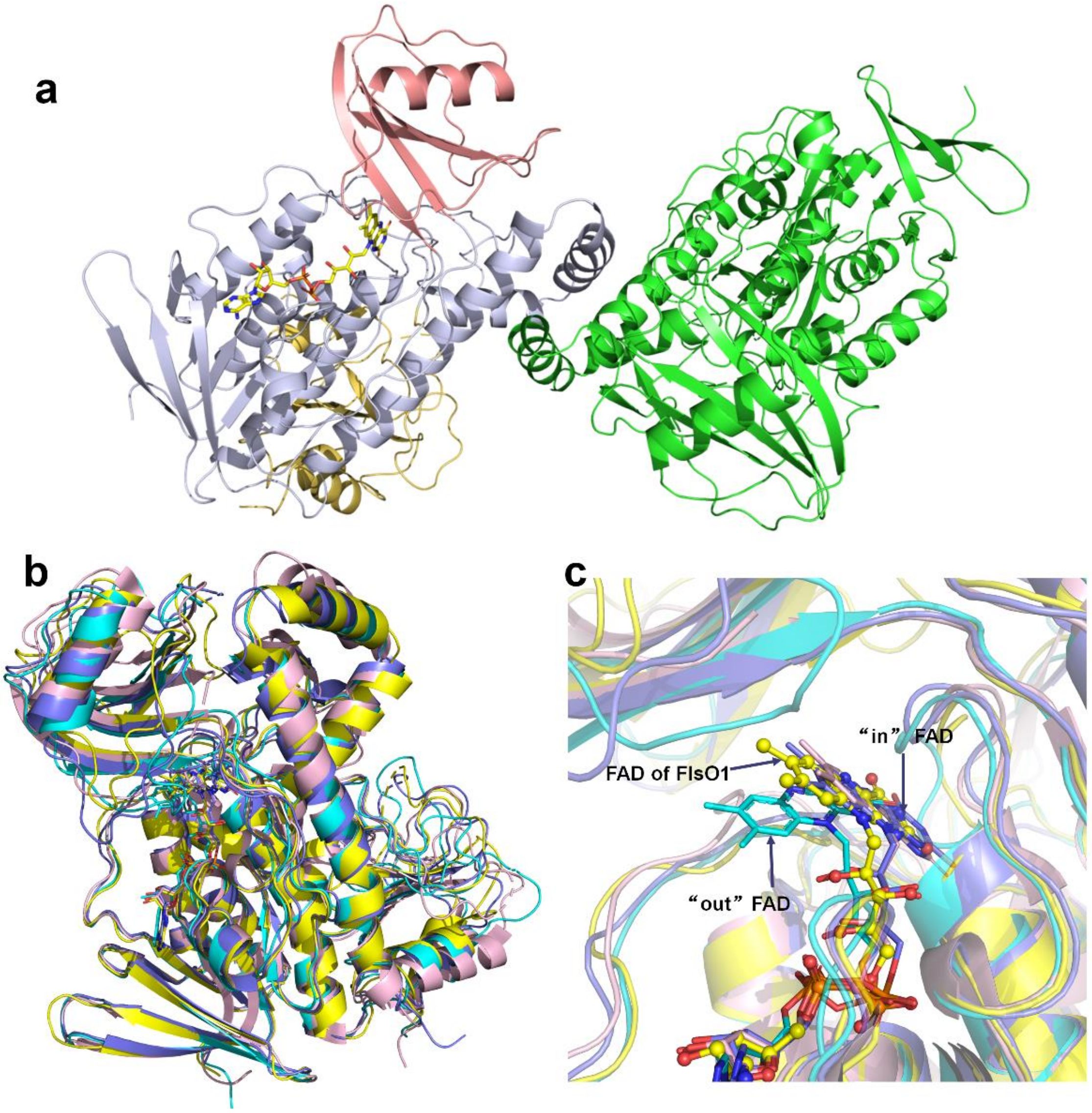

Supplementary Fig. 87. Structure comparison of FlsO1 with its homologues AlpK, MtmOIV, and BexE. (**a**) The homodimer structure of FlsO1. (**b**) FlsO1 (yellow cartoon) aligned well with the typical *para*-hydroxybenzoate hydroxylase (pHBH) subfamily proteins including AlpK (PDB ID: 6J0Z, blue cartoon, RMSD 1.6 Å for 441 atoms), the Baeyer−Villiger monooxygenase MtmOIV (PDB ID: 4k5s, pink, RMSD 0.85 Å for 467 atoms) that catalyzes an oxidative C−C bond cleavage in mithramycin biosynthesis, and the oxygenase BexE (PDB ID: 4x4j, purple, RMSD 1.1 Å for 474 atoms) that mediates oxidative rearrangement reaction during BE-7585A biosynthesis. The structural alignments were conducted using SSM superpose of WinCOOT-0.9.4.1. (**c**) Close-up view of the aligned active sites, with the FAD of AlpK (cyan) in "out" conformation, and the FAD in MtmOIV and BexE in "in" conformation. Note that FAD of FlsO1 close to the "in" conformation.

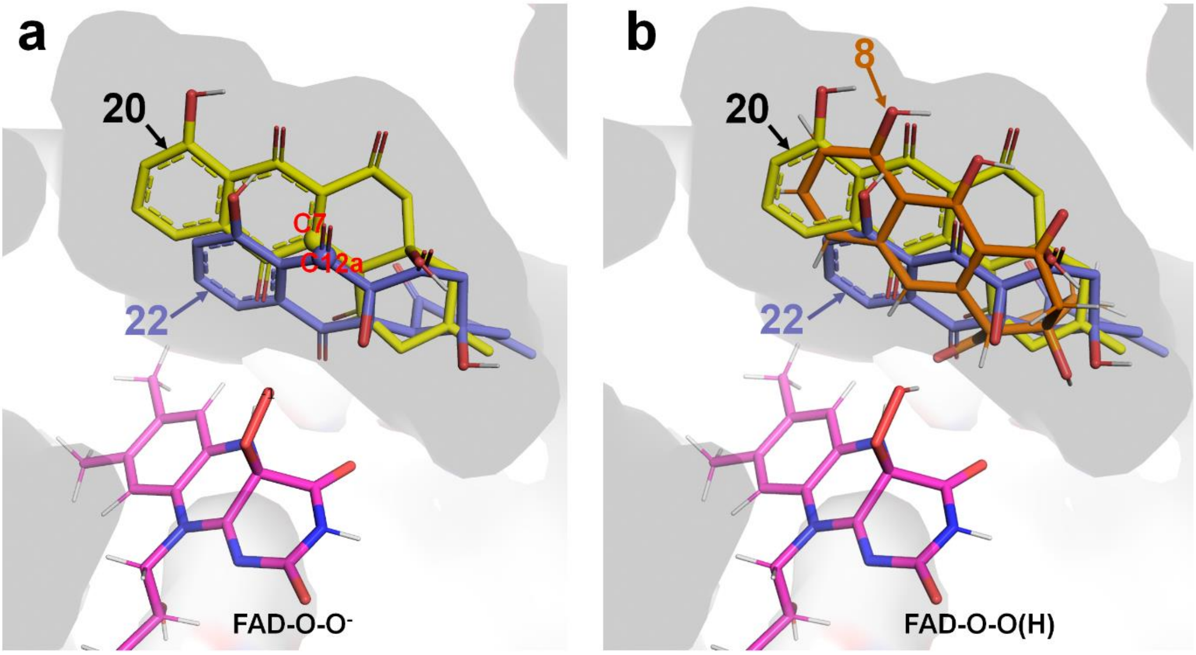

Supplementary Fig. 88. Alignments of the docked models of 8, 20 and 22. (**a**) **20** (yellow stick) was aligned with **22** (lightblue stick). Note that C12a atom of **20** overlays with C7 atom of **22**, consistent with that the two carbons are proposed to be attacked by the peroxyflavin FAD-O-O$^-$ (magenta stick). (**b**) The alignments of all three docked models of **8** (orange stick), **20** (yellow stick) and **22** (lightblue stick) reveal similar orientations in the active pocket, suggesting that the unusual stepwise oxidations on **8**, **20** and **22** only require slight swings of the intermediates in the active site of FlsO1.

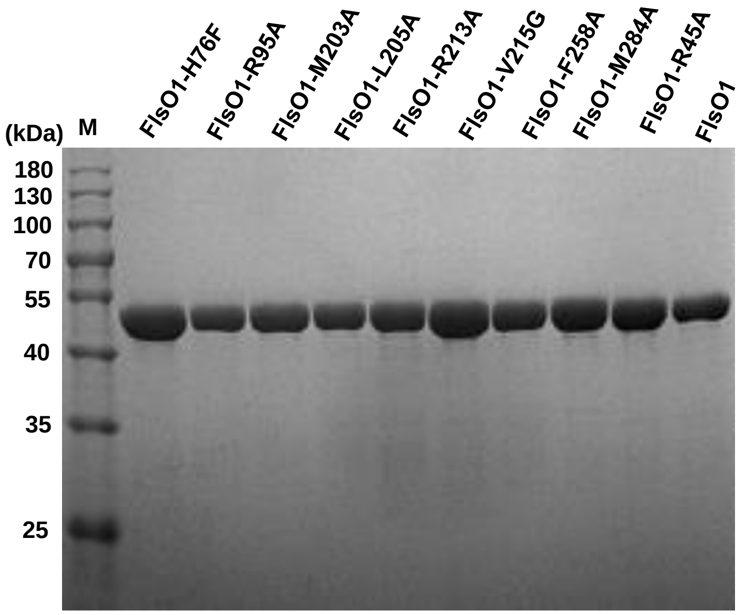

Supplementary Fig. 89. The SDS-PAGE analysis of purified recombinant proteins of FlsO1 and its mutants. Lane M, protein molecular weight marker (PageRuler). The acrylamide percentage of SDS-PAGE gel is 12%. Each experiment was independently repeated two times.

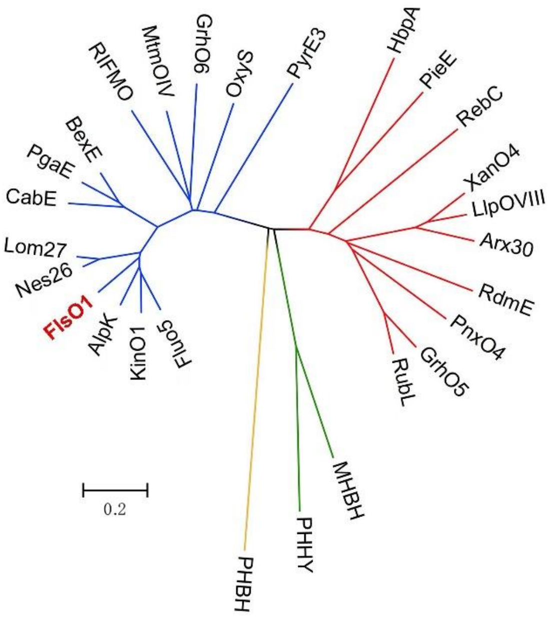

Supplementary Fig. 90. Phylogenetic analysis of FlsO1. The tree was constructed by Neighbor-Joining method using MEGA 3.1.[5] Scale bar represents 20% dissimilarity. The amino acid sequences are consisted of the entire group A FPMOs Information of the reference sequences are as follows: FlsO1 (*Micromonospora rosaria*, GenBank: ALJ99859.1); Nes26 (*Micromonospora echinospora*, GenBank: ARD70875.1); Lom27 (*Salinispora pacifica*, GenBank: AHZ61861.1); Fluo5 (*Streptomyces sp*. PKU-MA00045); AlpK (*Streptomyces ambofaciens* ATCC 23877, GenBank: AKZ60515.1); KinO1 (*Streptomyces murayamaensis*, GenBank: AAO65343.1); XanO4 (*Streptomyces flavogriseus*, GenBank: ADE22300.1); RebC (*Lentzea aerocolonigenes,* GenBank: BAC15752.1); MtmOIV (*Streptomyces argillaceus*, GenBank: CAK50794.2); GrhO5 (*Streptomyces sp*. JP95, GenBank: AAM33672.1); Arx30 (uncultured bacterium, GenBank: AHX24715.1); LlpOVIII (*Streptomyces tendae*, GenBank: CAM34363.1); PnxO4 (*Streptomyces sp*. TA-0256, GenBank: BAJ52694.1); CabE (*Streptomyces sp*. H021, PDB: 2QA2); PgaE (*Streptomyces sp*. PGA64, PDB: 4ICY); BexE (*Amycolatopsis orientalis subsp*. *Vinearia*, GenBank: ADI71441.1); OxyS (*Streptomyces rimosus*, GenBank: AAZ78342.1); PHHY (*Trichosporon cutaneum*,UniProtKB/Swiss-Prot: P15245.3); MHBH (*Comamonas testosteroni*, PDB: 2DKI_A); PHBH (*Pseudomonas fluorescens*, PDB: 1PDH); HbpA (*Pseudomonas nitroreducens*, GenBank: QIE86093.1); PieE (*Streptomyces sp*. SCSIO 3032, GenBank: AHE81001.1); RdmE (*Streptomyces purpurascens*, GenBank: AAA83424.1); RubL (*Streptomyces collinus*, GenBank: AAM97362.1); PyrE3 (*Streptomyces rugosporus*, GenBank: AFV71312.1); RIFMO (*Streptomyces venezuelae* ATCC 10712, UniProtKB/Swiss-Prot: F2R776.1); GrhO6 (*Streptomyces sp*. JP95, GenBank: AAM33673.1) .

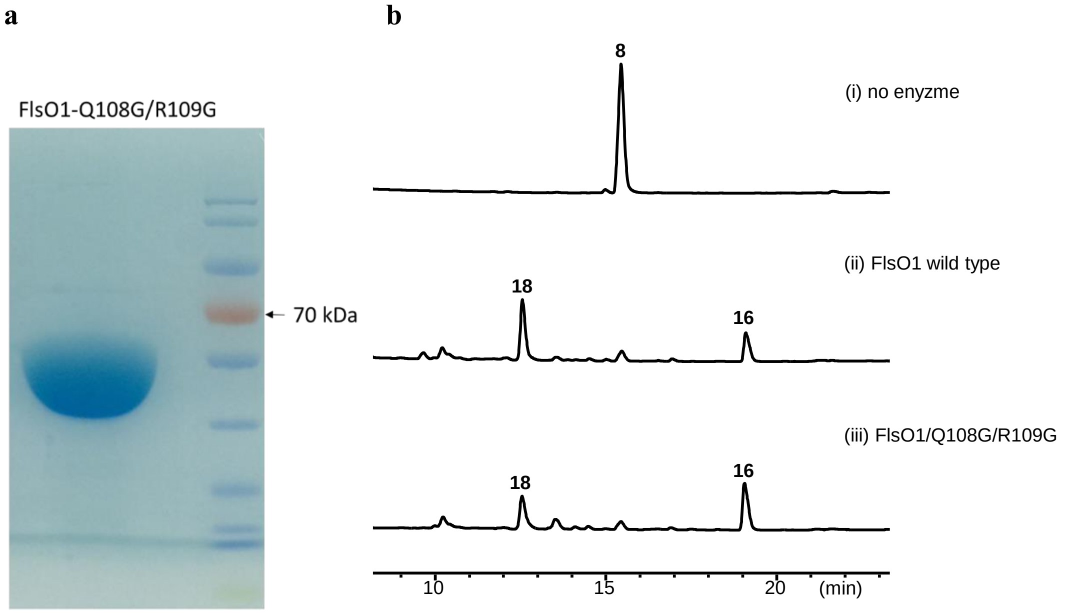

Supplementary Fig. 91. Characterization of the catalytic activity of FlsO1 Q108G/R109G mutant. (**a**) The SDS-PAGE analysis of purified recombinant proteins of FlsO1 Q108G/R109G mutant with the protein molecular weight marker (PageRuler) and the same experiment was independently performed two times. The acrylamide percentage of SDS-PAGE gel is 12%. (**b**) HPLC analysis of the biochemical assays. The assays were performed by incubation of 200 μM PJM (**8**) in the presence of 2 mM NADPH, with (i) control (no enzyme); (ii) 10 μM FlsO1; (iii) 10 μM FlsO1 Q108G/R109G. The reactions were performed in 50 mM PBS buffers (pH 7.0) at 30 °C for 30 min.

Supplementary References

1. Yang, C., Huang, C., Zhang, W., Zhu, Y. & Zhang, C. Heterologous Expression of Fluostatin Gene Cluster Leads to a Bioactive Heterodimer. *Org. Lett.* **17**, 5324-5327 (2015).
2. Zhang, W*., et al.* Fluostatins I-K from the South China Sea-derived *Micromonospora rosaria* SCSIO N160. *J. Nat. Prod.* **75**, 1937-1943 (2012).
3. Jiang, X*., et al.* Isolation, structure elucidation and biosynthesis of benzo[b]fluorene nenestatin A from deep-sea derived *Micromonospora echinospora* SCSIO 04089. *Tetrahedron* **73**, 3585-3590 (2017).
4. Jiang, X*., et al.* Discovery of a new asymmetric dimer nenestatin B and implications of a dimerizing enzyme in a deep sea actinomycete. *Org. Biomol. Chem.* **19**, 4243-4247 (2021).
5. Wang, B*., et al.* Kinamycin biosynthesis employs a conserved pair of oxidases for B-ring contraction. *Chem. Commun.* **51**, 8845-8848 (2015).
6. Resende, D. I. S. P., Durães, F., Maia, M., Sousa, E. & Pinto, M. M. M. Recent advances in the synthesis of xanthones and azaxanthones. *Org. Chem. Front.* **7**, 3027-3066 (2020).
7. Wang, P., Hong, G. J., Wilson, M. R. & Balskus, E. P. Production of Stealthin C Involves an S-N-Type Smiles Rearrangement. *J. Am. Chem. Soc.* **139**, 2864-2867 (2017).
8. Huang, C*., et al.* Discovery of Stealthin Derivatives and Implication of the Amidotransferase FlsN3 in the Biosynthesis of Nitrogen-Containing Fluostatins. *Mar. Drugs* **17**, 150 (2019).
