# Supplementary material for: Biochemical and structural insights of multifunctional flavin-dependent monooxygenase FlsO1-catalyzed unexpected xanthone formation
Source: Nat Commun. 2022 Sep 14;13:5386. doi: 10.1038/s41467-022-33131-0 (PMC9474520; doi:10.1038/s41467-022-33131-0)
Supplement: Supplementary file 3 — Reporting Summary [file 41467_2022_33131_MOESM3_ESM.pdf]

nature portfolio

Corresponding author(s): Changsheng Zhang
Last updated by author(s): Aug 29, 2022

Reporting Summary

Nature Portfolio wishes to improve the reproducibility of the work that we publish. This form provides structure for consistency and transparency in reporting. For further information on Nature Portfolio policies, see our Editorial Policies and the Editorial Policy Checklist.

Statistics

For all statistical analyses, confirm that the following items are present in the figure legend, table legend, main text, or Methods section.

n/a	Confirmed	
☐	☒	The exact sample size (n) for each experimental group/condition, given as a discrete number and unit of measurement
☐	☒	A statement on whether measurements were taken from distinct samples or whether the same sample was measured repeatedly
☐	☒	The statistical test(s) used AND whether they are one- or two-sided<br>*Only common tests should be described solely by name; describe more complex techniques in the Methods section.*
☐	☒	A description of all covariates tested
☐	☒	A description of any assumptions or corrections, such as tests of normality and adjustment for multiple comparisons
☐	☒	A full description of the statistical parameters including central tendency (e.g. means) or other basic estimates (e.g. regression coefficient) AND variation (e.g. standard deviation) or associated estimates of uncertainty (e.g. confidence intervals)
☒	☐	For null hypothesis testing, the test statistic (e.g. F, t, r) with confidence intervals, effect sizes, degrees of freedom and P value noted<br>*Give P values as exact values whenever suitable.*
☒	☐	For Bayesian analysis, information on the choice of priors and Markov chain Monte Carlo settings
☐	☒	For hierarchical and complex designs, identification of the appropriate level for tests and full reporting of outcomes
☒	☐	Estimates of effect sizes (e.g. Cohen's d, Pearson's r), indicating how they were calculated

Our web collection on statistics for biologists contains articles on many of the points above.

Software and code

Policy information about availability of computer code

Data collection	The crystal data were collected using the in-house Rigaku XtaLAB Pro: kappa single device equipped with rotating anode X-ray source (λCu Kα = 1.54184 Å) and Pilatus 3R 200K-A detector and processed using CrysAlisPro version 1.0.39.
Data analysis	The software packages PHENIX and CCP4 were used for crystal structure solving and refinement. To improve the diffraction quality, the double mutation of two surface residues of FlsO1 (Q108G/R109G) was designed by the Surface Entropy Reduction prediction (SERp) server. The qualities of the final model were validated by MolProbity. PDBePISA and POCASA online tools are also used for structure analysis. Autodock vina, Grade Web Server and the Desmond package of Schrödinger software (Schrodinger LLC. 2020) were used for the substrates docking and MD simulations studies of FlsO1. Structural diagrams were prepared using the program PyMOL. Kinetic parameters (Km, kcat, Vmax) were determined by nonlinear regression analysis using the GraphPad Prism 6 software. The phylogenetic tree was generated using MEGA 3.1 through application of the neighbor-joining method.

For manuscripts utilizing custom algorithms or software that are central to the research but not yet described in published literature, software must be made available to editors and reviewers. We strongly encourage code deposition in a community repository (e.g. GitHub). See the Nature Portfolio guidelines for submitting code & software for further information.

Data

Policy information about availability of data

All manuscripts must include a data availability statement. This statement should provide the following information, where applicable:

- Accession codes, unique identifiers, or web links for publicly available datasets
- A description of any restrictions on data availability
- For clinical datasets or third party data, please ensure that the statement adheres to our policy

Data generated in this study are available within the paper and its Supplementary information files. The GenBank accession number of fls genes (flsO1, flsO2, flsO3, flsO4 and flsO5) is KT726162.1 [https://www.ncbi.nlm.nih.gov/nuccore/KT726162.1]. The GenBank accession number of alpK is AY338477.2 [https://www.ncbi.nlm.nih.gov/nuccore/AY338477.2]. The GenBank accession number of nes26 is KY454837.1 [https://www.ncbi.nlm.nih.gov/nuccore/KY454837.1]. Crystallographic data for FlsO1 were deposited in the Protein Data Bank (PDB) with accession codes 7VWP [http://doi.org/10.2210/pdb7VWP/pdb]. The structures were obtained from the Protein Data Bank (PDB) with accession codes 6J0Z (AlpK) [http://doi.org/10.2210/pdb6J0Z/pdb], 2QA1 (PgaE) [http://doi.org/10.2210/pdb2QA1/pdb], 2QA2 (CabE) [http://doi.org/10.2210/pdb2QA2/pdb], 4K5S (MtmOIV) [http://doi.org/10.2210/pdb4K5S/pdb], and 4X4J (BexE) [http://doi.org/10.2210/pdb4X4J/pdb]. Source data are provided with this paper. Data is available from the corresponding authors upon request. Data for this manuscript are also available at South China Sea Ocean Data Center, National Earth System Science Data Center, National Science & Technology Infrastructure of China [http://data.scsio.ac.cn/metaData-detail/1563148765659291648].

Field-specific reporting

Please select the one below that is the best fit for your research. If you are not sure, read the appropriate sections before making your selection.

☒ Life sciences ☐ Behavioural & social sciences ☐ Ecological, evolutionary & environmental sciences

For a reference copy of the document with all sections, see nature.com/documents/nr-reporting-summary-flat.pdf

Life sciences study design

All studies must disclose on these points even when the disclosure is negative.

Sample size	For determining kinetic parameters of FlsO1, PJM (8) was set at 12 concentrations of 15, 25, 50, 75, 100, 150, 200, 250, 300, 400, 1000, and 1500 µM (n = 12); For determining the knitetic parameters of the FlsO2, PJM (8) was set at 9 concentrations of 15, 25, 50, 75, 100, 150, 250, 500, and 1000 µM (n = 9).
Data exclusions	No data were excluded from the analyses.
Replication	All data were repeatable and reproducible. For determining kinetic parameters of FlsO1-catalyzed reaction, PJM (8) was set at the concentrations of 15, 25, 50, 75, 100, 150, 200, 250, 300, 400, 1000, and 1500 µM. Enzyme assays were performed in triplicates in 50 mM phosphate buffer (pH 7.0) with 0.5 µM FlsO1 and 2 mM NADPH, by incubation at 30 °C for 4 min. For determining kinetic parameters of FlsO2-catalyzed reaction, PJM (8) was set at the concentrations of 15, 25, 50, 75, 100, 150, 250, 500, and 1000 µM. Enzyme assays were performed in triplicates in 50 mM phosphate buffer (pH 7.0) with 0.25 µM FlsO2 and 2 mM NADPH, by incubation at 30 °C for 6 min.
Randomization	This is not relevant to our study, because our study belongs to the field of biochemistry focusing on the catalytic function and mechanism of specific enzymes.
Blinding	Non-applicable to this study, because this study is on characterizing the oxygenase FlsO1, which belongs to biochemical work and dose not require blinding.

Behavioural & social sciences study design

All studies must disclose on these points even when the disclosure is negative.

Study description	Non-applicable to this study.
Research sample	Non-applicable to this study.
Sampling strategy	Non-applicable to this study.
Data collection	Non-applicable to this study.
Timing	Non-applicable to this study.
Data exclusions	Non-applicable to this study.
Non-participation	Non-applicable to this study.

Randomization	Non-applicable to this study.

Ecological, evolutionary & environmental sciences study design

All studies must disclose on these points even when the disclosure is negative.

Study description	Non-applicable to this study.
Research sample	Non-applicable to this study.
Sampling strategy	Non-applicable to this study.
Data collection	Non-applicable to this study.
Timing and spatial scale	Non-applicable to this study.
Data exclusions	Non-applicable to this study.
Reproducibility	Non-applicable to this study.
Randomization	Non-applicable to this study.
Blinding	Non-applicable to this study.

Did the study involve field work? ☐ Yes ☒ No

Reporting for specific materials, systems and methods

We require information from authors about some types of materials, experimental systems and methods used in many studies. Here, indicate whether each material, system or method listed is relevant to your study. If you are not sure if a list item applies to your research, read the appropriate section before selecting a response.

Materials & experimental systems

n/a	Involved in the study
☒	☐ Antibodies
☒	☐ Eukaryotic cell lines
☒	☐ Palaeontology and archaeology
☒	☐ Animals and other organisms
☒	☐ Human research participants
☒	☐ Clinical data
☒	☐ Dual use research of concern

Methods

n/a	Involved in the study
☒	☐ ChIP-seq
☒	☐ Flow cytometry
☒	☐ MRI-based neuroimaging

Antibodies

Antibodies used	Non-applicable to this study.
Validation	Non-applicable to this study.

Eukaryotic cell lines

Policy information about cell lines

Cell line source(s)	Non-applicable to this study.
Authentication	Non-applicable to this study.
Mycoplasma contamination	Non-applicable to this study.
Commonly misidentified lines (See ICLAC register)	Non-applicable to this study.

Palaeontology and Archaeology

Specimen provenance	Non-applicable to this study.

Specimen deposition | Non-applicable to this study.

Dating methods | Non-applicable to this study.

☐ Tick this box to confirm that the raw and calibrated dates are available in the paper or in Supplementary Information.

Ethics oversight | Non-applicable to this study.

Note that full information on the approval of the study protocol must also be provided in the manuscript.

Animals and other organisms

Policy information about studies involving animals; ARRIVE guidelines recommended for reporting animal research

Laboratory animals	Non-applicable to this study.
Wild animals	Non-applicable to this study.
Field-collected samples	Non-applicable to this study.
Ethics oversight	Non-applicable to this study.

Note that full information on the approval of the study protocol must also be provided in the manuscript.

Human research participants

Policy information about studies involving human research participants

Population characteristics	Non-applicable to this study.
Recruitment	Non-applicable to this study.
Ethics oversight	Non-applicable to this study.

Note that full information on the approval of the study protocol must also be provided in the manuscript.

Clinical data

Policy information about clinical studies

All manuscripts should comply with the ICMJEguidelines for publication of clinical research and a completedCONSORT checklist must be included with all submissions.

Clinical trial registration	Non-applicable to this study.
Study protocol	Non-applicable to this study.
Data collection	Non-applicable to this study.
Outcomes	Non-applicable to this study.

Dual use research of concern

Policy information about dual use research of concern

Hazards

Could the accidental, deliberate or reckless misuse of agents or technologies generated in the work, or the application of information presented in the manuscript, pose a threat to:

No	Yes	
☒	☐	Public health
☒	☐	National security
☒	☐	Crops and/or livestock
☒	☐	Ecosystems
☒	☐	Any other significant area

Experiments of concern

Does the work involve any of these experiments of concern:

No	Yes	
☒	☐	Demonstrate how to render a vaccine ineffective
☒	☐	Confer resistance to therapeutically useful antibiotics or antiviral agents
☒	☐	Enhance the virulence of a pathogen or render a nonpathogen virulent
☒	☐	Increase transmissibility of a pathogen
☒	☐	Alter the host range of a pathogen
☒	☐	Enable evasion of diagnostic/detection modalities
☒	☐	Enable the weaponization of a biological agent or toxin
☒	☐	Any other potentially harmful combination of experiments and agents

ChIP-seq

Data deposition

☐ Confirm that both raw and final processed data have been deposited in a public database such as GEO.

☐ Confirm that you have deposited or provided access to graph files (e.g. BED files) for the called peaks.

Data access links<br>*May remain private before publication.*	Non-applicable to this study.
Files in database submission	Non-applicable to this study.
Genome browser session<br>(e.g. UCSC)	Non-applicable to this study.

Methodology

Replicates	Non-applicable to this study.
Sequencing depth	Non-applicable to this study.
Antibodies	Non-applicable to this study.
Peak calling parameters	Non-applicable to this study.
Data quality	Non-applicable to this study.
Software	Non-applicable to this study.

Flow Cytometry

Plots

Confirm that:

☐ The axis labels state the marker and fluorochrome used (e.g. CD4-FITC).

☐ The axis scales are clearly visible. Include numbers along axes only for bottom left plot of group (a 'group' is an analysis of identical markers).

☐ All plots are contour plots with outliers or pseudocolor plots.

☐ A numerical value for number of cells or percentage (with statistics) is provided.

Methodology

Sample preparation	Non-applicable to this study.
Instrument	Non-applicable to this study.
Software	Non-applicable to this study.
Cell population abundance	Non-applicable to this study.

Gating strategy | Non-applicable to this study.

☐ Tick this box to confirm that a figure exemplifying the gating strategy is provided in the Supplementary Information.

Magnetic resonance imaging

Experimental design

Design type	Non-applicable to this study.
Design specifications	Non-applicable to this study.
Behavioral performance measures	Non-applicable to this study.

Acquisition

Imaging type(s)	Non-applicable to this study.
Field strength	Non-applicable to this study.
Sequence & imaging parameters	Non-applicable to this study.
Area of acquisition	Non-applicable to this study.

Diffusion MRI ☐ Used ☒ Not used

Preprocessing

Preprocessing software	Non-applicable to this study.
Normalization	Non-applicable to this study.
Normalization template	Non-applicable to this study.
Noise and artifact removal	Non-applicable to this study.
Volume censoring	Non-applicable to this study.

Statistical modeling & inference

Model type and settings	Non-applicable to this study.
Effect(s) tested	Non-applicable to this study.

Specify type of analysis: ☐ Whole brain ☐ ROI-based ☐ Both

Statistic type for inference (See Eklund et al. 2016)	Non-applicable to this study.
Correction	Non-applicable to this study.

Models & analysis

n/a	Involved in the study
☒	☐ Functional and/or effective connectivity
☒	☐ Graph analysis
☒	☐ Multivariate modeling or predictive analysis
